# Study on the Polar Extracts of *Dendrobium nobile*, *D. officinale*, *D. loddigesii*, and *Flickingeria fimbriata*: Metabolite Identification, Content Evaluation, and Bioactivity Assay

**DOI:** 10.3390/molecules23051185

**Published:** 2018-05-15

**Authors:** Huiping Chen, Xuewen Li, Yongli Xu, Kakei Lo, Huizhen Zheng, Haiyan Hu, Jun Wang, Yongcheng Lin

**Affiliations:** 1 School of Pharmaceutical Sciences, Sun Yat-sen University, Guangzhou 510006, China; chenhp25@mail2.sysu.edu.cn (H.C.); lixw28@mail2.sysu.edu.cn (X.L.); xuyl7@mail2.sysu.edu.cn (Y.X.); lokk@mail2.sysu.edu.cn (K.L.); shirleyzheng1126@gmail.com (H.Z.); 2 School of Chemistry and Chemical Engineering, Sun Yat-sen University, Guangzhou 510275, China; ceslyc@mail.sysu.edu.cn

**Keywords:** *Dendrobium*, *Flickingeria fimbriata*, qNMR, α-glucosidase inhibition, antioxygenation, neuroprotection

## Abstract

The polar extract of the *Dendrobium* species or *F. fimbriata* (a substitute of *Dendrobium*), between the fat-soluble extract and polysaccharide has barely been researched. This report worked on the qualitative and quantitative studies of polar extracts from *D. nobile*, *D. officinale*, *D. loddigesii*, and *F. fimbriata*. Eight water-soluble metabolites containing a new diglucoside, flifimdioside A (**1**), and a rare imidazolium-type alkaloid, anosmine (**4**), were identified using chromatography as well as spectroscopic techniques. Their contents in the four herbs were high, approximately 0.9–3.7 mg/g based on the analysis of quantitative nuclear magnetic resonance (qNMR) spectroscopy. Biological activity evaluation showed that the polar extract of *F. fimbriata* or its pure component had good antioxidant and neuroprotective activity; compounds **1**‒**4** and shihunine (**8**) showed weak α-glucosidase inhibitory activity; **4** and **8** had weak anti-inflammatory activity. Under trial conditions, all samples had no cytotoxic activity.

## 1. Introduction

“Shi Hu” is the general name for *Dendrobium* Sw. (*Orchidaceae*) and *F. fimbriata* (*Orchidaceae*; a substitute of *Dendrobium*) in China; it has been used as a tonic in both medicine and food since ancient times [1,2]. The most notable health benefits of “Shi Hu” are the effects of supplementing the stomach, nourishing the lungs to arrest cough, promoting the production of body fluids, clearing heat, enhancing the body’s immunity, anti-aging, and reducing blood sugar levels [3,4]. The chemical components of “Shi Hu” have been widely researched. Diverse metabolites such as alkaloids, bibenzyls, phenanthrenes, sesquiterpenoids, flavonoids, fluorenones, coumarins, and polysaccharides have been isolated [5,6,7]. The chemical profile and quality assessments of “Shi Hu” have also been studied with HPLC (high performance liquid chromatography) and hyphenated analytical techniques [8,9]. The biological activities of “Shi Hu” vary with effect due to the content of the active ingredients [10]. *D. nobile*, *D. officinale*, *D. loddigesii*, and *F. fimbriata* are four of the most frequently used “Shi Hu”. The dried stems of *D. loddigesii* or *F. fimbriata* are usually used in clinical medicine. The fresh or dried stems of *D. nobile* and *D. officinale* are regarded as valuable species, and used more as health products than herbal medicines such as *D. Officinale* teas, beverages, and broths [11,12]. *F. fimbriata* contains diterpenoids, polyphenols, flavonoids and glycosides, which has liver protective activity [13,14]. *D. officinale* has the highest content of polysaccharides in the *Dendrobium* species, which shows immunomodulatory, antioxidant, and hepatoprotective activities [15,16,17]. *D. nobile* contains alkaloids, bibenzyls, and phenanthrenes, which have anti-cataract and neuronal protective activities [15,18,19]. *D. loddigesii* contains bibenzyls and alkaloids, which shows the effects of reducing blood sugar levels and suppressing agglutination of platelets [15,20,21,22]. The existing research has mainly concentrated on the fat-soluble extract or polysaccharides; there have been few reports on their water-soluble metabolites. In China, the habits of eating “Shi Hu” are deconcocted with water such as formulations, broths, or the juices of fresh stems of *Dendrobium* [11,12], which should contain some water-soluble metabolites. Advanced analytical approaches of HPLC, LC-MS or LC-MS/MS have often been employed to characterize the complex composition of plants; but the pre-treatment of samples involves removing sugar by way of eluting with H_2_O on a C-18 SPE (solid-phase extract) cartridge [8,9]; in many cases, coexisting water-soluble metabolites would be removed. In order to discover more bioactive metabolites, the components and content of water-soluble metabolites in the H_2_O eluent from the four herbs were studied. In this work, we prepared the polar extracts from the H_2_O eluent using C-18 SPE. The isolation or analyses of the polar extracts could not be carried out using HPLC or LC-MS, which are usually equipped with a C-18 reverse phase column; therefore, the metabolites in the extracts were isolated using silica gel normal-phase chromatography as well as Sephadex LH-20 chromatography, and the contents of isolated metabolites were analyzed with qNMR. The various biological activities of the extracts and isolated metabolites were evaluated including anti-oxidation activity, α-glucosidase inhibitory activity, cytotoxicity, neuroprotective activity, and anti-inflammatory activity, which were chosen based on previous reports [4,13,14,18]. 

## 2. Results and Discussion

This work provided a new perspective to further understand the chemical composition of “Shi Hu”.

### 2.1. Water-Soluble Metabolite Structure

**Figure 1:**
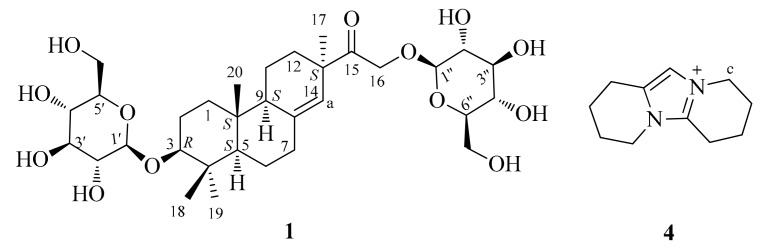
Structures of compounds **1** and **4** (H-a and H-c were the target protons for content determination).

In this work, the four polar extracts from *D. nobile*, *D. officinale*, *D. loddigesii*, and *F. fimbriata* were prepared; the metabolites in extracts were isolated and identified. Eight compounds (**1**–**8**) were obtained (Figure S1); their structures were elucidated based on spectroscopic analysis and comparison with the literature. One new pimarane-type diglucoside, flifimdioside A (**1**, Figure 1), along with two known glucosides, flickinflimoside B (**2**) [23] and syringaresinol-4′-*O*-d-glucopyranoside (**3**) [24], were isolated from the polar extract of *F. fimbriata* (PEF). A rare imidazolium-type alkaloid anosmine (**4**, Figure 1) was found from the polar extract of *D. nobile* (PEN) [25]. Three known compounds, malic acid (**5**) [26], 3-*O*-*β*-d-galactopyranosyl-*β*-d-galactopyranose (**6**) [27], and a mixture of fructose isomers (**7**) [28], were isolated from the polar extract of *D. officinale* (PEO). A known alkaloid, shihunine (**8**), was found from the polar extract of *D. loddigesii* (PEL) [20]. Compound **6** was also isolated from PEF.

**Figure 2:**
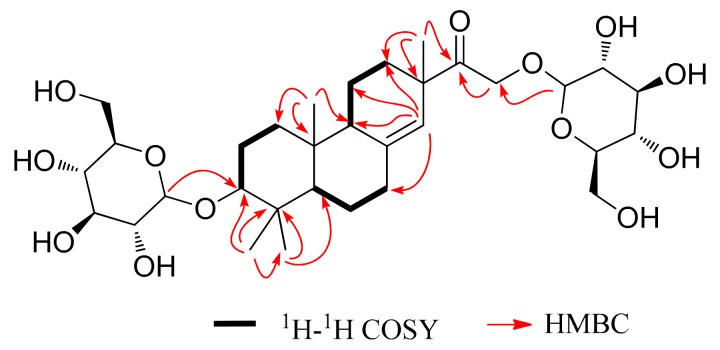
2D-NMR correlations of flifimdioside A (**1**).

Flifimdioside A (**1**, Figure 1) had a molecular formula of C_32_H_52_O_13_ based on its quasi molecular ion peak of *m*/*z* 643.33388 [M − H]^−^ (calcd. 643.33351) in HRESIMS (high resolution electrospray ionization mass spectrum). The NMR data of **1** are shown in Table 1 and Figure 2. Its ^13^C-NMR spectrum displayed the signals of one carbonyl group (*δ*c 213.7), two olefinic carbons, 11 oxygenic methylidyne groups, three oxygenic methylene groups, three sp^3^ quaternary carbons, two sp^3^ CH groups, six sp^3^ CH_2_ groups, and four methyl groups. The ^1^H-NMR spectrum of **1** showed signals matching the ^13^C-NMR data. In a comparison with the literature [13], the NMR data of **1**, from C-1 to C-20 (see Table 1), were similar to that of lonchophylloid B reported by Reference [13], which revealed that **1** had a structural fragment of lonchophylloid B; this finding was supported by the 2D-NMR of **1** (Figure 2). There were two *β*-glucopyranosyl moieties in **1**, which were derived from the comparison between the ^13^C chemical shifts of **1** (Table 1, from C-1′ to C-6″) and the data in Reference [29]. The chemical shifts of two anomeric carbons C-1′/C-1″ (*δ*_C_ 101.9/104.2) of glucopyranosyl moieties supported the *β*-configuration of the glycoside bonds [29]. d-configuration of the two glucopyranosyl groups was determined based on a comparison of the molecular rotations of **1** and flickinflimoside C, 16-*O*-*β*-d-glucopyranoside of lonchophylloid B [23], which was based on Kline’s rule [30]. The molecular rotation difference between the molecular rotation of **1** (−302.7°) and that of flickinflimoside C (−194.0°) was −108.7°, which was very close to the molecular rotation of methyl-*β*-d-glucopyranoside (−66.3°) [31]. On the basis of the above data, **1** was suggested as a lonchophylloid B diglucoside. Two *β*-d-glucosyl groups were indicated at C-3 and C-16 positions, respectively, which were supported by the HMBC (heteronuclear multiple-bond correlation) correlations between H-1′ and C-3 as well as H-1″ and C-16 (Figure 2). The absolute configuration of **1** was validated as (3*R*,5*S*,9*S*,10*S*,13*S*) configuration by comparing the ECD (electronic circular dichroism) spectra of **1** (Figure S8) and lonchophylloid B [13]; both showed a negative Cotton effect at 210 nm and a positive Cotton effect at 300 nm.

Anosmine (**4**, Figure 1) was an imidazolium-type alkaloid, which was the only imidazolium-type compound found in nature. Leander and Lüning isolated anosmine (**4**) from *D. anosmum* and *D. parishii* in 1968 [25]. Our research showed that *D. nobile* could produce a lot of anosmine (**4**). Compound **7** was a mixture of fructose isomers containing three isomers: *β*-pyranose, *β*-furanose, and *α*-furanose (51.8:28.5:19.7; see Figure S17) [28].

### 2.2. Content and Bioactivity

qNMR has been applied more frequently in natural product areas for its unique advantages: it does not need the references of the determined components, and it exhibits easy operation, non-destructiveness for the determined sample, high accuracy, and repeatability [32,33,34]. These advantages made it possible for us to evaluate the content of water-soluble metabolites using qNMR.

**Figure 3:**
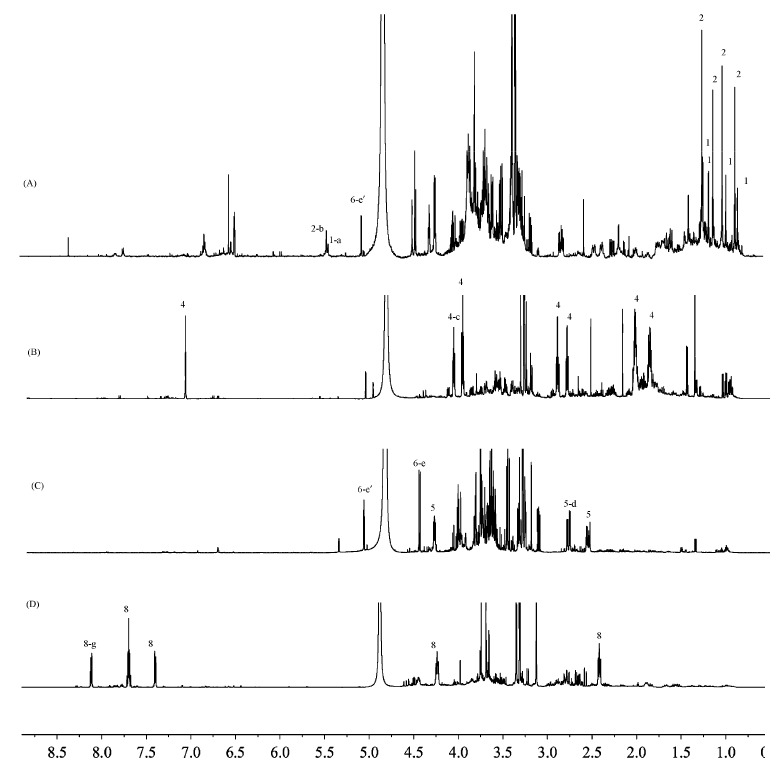
The ^1^H-NMR profiles of extracts. (**A**) *F. fimbriata*; (**B**) *D. nobile*; (**C**) *D. officinale*; and (**D**) *D. loddigesii*. The diagnostic signals of metabolites were labeled in the order number of metabolites; **1** = flifimdioside A; **2** = flickinflimoside B; **4** = anosmine; **5** = malic acid; **6** = 3-*O*-*β*-d-galactopyranosyl-*β*-d-galactopyranose; **8** = shihunine (Figure S1). The chemical shifts and splitting patterns of diagnostic signals are listed in Table S1.

The ^1^H-NMR profiles of the four polar extracts are shown in Figure 3. Several differences in the qualitative/quantitative composition of the four plant extracts were evident from the spectral analysis. Comparing the spectra of F. fimbriata (Figure 3A) and *D. officinale* (Figure 3C), similar signals of carbohydrates were observed in the spectral region from 3.0 to 5.5 ppm. *F. fimbriata* and *D. officinale* showed some metabolite similarities, with their molecules containing carbohydrate elements; the metabolites of F. fimbriata were glycosides, while the metabolites of *D. officinale* were fructose. Also, compound **6**, a disaccharide, was one common metabolite between F. fimbriata and *D. officinale*. Comparing Figure 3B,D, it seems that there were no similarities; the major signals of *D. nobile* were the signals of anosmine (**4**), and the major signals of *D. loddigesii* were those of shihunine (**8**). However, the similarity between *D. nobile* and *D. loddigesii* may be that both of them contain a large number of water-soluble alkaloids.

Figure 3 displays several diagnostic signals for each water-soluble metabolite, except for **3** and **7**. The signals of protons from 1-a to 8-g were chosen as the target peaks for content determination, since they were quite well separated from the others (Figures S19‒S23) and were not exchangeable protons [33]; another important reason was that the target peaks had smooth baselines in the ^1^H-NMR spectra, which could decrease the measuring error. The chemical shifts and splitting patterns of the target peaks are listed in Table 2. A solution of 0.18 M salicylic acid was used as the calibration for the electronic reference signal (Figure S24) [35]. The concentrations of water-soluble metabolites in the four crude herbs were determined by comparing the signal intensities of the target proton against the intensity of the reference signal of salicylic acid using ^1^H-NMR, and the results are shown in Table 2.

The content of **3** could not be evaluated as it was too low to show a suitable signal-to-noise ratio in the ^1^H-NMR spectrum of PEF. Compound **7** could not be evaluated by ^1^H-qNMR as its proton signals could not be well resolved. In the ^13^C-NMR spectrum of PEO, the anomeric carbon signals of three fructose isomers were well resolved (Figure S22).

The result of the quantitative analysis showed that the content of water-soluble metabolites was high, approximately 0.10–0.38% of the weight of crude drugs. Compared with the literature [8,9,10,15,36], the analysis showed that the water-soluble metabolites may be the most abundant metabolites.

Table 3 shows the results of the biological activities evaluation of the polar extracts as well as the water-soluble metabolites including DPPH (1,1-diphenyl-2-picryl-hydrazyl) radical scavenging activity, OH radical scavenging activity, inhibitory activity against α-glucosidase, and inhibitory activity against NO production.

**Figure 4:**
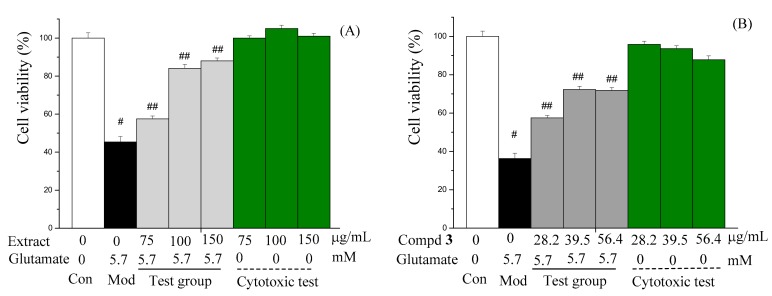
Effects of polar extract of *F. fimbriata* (**A**) and compound **3** (**B**) restraining glutamate-induced HT22 cell death. Data are presented as means ± standard deviation (*n* = 5); ^#^ *p* < 0.05 vs. the control (Con) group; ^##^ *p* < 0.05 vs. model (Mod) group.

The result showed that the four polar extracts and their main components had different biological activities. Three polar extracts had DPPH radical and OH radical scavenging effects, except for that of *D. nobile*. Compounds **1**, **4** and **8** had no antioxidant activity. PEO and PEF had moderate α-glucosidase inhibitory activity; compounds **1**–**4** and **8** had weak α-glucosidase inhibitory activity. Compounds **4** and **8** exhibited weak inhibitory activity against NO production in LPS (lipopolysaccharide)-activated RAW264.7 cells. The protective effect of the extracts and isolated metabolites against glutamate-induced HT22 cell apoptosis was evaluated. PEF and **3** showed good neuroprotection in a concentration-dependent manner (Figure 4A,B); their EC_50_ were 78.5 and 39.5 μg/mL, respectively. All samples had no cytotoxic activity against HeLa cells, HepG2 cells, MDA-MB- 231 human breast cancer cells, HT22 cells, and RAW 264.7 cells.

## 3. Materials and Methods

### 3.1. General Experimental Procedures

A Bellingham-Stanley 37–440 polarimeter (Bellingham Stanley Ltd., Kent, UK), a Chirascan Circular Dichroism Spectrometer (Applied Photophysics, Surrey, UK), a UV-240 spectrophotometer (Shimadzu, Tokyo, Japan), and a TENSOR37 spectrometer (Bruker Optics, Ettlingen, German) were used to determine the optical properties of the compounds. A multi-mode microplate reader FlexStation 3 (Molecular devices, Sunnyvale, California, USA) was used for bioactivity testing. ^1^H-NMR and ^13^C-NMR data were acquired using a Bruker Avance 400 spectrometer at 400 MHz for ^1^H nuclei and 100 MHz for ^13^C nuclei (Bruker Biospin, Rheinstetten, German). Content evaluations were carried out using a Bruker Avance 600 spectrometer (Bruker Biospin, Rheinstetten, German). NMR sample tubes were 5 mm Norell 509UP. The ESI (electrospray ionization) mass spectra and HRESIMS were obtained using an LTQ-Orbitrap LC-MS (Thermo Fisher, Frankfurt, German). SPE were performed using chromatographic columns of RP-18 gel (25–40 μm, Daiso Inc., Osaka, Japan). Isolations were carried out using chromatographic columns of silica gel (200–400 mesh, Qingdao Marine Chemical Inc., Qingdao, China) and Sephadex LH-20 (GE Healthcare, Stockholm, Sweden). 

Solvents were of analytical grade (Guangzhou Chemical Reagent Factory, Guangzhou, China); methanol-*d*_4_ (99.9%) was purchased from Aldrich (Milwaukee, WI, USA). Analytical-grade salicylic acid was purchased from Nanjing Reagent (Nanjing, China) and purified by recrystallization with ethanol-H_2_O. H_2_O_2_, 2,6-ditertbutyl-4-methyl-phenol (BHT), DPPH, FeSO_4_, and 1,10-phenanthroline were of analytical grade and bought from Aladdin (Shanghai, China). The following reagents were purchased from Sigma (St. Louis, MO, USA): l-glutamic acid hydrochloride, *p*-nitrophenyl-α-d-glucopyranoside, α-glucosidase from saccharomyces cerevisiae, *trans*-resveratrol, vitamin C (VC), *N*-1-naphthyletylenediamide-dihydrochloride, L-NG-monomethyl arginine citrate (L-NMMA), *N*-acetyl cysteine (NAC), *Escherichia coli* LPS, and paclitaxel.

### 3.2. Plant Sample

The plant samples were obtained from different origins. The dried stems of *D. loddigesii* and *F. fimbriata* (from Yunnan Province, China) were purchased in September 2015 from Caizilin Pharmacy in Guangzhou, China. *D. nobile* was bought in December 2015 from South China Botanical Garden (Guangzhou, China), dried in the shade, and stored at 4 °C until use. The fresh stems of *D. officinale* were bought in October 2016 from Yunnan Herba Dendrobii Biotechnology Development Co., Ltd. (Xishuangbanna, China), and stored at 4 °C until use (for about 6 weeks). The four plant samples were identified with the classical method by pharmaceutical botanist Prof. Lin Jiang, School of Pharmaceutical Sciences, Sun Yat-Sen University. The voucher specimens (No. 20150913l, 20150913f, 20151215n, and 20161025o) were deposited in the School of Pharmaceutical Sciences, Sun Yat-Sen University (Guangzhou, China).

### 3.3. Extraction

The dried stems of *F. fimbriata* (50 g), dried stems of *D. nobile* (17.2 g), fresh stems of *D. officinale* (200 g), and dried stems of *D. loddigesii* (30 g) were used in the study. The extraction of each sample was performed as follows. Plant sample was ground, and then extracted with a mixture of acetone-water (80:20, *v*/*v*) using ultrasonication for 80 min at room temperature. The extraction was repeated three times. The extract was filtered, combined, and evaporated under vacuum to generate the crude extract. Four crude extracts, namely, crude-extract-f (4.30 g) from *F. fimbriata*, crude-extract-n (1.88 g) from *D*. *nobile*, crude-extract-o (4.95 g) from *D. officinale*, and crude-extract-l (2.77 g) from *D. loddigesii*, were obtained.

### 3.4. Polar Extract Preparation

The polar extract was enriched from the corresponding crude extract using C-18 SPE, which was performed as follows. The crude extract obtained was dissolved with methanol (about 3 mL) and deposited on a RP-18 column (130 mm × 20 mm Φ). The column that adsorbed the sample was eluted with five bed volumes of H_2_O (20% MeOH-H_2_O for *F. fimbriata*). The H_2_O eluate was collected and evaporated to generate the polar extract. Four polar extracts, polar-extracts-f (1.80 g) from crude-extract-f, polar-extracts-n (0.78 g) from crude-extract-n, polar-extracts-o (3.65 g) from crude-extract-o, and polar-extracts-l (0.95 g) from crude-extract-l, were obtained. 

### 3.5. qNMR Analysis

Samples for qNMR analysis were prepared as follows: 30 mg dried polar extract was weighed out precisely, re-dissolved in 1 mL of methanol-*d*_4_, and the container of the sample was sealed. Subsequently, the compounds were dissolved using vortex-shaking and centrifuged for 3 min (3000 r/min) to remove the suspended matter. A 600-μL aliquot of the supernatant solution was transferred into an NMR tube and analyzed immediately by ^1^H-NMR. The same operation was performed for salicylic acid, which was used as an external standard. All experiments were performed in triplicate.

^1^H-NMR spectra were recorded on a Bruker Avance 600 NMR spectrometer. Topspin 3.5 software (Bruker, Germany) was used. The ^1^H-NMR quantitative experimental parameters were set based on those described by Tapiolas et al. [32]. Quantification was performed using the magnetic resonance quantitative tool-ERETIC2 (Electronic Reference To access In vivo Concentrations) based on Topspin 3.5 software (Bruker Biospin, Rheinstetten, German) [35].

### 3.6. Water-Soluble Metabolite Isolation

Water-soluble metabolites were isolated from each of the polar extracts. Isolation was conducted as follows. The polar extract was isolated on a silica gel column by direct phase elution modes (CH_2_Cl_2_-MeOH gradient from 20:1 to 50:50, *v*/*v*). The fractions collected were purified repeatedly by the Sephadex LH-20 column (MeOH) to obtain pure compounds. Finally, eight compounds, **1** (50.3 mg), **2** (40.5 mg), **3** (3.2 mg), **4** (133.2 mg), **5** (426.1 mg), **6** (101.1 mg and 28.8 mg), **7** (166 mg), and **8** (110.5 mg), were obtained. 

### 3.7. Spectral Data

Flifimdioside A (**1**): white solid; soluble in MeOH, but insoluble in MeCN; [α]${}_{D}^{20}$ −47 (*c* 0.074, MeOH); UV (MeOH) λ_max_ (log*ε*): 203 (1.42) nm; ECD (MeOH) λ_max_ (Δ*ε*): 206 (−6.03), 300 (+0.39) nm; IR (KBr) ν_max_: 3352, 2936, 1709, 1363, 1074 cm^−1^; for ^1^H and ^13^C-NMR data see Table 1 and Figure 2; HRESIMS *m*/*z*: 643.33388 [M − H]^−^ (calcd for C_32_H_51_O_13_, 643.33351).

### 3.8. Biological Assay

The biological activities of the four polar extracts and isolated water-soluble metabolites were evaluated using the following protocol. The methods of DPPH radical scavenging activity and OH radical scavenging activity were used to evaluate the antioxidant activities [37,38,39]. BHT and VC were used as positive controls. Evaluation of the α-glucosidase inhibitory activity was carried out according to the method in the literature [40]. *Trans*-resveratrol was used as a positive control. The anti-inflammatory activity was investigated using the method of inhibition of nitric oxide (NO) production in RAW 264.7 cells activated by LPS, which were operated as per the literature [41,42]. L-NMMA was used as a positive control. The protective effect against glutamate-induced HT22 cell death was evaluated according to the literature [43]. NAC was used as a positive control. Cytotoxic bioassay was performed using the MTT assay as per the literature [44]. Paclitaxel was used as a positive control. Five cell lines, HeLa cells, HepG2 cells, MDA-MB-231 human breast cancer cells, HT22 cells, and RAW 264.7 cells, were used for the cytotoxic evaluation of all samples. 

## Figures and Tables

**Table 1:** ^1^H-NMR (400 MHz) and ^13^C-NMR (100 MHz) data of flifimdioside A (**1**) (in CD_3_OD, *J* in Hz).

Position	*δ*_C_	*δ*_H_	Position	*δ*_C_	*δ*_H_	Position	*δ*_C_	*δ*_H_
1	37.7 t	1.68 m, 1.16 m	11	23.2 t	1.61 m, 1.46 m	1′	101.9 d	4.33 d (7.6)
2	24.1 t	1.79 m, 1.55 m	12	33.6 t	2.29 m, 1.14 m	2′	75.1 d	3.26–3.18 m
3	85.8 d	3.38 m	13	48.6 s		3′	78.2 d	3.37–3.27 m
4	39.4 s		14	124.8 d	5.52 s	4′	71.9 d	3.29 m
5	55.9 d	1.15 m	15	213.7 s		5′	77.6 d	3.37–3.27 m
6	21.2 t	1.63 m, 1.23 m	16	72.4 t	4.86 d (18.4)	6′	62.9 t	3.86 m, 3.67 m
7	36.7 t	2.04 m, 2.11 m			4.50 d (18.4)	1″	104.2 d	4.25 d (7.6)
8	143.5 s		17	27.5 q	1.13 s	2″	74.9 d	3.26–3.18 m
9	51.9 d	1.78 m	18	17.3 q	0.87 s	3″	78.1 d	3.37–3.27 m
10	40.4 s		19	29.1 q	1.06 s	4″	71.4 d	3.29 m
			20	15.1 q	0.73 s	5″	77.7 d	3.37–3.27 m
						6″	62.7 t	3.86 m, 3.67 m

**Table 2:** The concentrations of water-soluble metabolites in the four crude herbs by qNMR ^a^.

Plant	Compound	Target Signal	Concentration (mg/g)
		δ (ppm, Multiplicity Hz)	
*F. fimbriata*	flifimdioside A (**1**)	H-a/5.51 (s, 1H)	0.97
	flickinflimoside B (**2**)	H-b/5.54 (s, 1H)	1.63
	syringaresinol-4′-*O*-d-glucopyranoside (**3**)	/	/
	3-*O*-*β*-d-galactopyranosyl-*β*-d-galactopyranose (**6**)	H-e′/5.12 (d, 2.4, 1H)	0.96
*D. nobile*	anosmine (**4**)	H-c/4.11 (t, 3.6, 2H)	3.79
*D. officinale*	malic acid (**5**)	H-d/2.78 (dd, 10.8, 3.6, 1H)	1.38
	3-*O*-*β*-d-galactopyranosyl-*β*-d-galactopyranose (**6**)	H-e/4.48 (d, 5.2, 1H)	2.42
	*β*-pyranose (**7**)	C-f/99.2 (s)	about 1.09
	*β*-furanose (**7**)	C-f′/103.1 (s)	about 0.32
	*α*-furanose (**7**)	C-f″/105.3 (s)	about 0.069
*D. loddigesii*	shihunine (**8**)	H-g/8.11 (dd, 4.4, 1.2, 1H)	2.27

^a^ Recorded on mg/g of crude drug; *n* = 3.

**Table 3:** Biological activities of extracts and isolated metabolites expressed in IC_50_ μg/mL.

		DPPH	OH	α-glucosidase	NO
**Sample**	polar extract of *F. fimbriata*	131.8	274.7	396.7	a.a ^a^
	**1**	a.a	a.a	40.3	a.a
	**2**	a.a	a.a	45.8	a.a
	**3**	48.1	a.a	40.4	a.a
	polar extract of *D. nobile*	1278.9	a.a	396.7	122
	**4**	a.a	a.a	17.7	16.1
	polar extract of *D. officinale*	1032.0	457.5	161.7	a.a
	polar extract of *D. loddigesii*	317	486.3	682.2	130
	**8**	a.a	a.a	21.9	11.5
**Positive control**	*trans*-resveratrol	n.t ^b^	n.t	7.7	n.t
	L-NG-monomethyl arginine citrate	n.t	n.t	n.t	7.2
	2,6-ditertbutyl-4-methyl-phenol	5.7	n.t	n.t	n.t
	vitamin C	n.t	6.3	n.t	n.t

^a^ a.a: absence of activity; ^b^ n.t: not tested. All samples for bioactivity evaluation were prepared in DMSO.

## Supplementary Materials

The supplementary materials are available online. Figure S1. Structures of **1**–**8** and their target protons (a−g) for content determination; Figures S2. The NMR spectra, ECD spectrum, and HRMS spectrometry for flifimdioside A (**1**); Figures S9. The ^1^H-NMR spectra or ^13^C-NMR spectrum for compounds **2**–**8**; Figures S19. The NMR spectra for quantitative analysis; Figures S25 and S26. The inhibitory activities of polar extracts or isolated metabolites on α-glucosidase.
