# Supplementary material for: Study on the Polar Extracts of Dendrobium nobile, D. officinale, D. loddigesii, and Flickingeria fimbriata: Metabolite Identification, Content Evaluation, and Bioactivity Assay
Source: Molecules. 2018 May 15;23(5):1185. doi: 10.3390/molecules23051185 (PMC6099805; doi:10.3390/molecules23051185)
Supplement: Supplementary file 1 [file molecules-23-01185-s001.zip › molecules-287670-supplementary/Supplementary Materials/figures and table in Supplementary Materials/Table S1.docx]

**Table S1.**The chemical shifts and splitting patterns of diagnostic signals of isolated metabolites

| Plant | Compound | Diagnostic signals | |
| --- | --- | --- | --- |
|  |  | δ (ppm, J, Hz) | |
| *F. fimbriata* | flifimdioside A (1) | 5.51 (s, 1H-a), 1.13 (s, 3H), 1.06 (s, 3H), 0.86 (s, 3H), 0.72 (s, 3H). | |
|  | flickinflimoside B (2) | 5.54 (s, 1H-b), 1.14 (s, 3H), 1.01 (s, 3H), 0.90 (s, 3H), 0.76 (s, 3H). | |
|  | syringaresinol-4′-*O*-D-glucopyranoside (3) | / | |
|  | 3-*O*-*β*-D-galactopyranosyl-*β*-D-galactopyranose (6) | 5.12 (d, 2.4, 1H-e'). | |
| *D. nobile* | anosmine (4) | 7.14 (s, 1H), 4.11 (t, 3.6, 2H-c), 4.01 (t, 4.4, 2H), 2.94 (t, 4.4, 2H), 2.83 (t, 4.4, 2H), 2.07 (m 4H), 1.89 (m 4H) | |
| *D.officinale* | malic acid (5) | 4.31 (m 1H), 2.78 (dd, 10.8, 3.6, 2H-d), 2.57 (dd, 10.8, 4.8) | |
|  | 3-*O*-*β*-D-galactopyranosyl-β-D-galactopyranose (6) | 5.11 (d, 2.4, 1H-e'), 4.48 (d, 5.2, 1H-e) | |
|  | *β*-pyranose (7) | C-f/99.2 (s) |  |
|  | *β*-furanose (7) | C-f′/103.1 (s) |  |
|  | *α*- furanose (7) | C-f″/105.3 (s) |  |
| *D. loddigesii* | shihunine (8) | 8.11 (dd, 4.4, 1.2, 1H-g/), 7.69 (m, 2H), 7.39 (dd, 4.4, 1.2, 1H), 4.23 (t, 5.2, 2H), 2.41 (dd, 5.2, 2H) | |
