# Supplementary material for: Study on the Polar Extracts of Dendrobium nobile, D. officinale, D. loddigesii, and Flickingeria fimbriata: Metabolite Identification, Content Evaluation, and Bioactivity Assay
Source: Molecules. 2018 May 15;23(5):1185. doi: 10.3390/molecules23051185 (PMC6099805; doi:10.3390/molecules23051185)
Supplement: Supplementary file 1 [file molecules-23-01185-s001.zip › molecules-287670-supplementary/Supplementary Materials/figures and table in Supplementary Materials/Supplementary Materials.docx]

Supplementary Materials

**Study on** **the polar extracts of** ***Dendrobium nobile*, *D. officinale*, *D. loddigesii* and** ***Flickingeria fimbriata*:** **metabolite identification****, content evaluation and** **bioactivity assay**

Huiping Chen^1,†^**,** Xuewen Li^1,†^**,** Yongli Xu^1^**,** Kakei Lo^1^**,** Huizhen Zheng^1^**,** Haiyan Hu^1,*^**,** Jun Wang^1*^ and Yongcheng Lin^2^

^1^ School of Pharmaceutical Sciences, Sun Yat-sen University, Guangzhou 510006, China

^2^ School of Chemistry and Chemical Engineering, Sun Yat-sen University, Guangzhou 510275, China

^*^ Corresponding author

E-mail address: [lsshhy@mail.sysu.edu.cn](mailto:lsshhy@mail.sysu.edu.cn) (H-Y. Hu), or, [wjun@mail.sysu.edu.cn](mailto:wjun@mail.sysu.edu.cn) (J. Wang)

† Those authors contribute equally to this work.

**Figure Legends:**

**Figure S1**. Structures of **1**−**8** and their target protons (a−g) for content determination

**Figure S2** ^1^H NMR for flifimdioside A (**1**) from *F. fimbriata*

**Figure S3** ^13^C NMR for flifimdioside A (**1**) from *F. fimbriata*

**Figure S4** ^1^H-^1^H COSY for flifimdioside A (**1**) from *F. fimbriata*

**Figure S5** HSQC for flifimdioside A (**1**) from *F. fimbriata*

**Figure S6** HMBC for flifimdioside A (**1**) from *F. fimbriata*

**Figure S7** HRMS spectrometry for flifimdioside A (**1**) from *F. fimbriata*

**Figure S8** ECD spectrum for flifimdioside A (**1**) from *F. fimbriata*

**Figure S9** ^1^H NMR for flickinflimoside B (**2**) from *F. fimbriata*

**Figure S10** ^1^H NMR for syringaresinol-4′-*O*-D-glucopyranoside (**3**) from *F. fimbriata*

**Figure S11** ^1^H NMR for anosmine (**4**) from *D. nobile*

**Figure S12** ^1^H NMR for malic acid (**5**) from *D.officinale*

**Figure S13** ^1^H NMR for compound (**6**) from *D.officinale*

**Figure S14** ^13^C-NMR for compound (**6**) from *D.officinale*

**Figure S15** ^13^C-NMR for compound (**6**) from *F. fimbriata*

**Figure S16** ^1^H NMR for mixture of three fructose isomer (**7**) from *D.officinale*

**Figure S17** ^13^C NMR the mixture of three fructose isomer (**7**) from *D.officinale*

**Figure S18** ^1^H NMR for shihunine (**8**) from *D. loddigesii*

**Figure S19** ^1^H NMR of polar-extract-f for content determination

**Figure S20** ^1^H NMR of polar-extract-n for content determination

**Figure S21** ^1^H NMR of polar-extract-o for content determination

**Figure S22** ^13^C NMR of polar-extract-o for content determination

**Figure S23** ^1^H NMR of polar-extract-l for content determination

**Figure S24** ^1^H NMR of salicylic acid for an external standard

All NMR data were recorded in CD_3_OD.

**Figure S25** The inhibitory activities of the polar extracts on α-glucosidase

**Figure S26** The inhibitory activities of isolated metabolites on α-glucosidase

**Table S1** The chemical shifts and splitting patterns of diagnostic signals of isolated metabolites

**Figure S1**. Structures of **1**−**8** and their target protons (a−g) for content determination


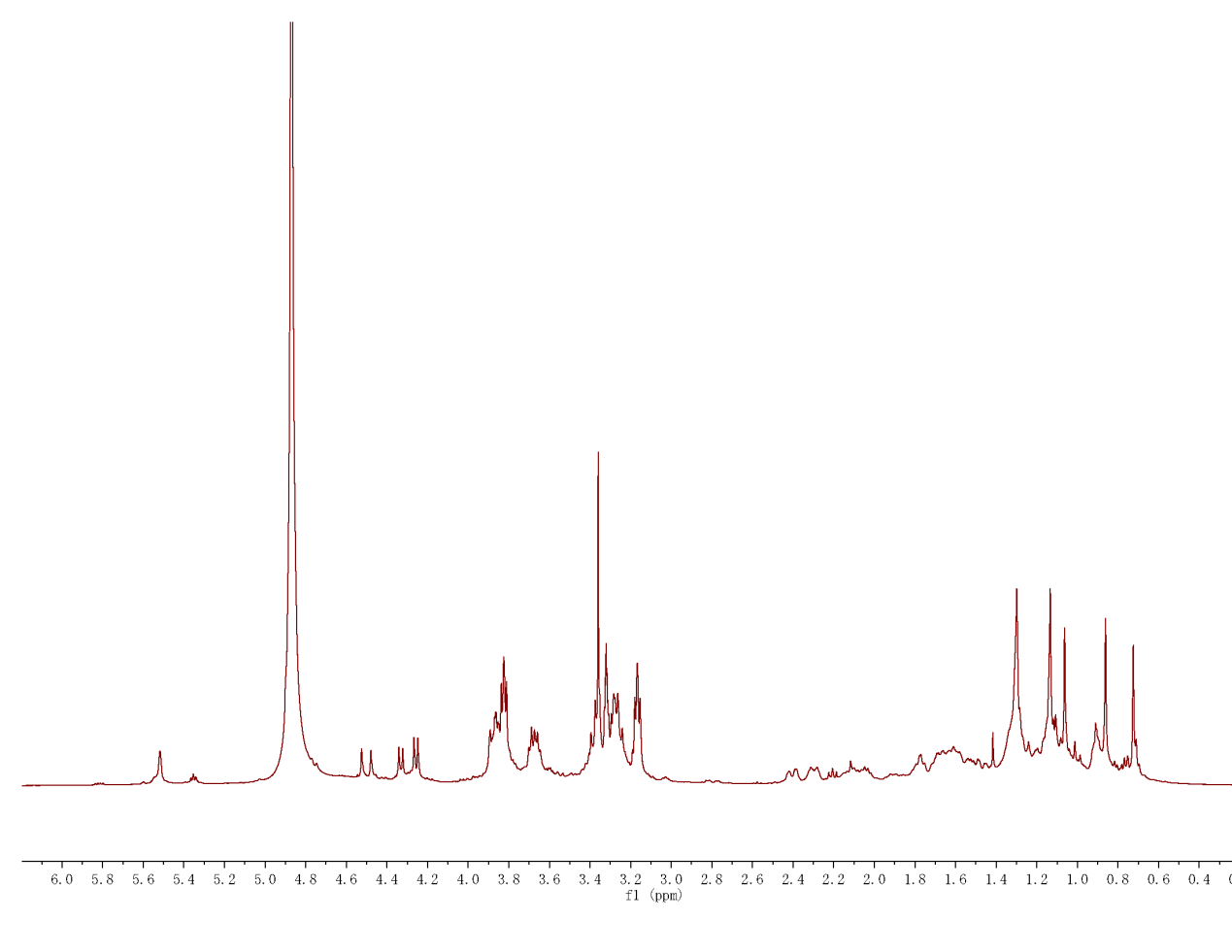


**Figure S2** ^1^H NMR for flifimdioside A (**1**) from *F. fimbriata*


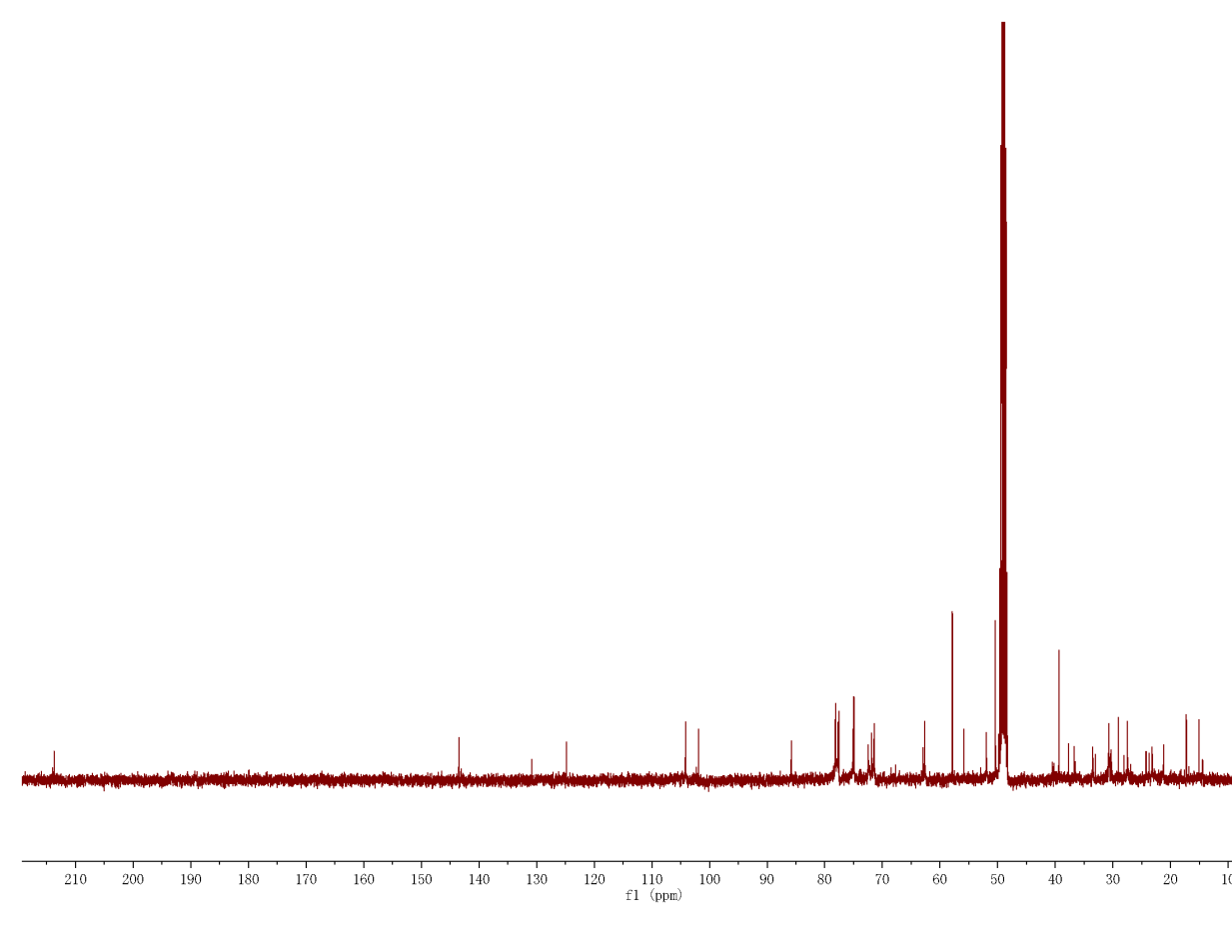


**Figure S3** ^13^C NMR for flifimdioside A (**1**) from *F. fimbriata*

**
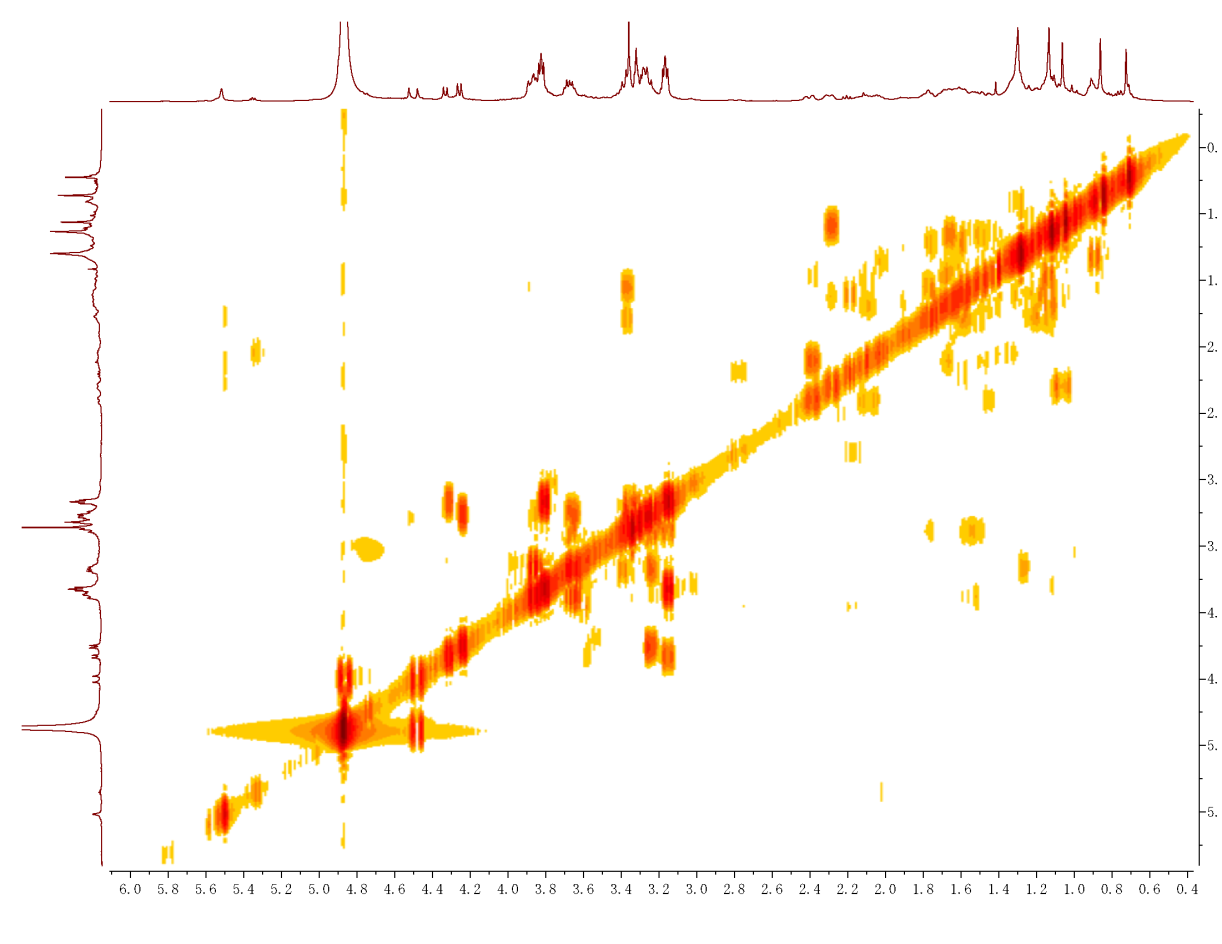
**

**Figure S4** ^1^H-^1^H COSY for flifimdioside A (**1**) from *F. fimbriata*


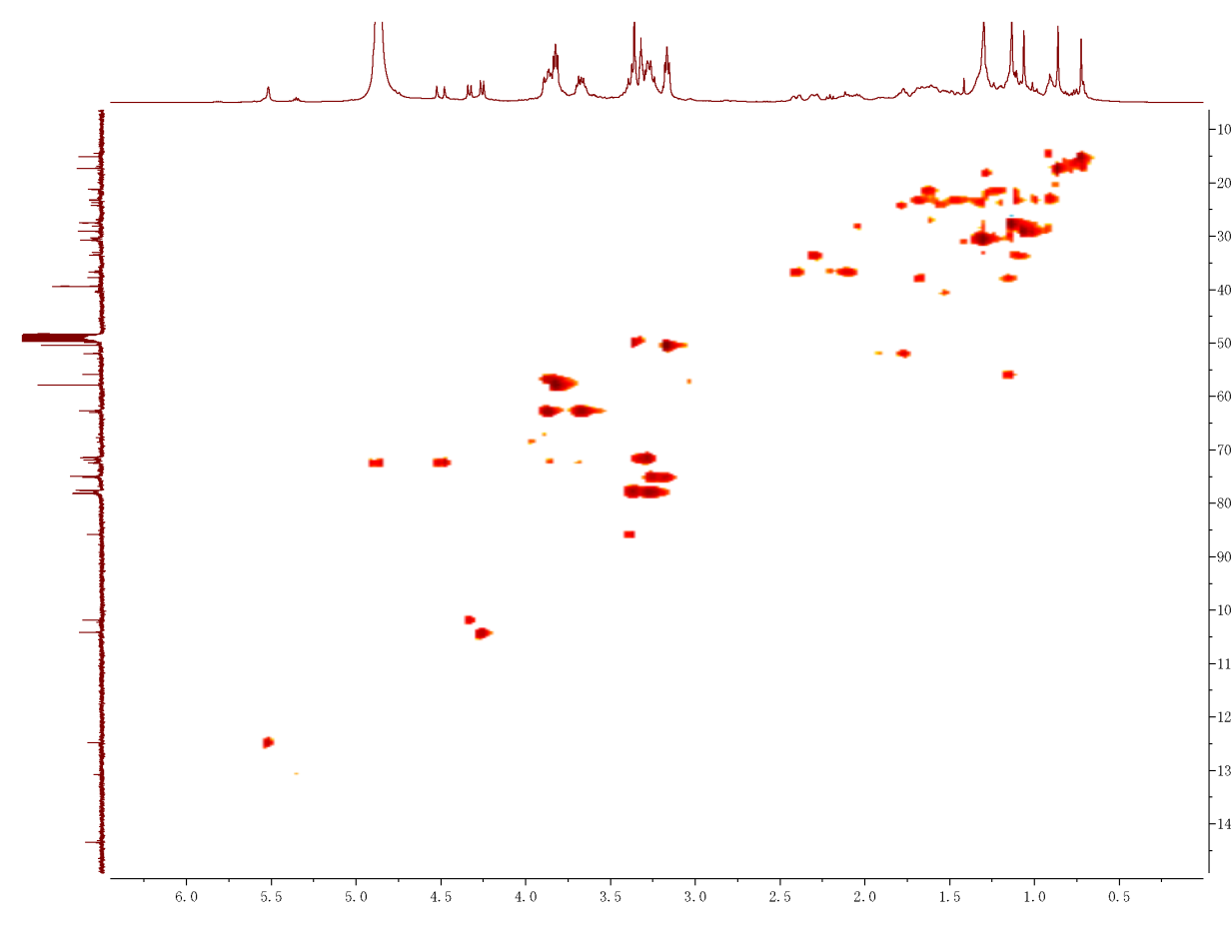


**Figure S5** HSQC for flifimdioside A (**1**) from *F. fimbriata*

**
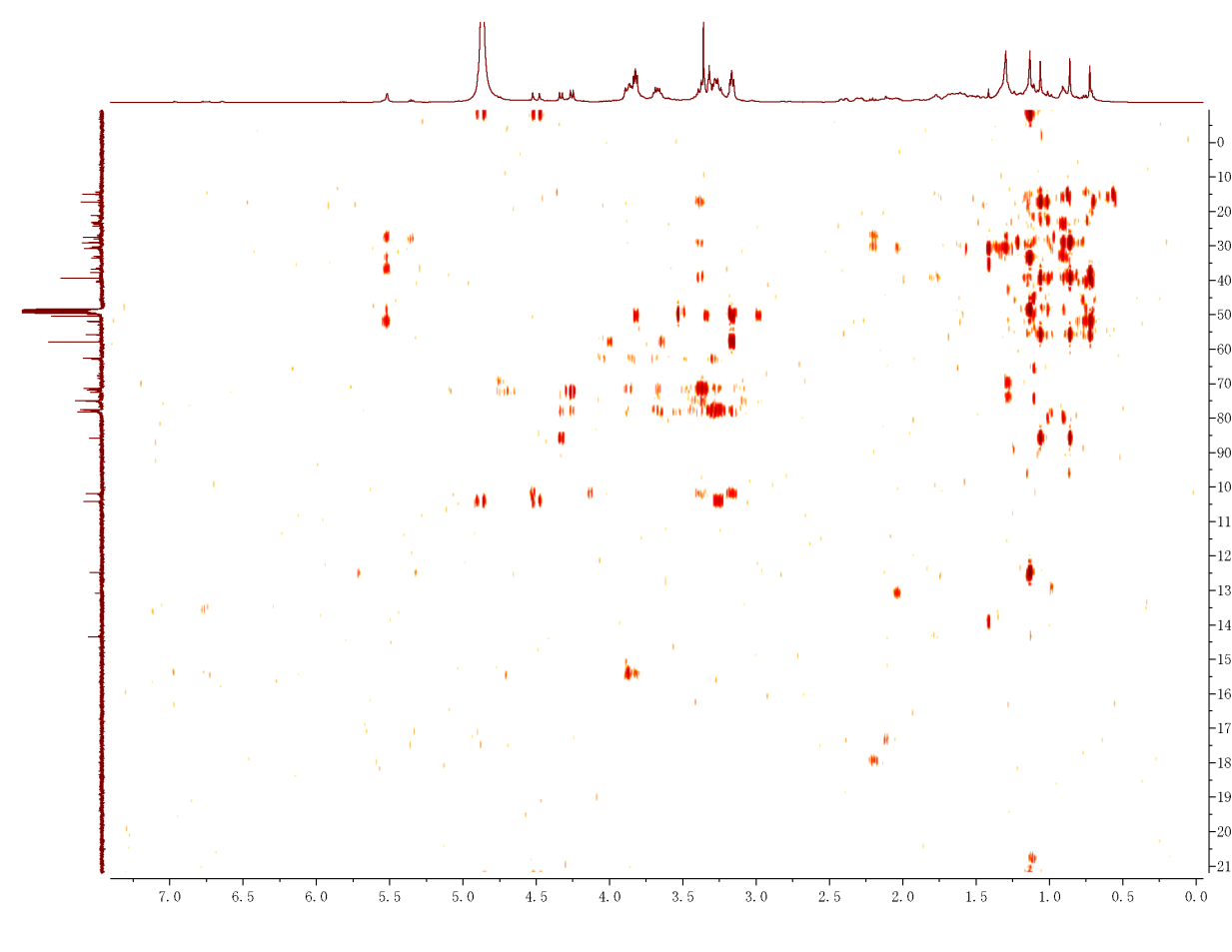
**

**Figure S6** HMBC for flifimdioside A (**1**) from *F. fimbriata*


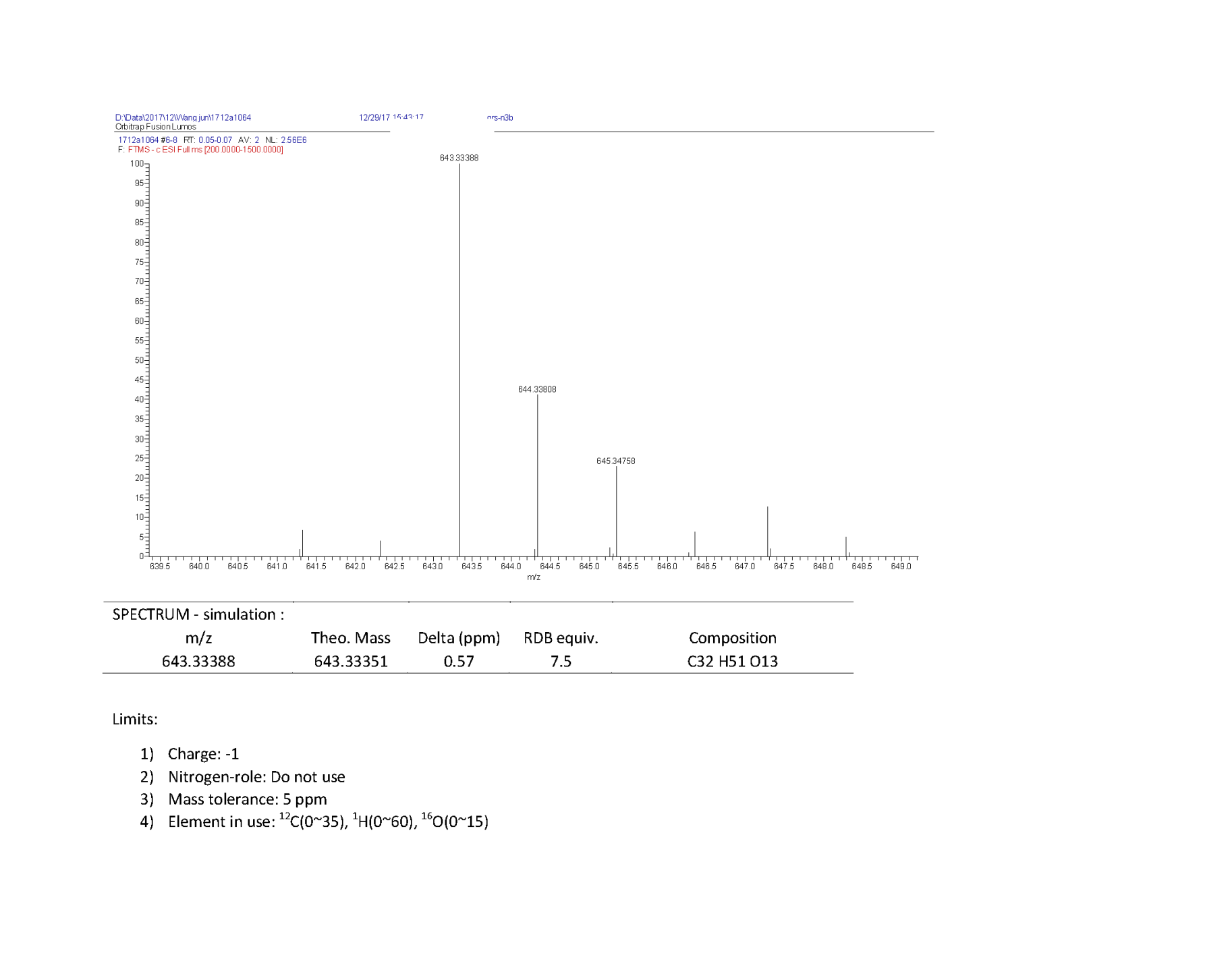


**Figure S7** HRMS spectrometry for flifimdioside A (**1**) from *F. fimbriata*


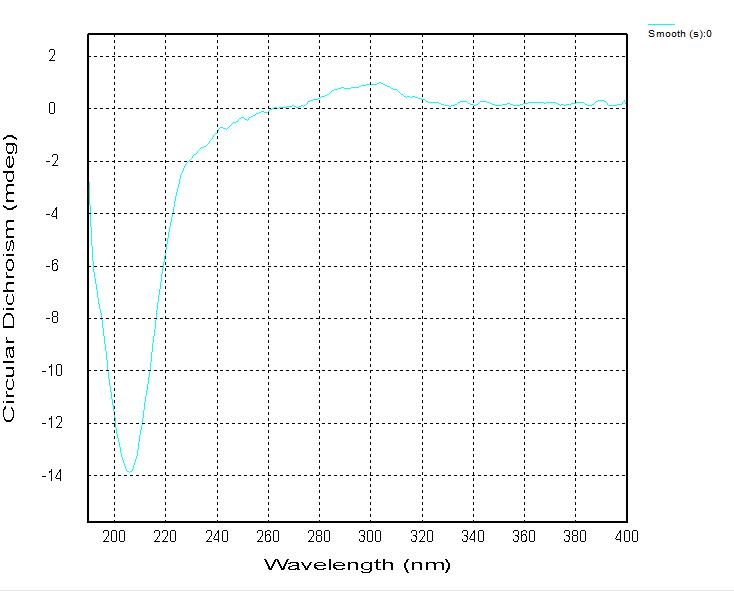


**Figure S8** ECD spectra for flifimdioside A (**1**) from *F. fimbriata*


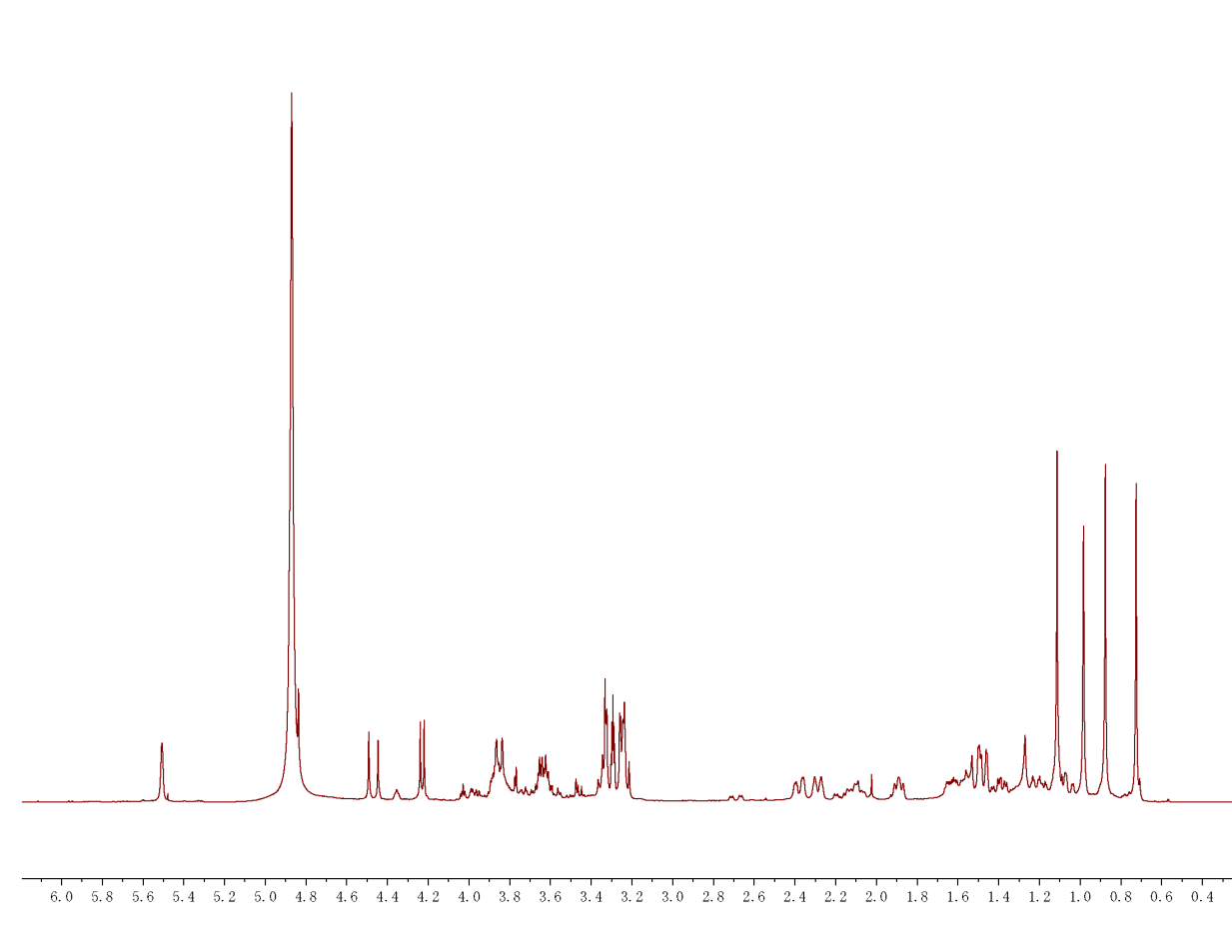


**Figure S9** ^1^H NMR for flickinflimoside B (**2**) from *F. fimbriata*


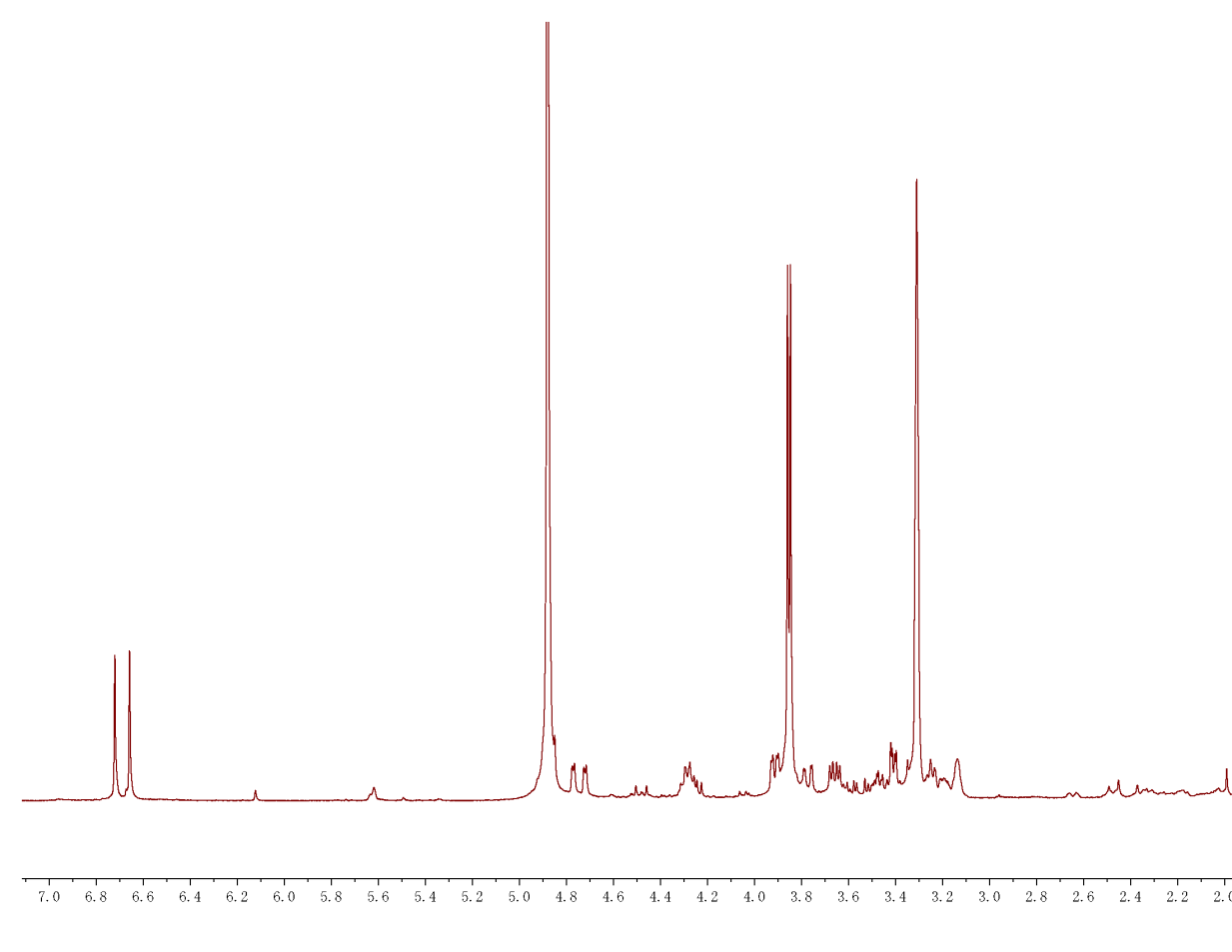


**Figure S10** ^1^H NMR for syringaresinol-4′-*O*-D-glucopyranoside (**3**) from *F. fimbriata*

**
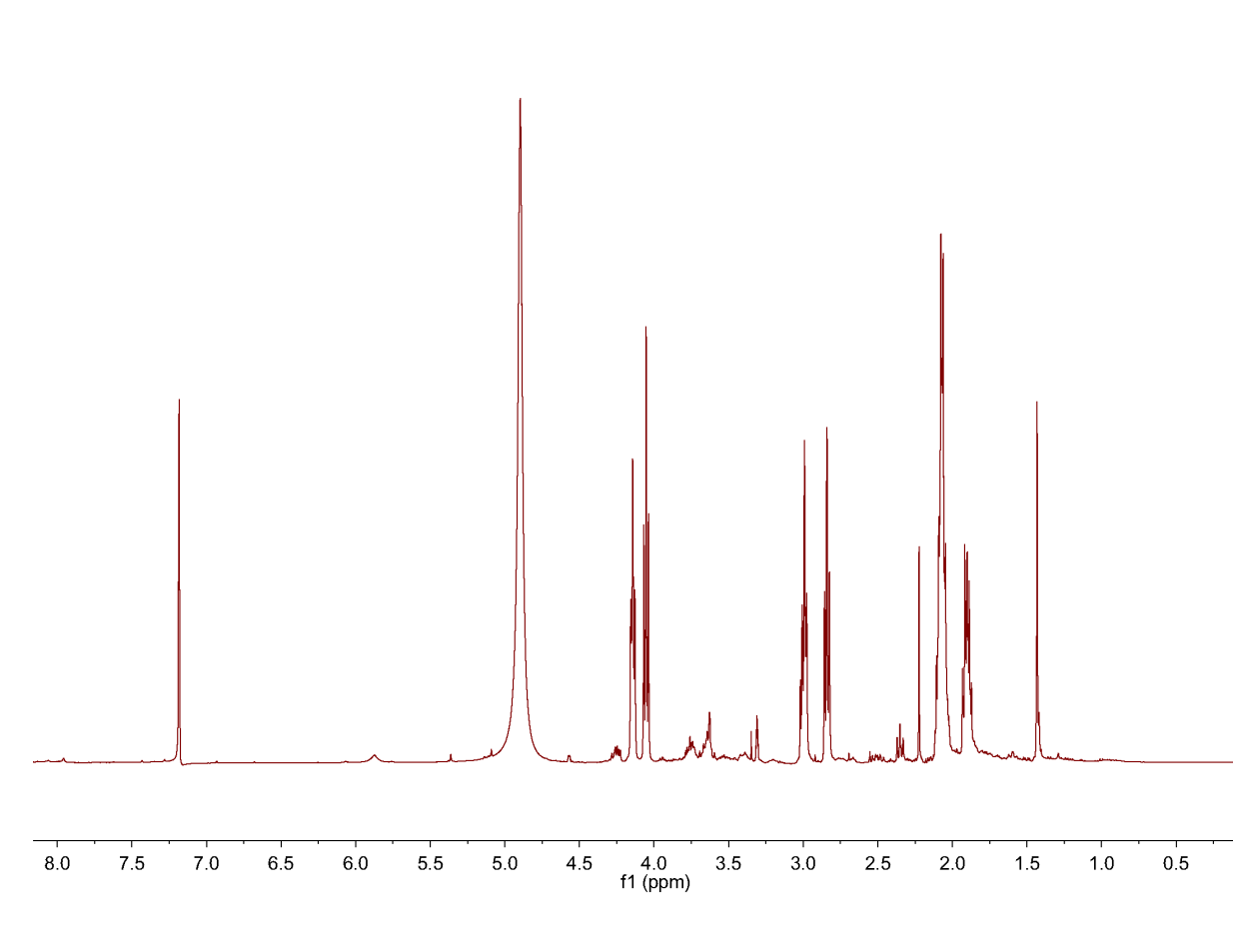
**

**Figure S11** ^1^H NMR for anosmine (**4**) from *D. nobile*

**
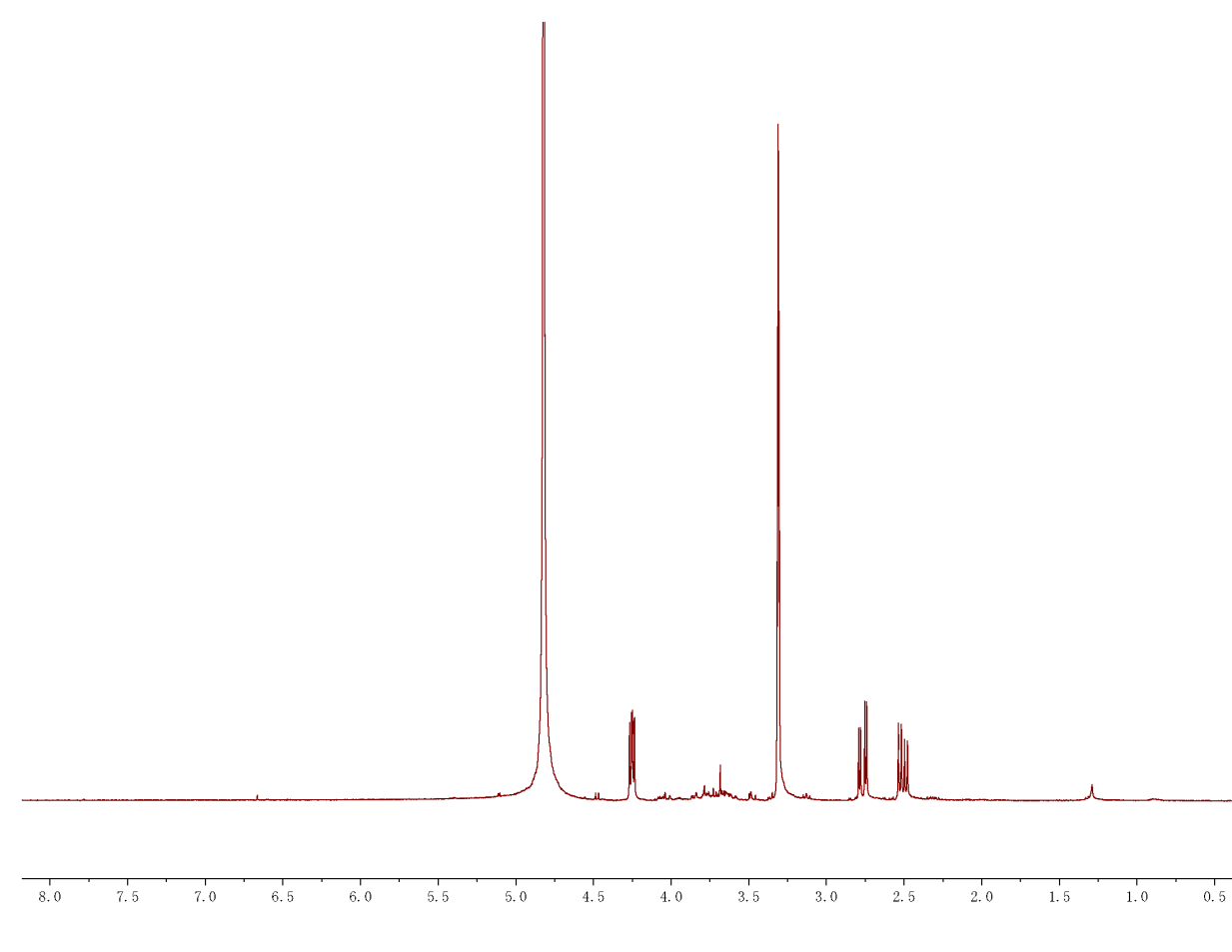
**

**Figure S12** ^1^H NMR for malic acid (**5**) from *D. officinale*


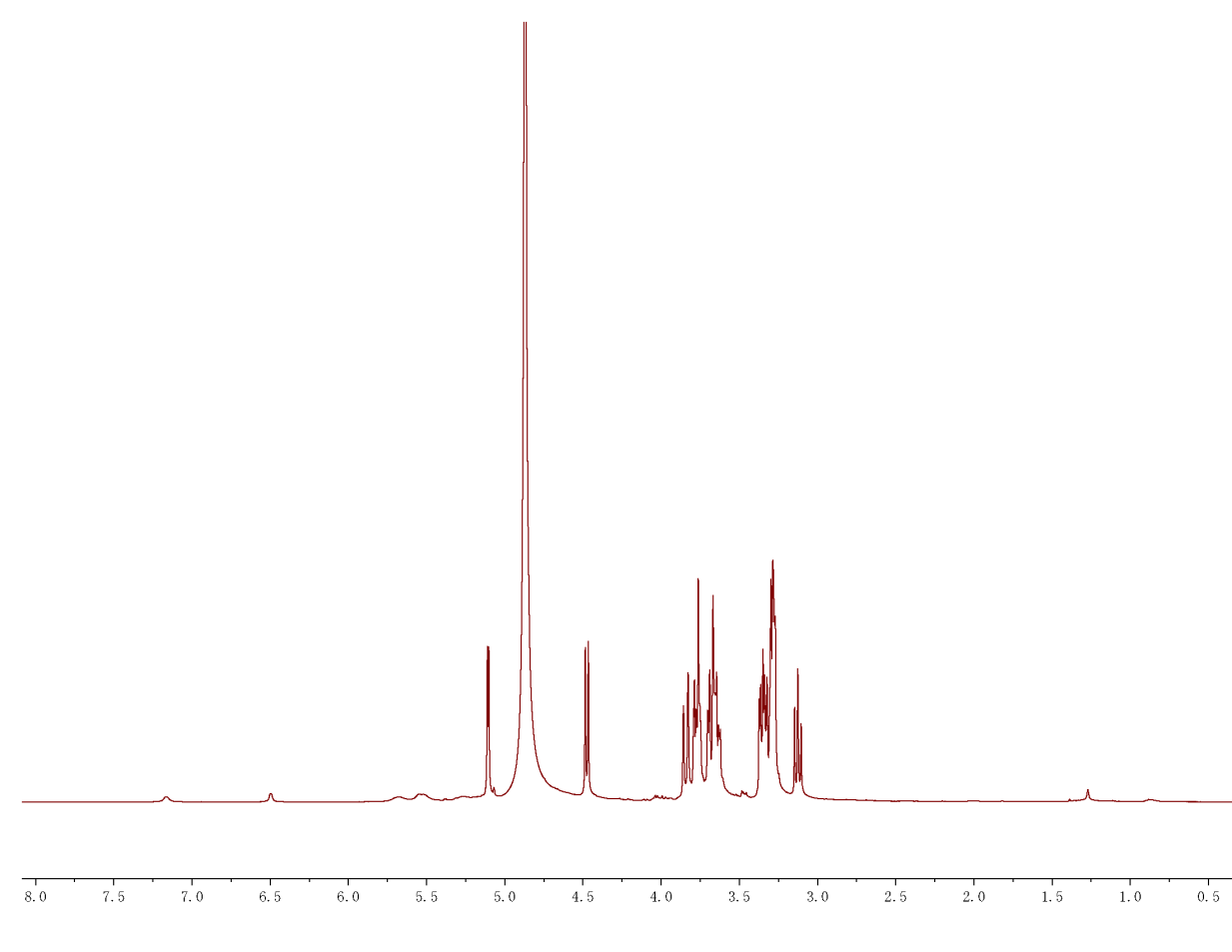


**Figure S13** ^1^H NMR for compound (**6**) from *D. officinale*


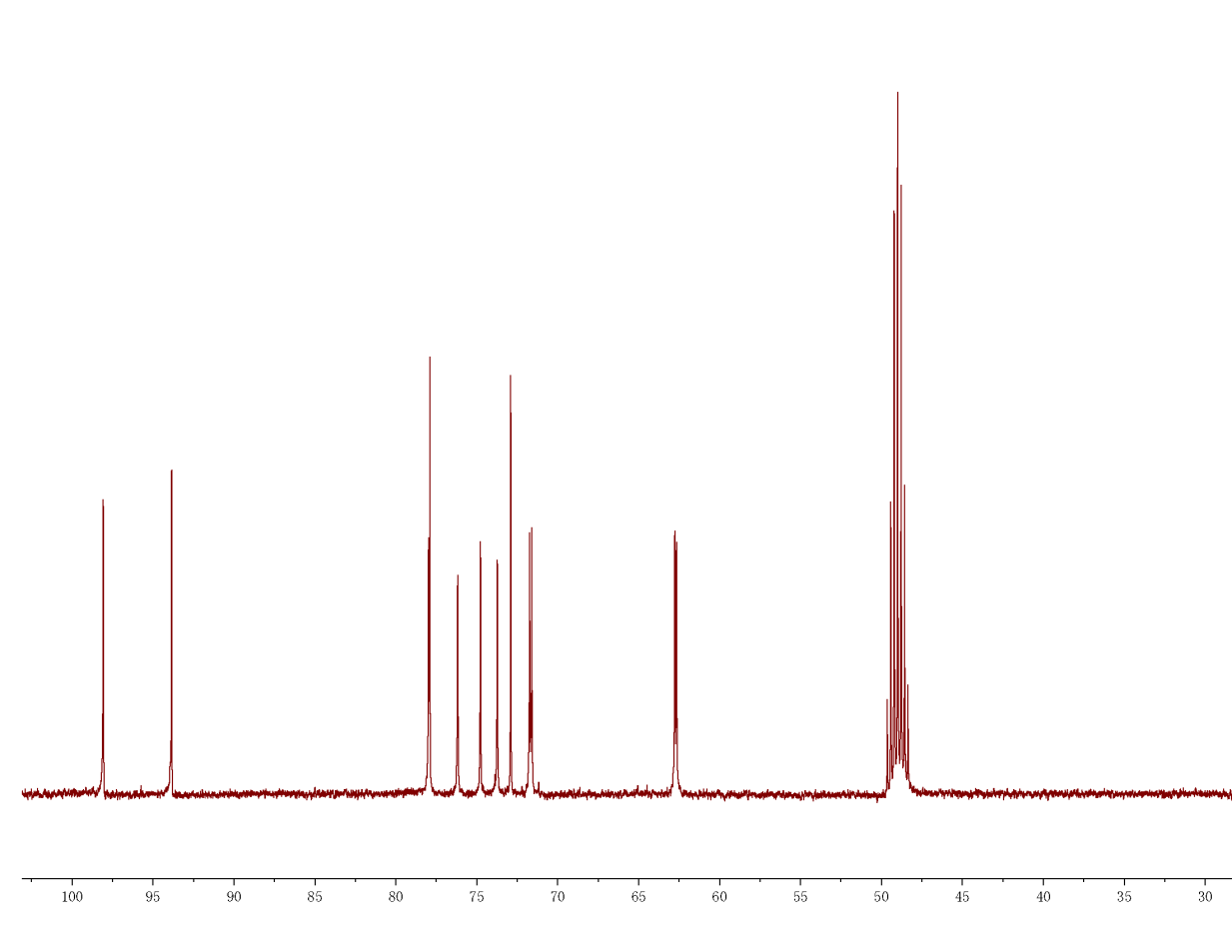


**Figure S14** ^13^C-NMR for compound (**6**) from *D. officinale*


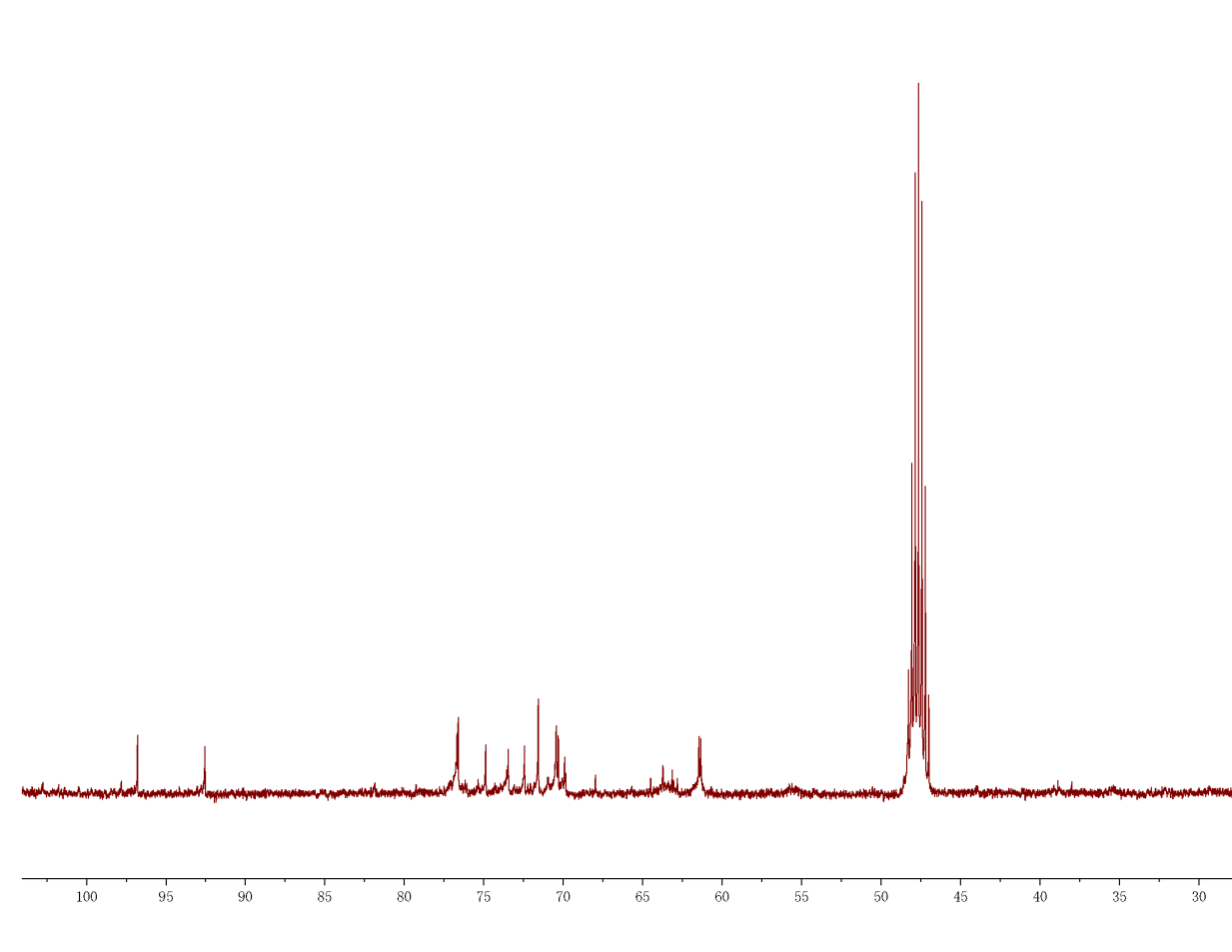


**Figure S15** ^13^C-NMR for compound (**6**) from *F. fimbriata*

**
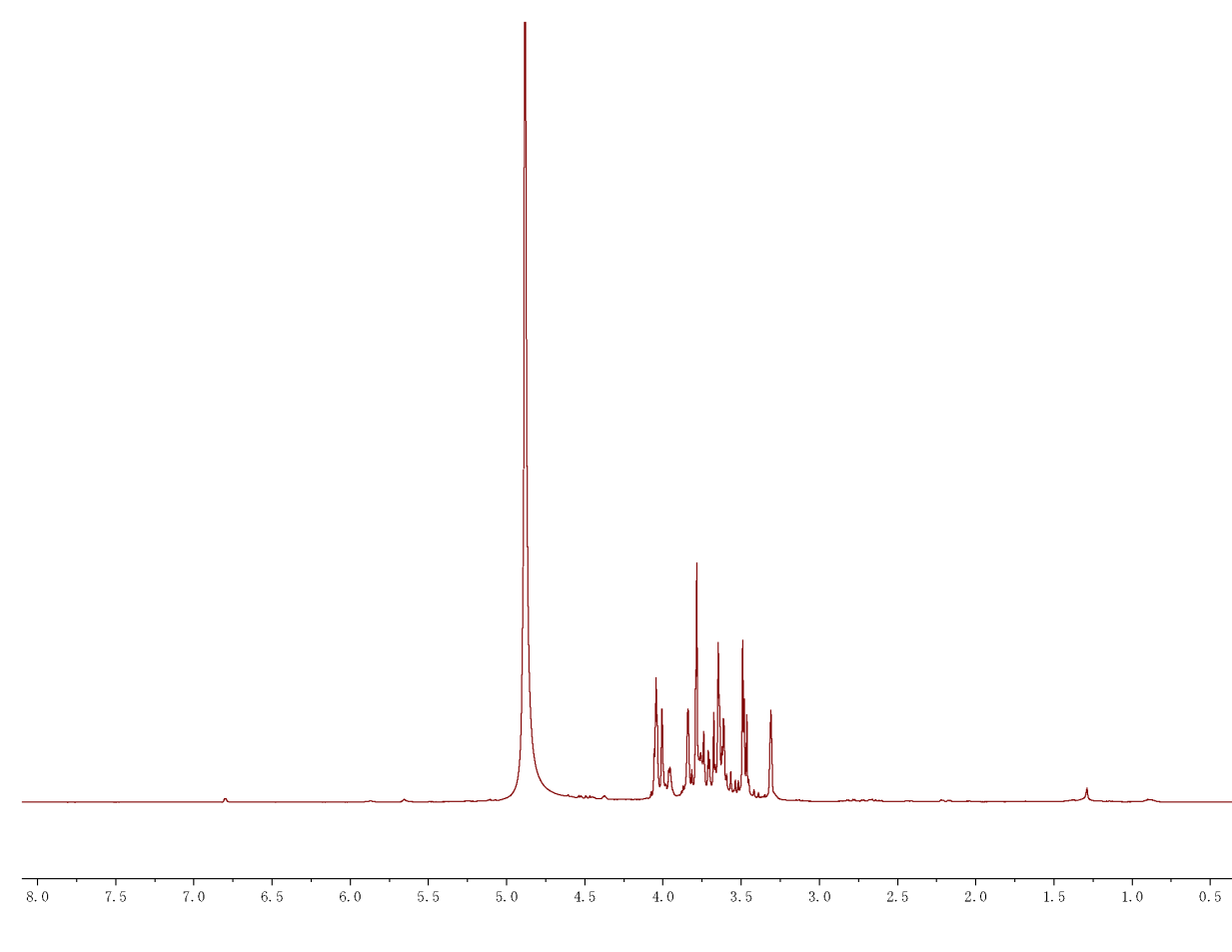
**

**Figure S16** ^1^H NMR for mixture of three fructose isomer (**7**) from *D. officinale*


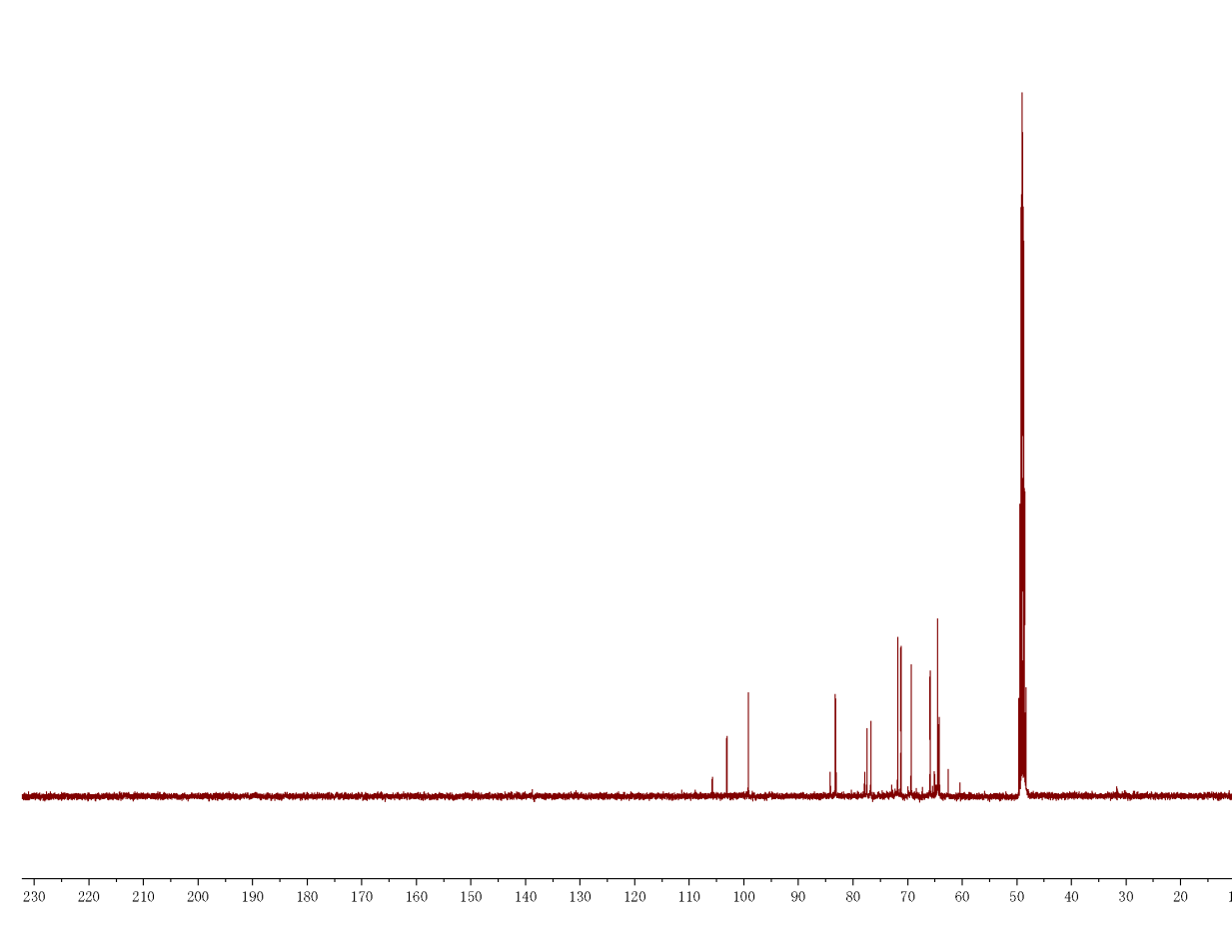


**Figure S17** ^13^C-NMR for mixture of three fructose isomer (**7**) from *D. officinale*

**
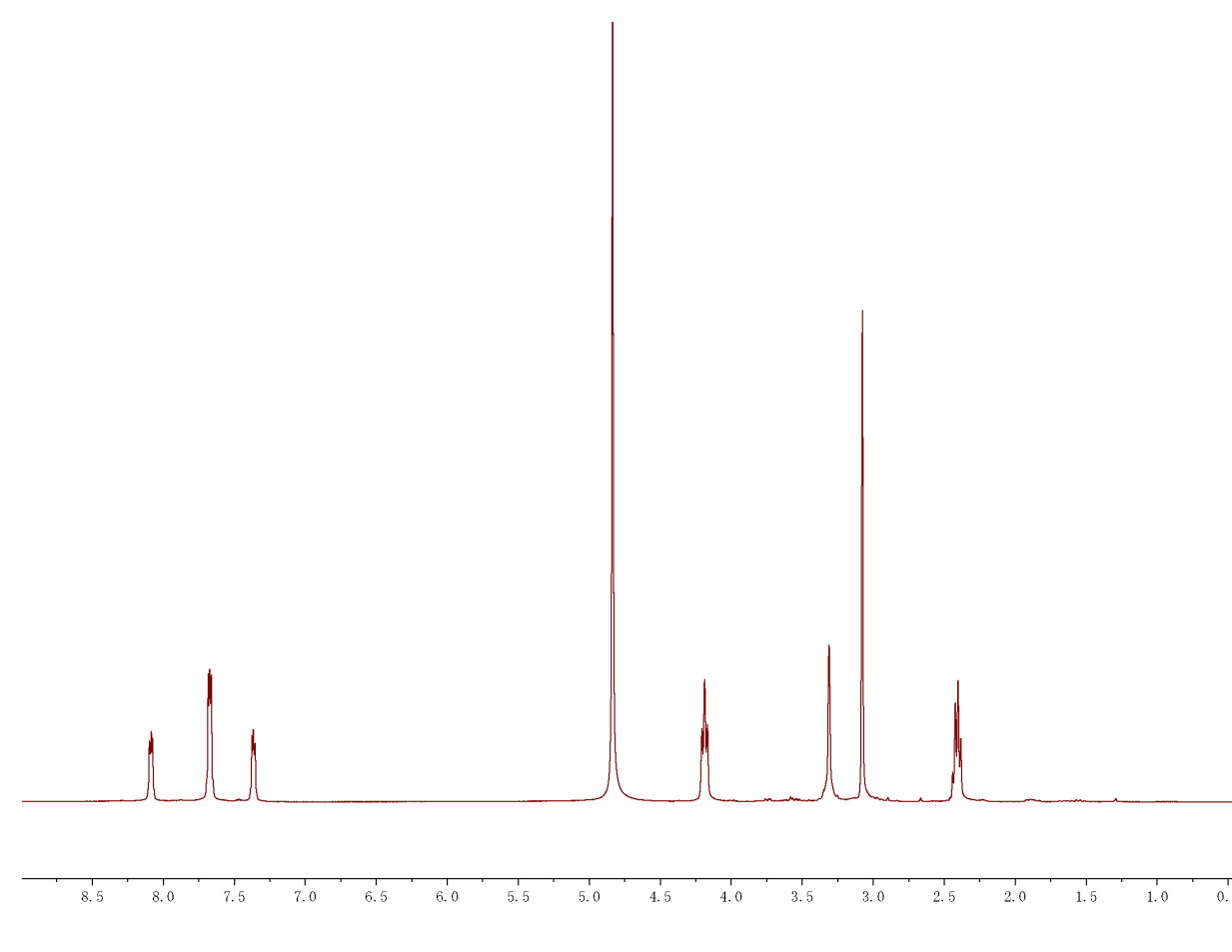
**

**Figure S18** ^1^H NMR for shihunine (**8**) from *D. loddigesii*


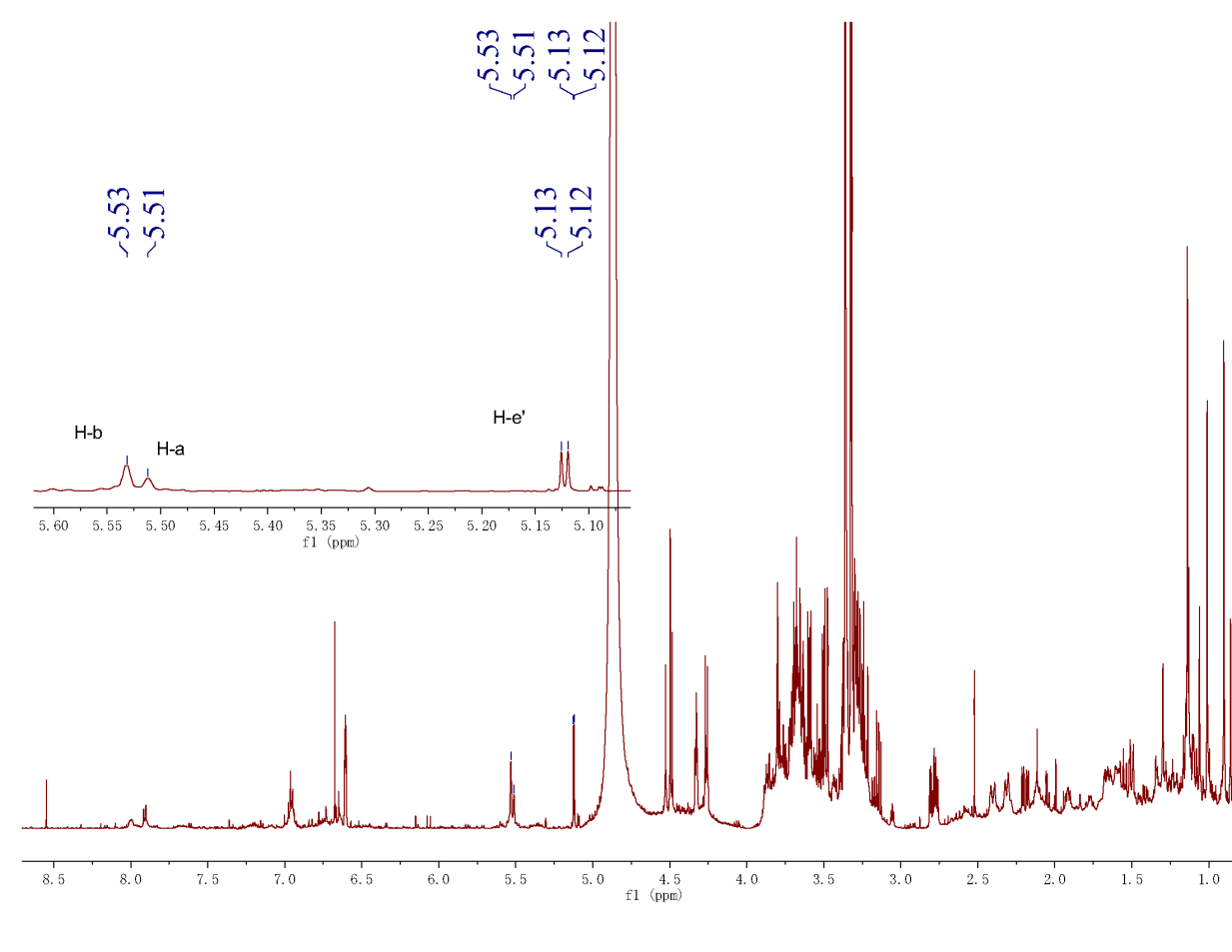


**Figure S19** ^1^H NMR of polar-extract-f for content determination


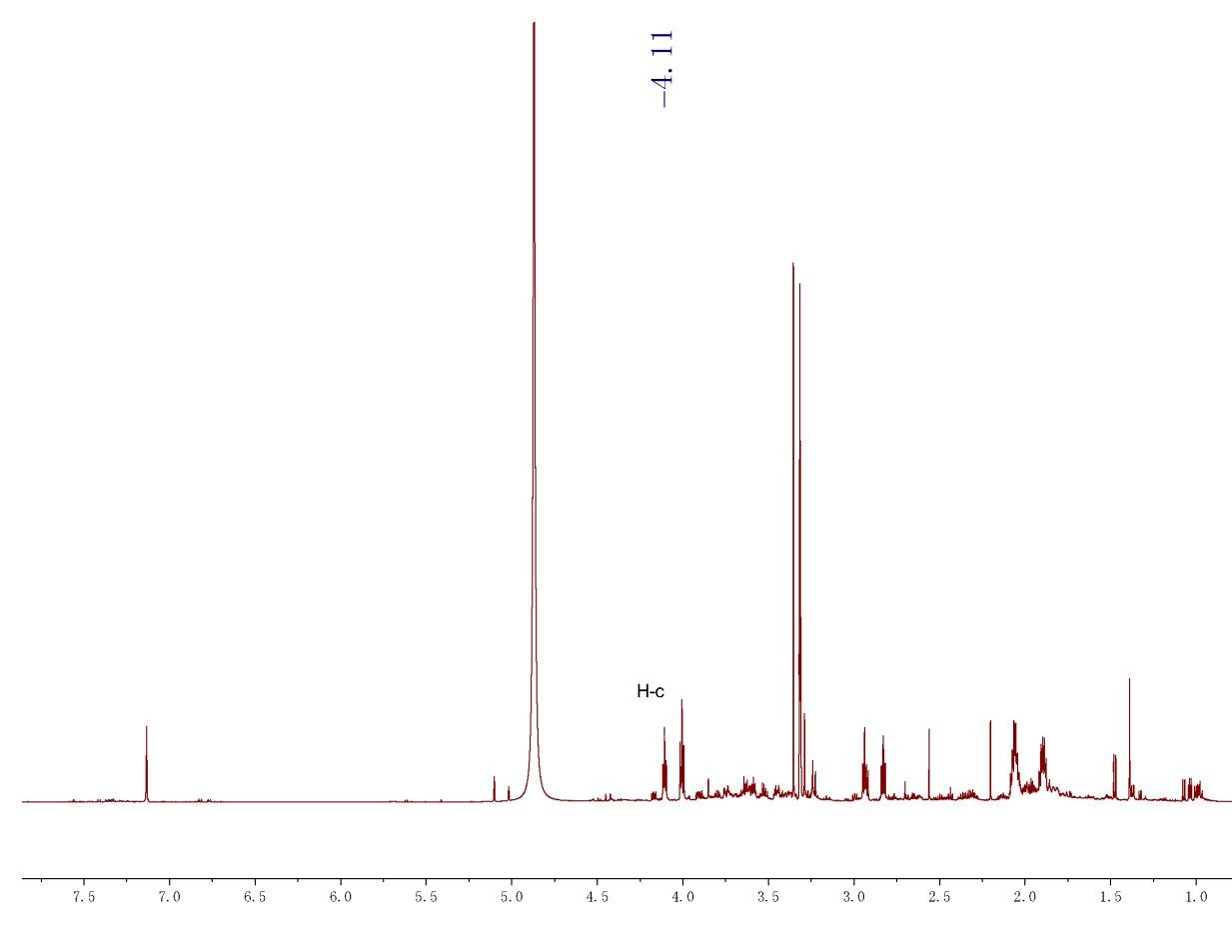


**Figure S20** ^1^H NMR of polar-extract-n for content determination


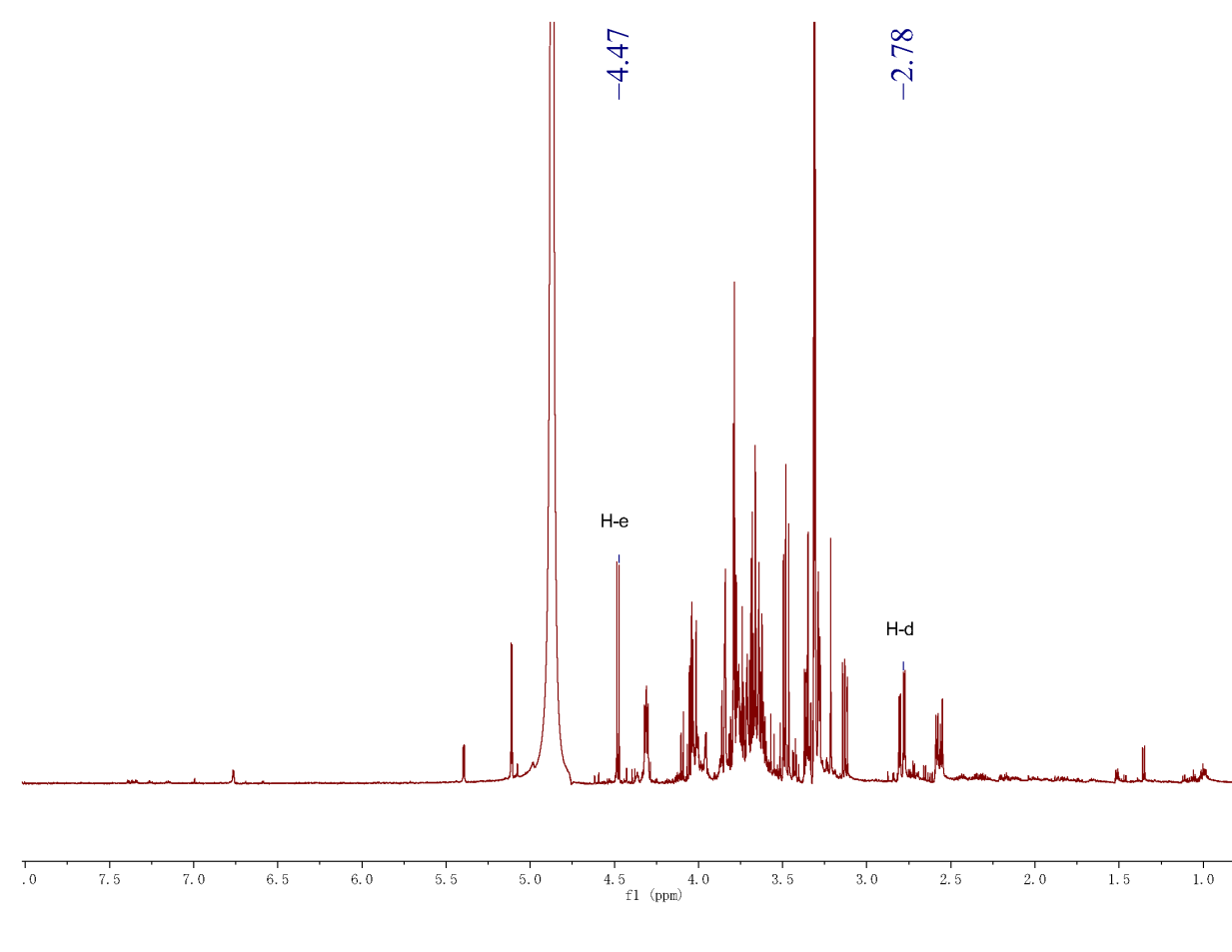


**Figure S21** ^1^H NMR of polar-extract-o for content determination


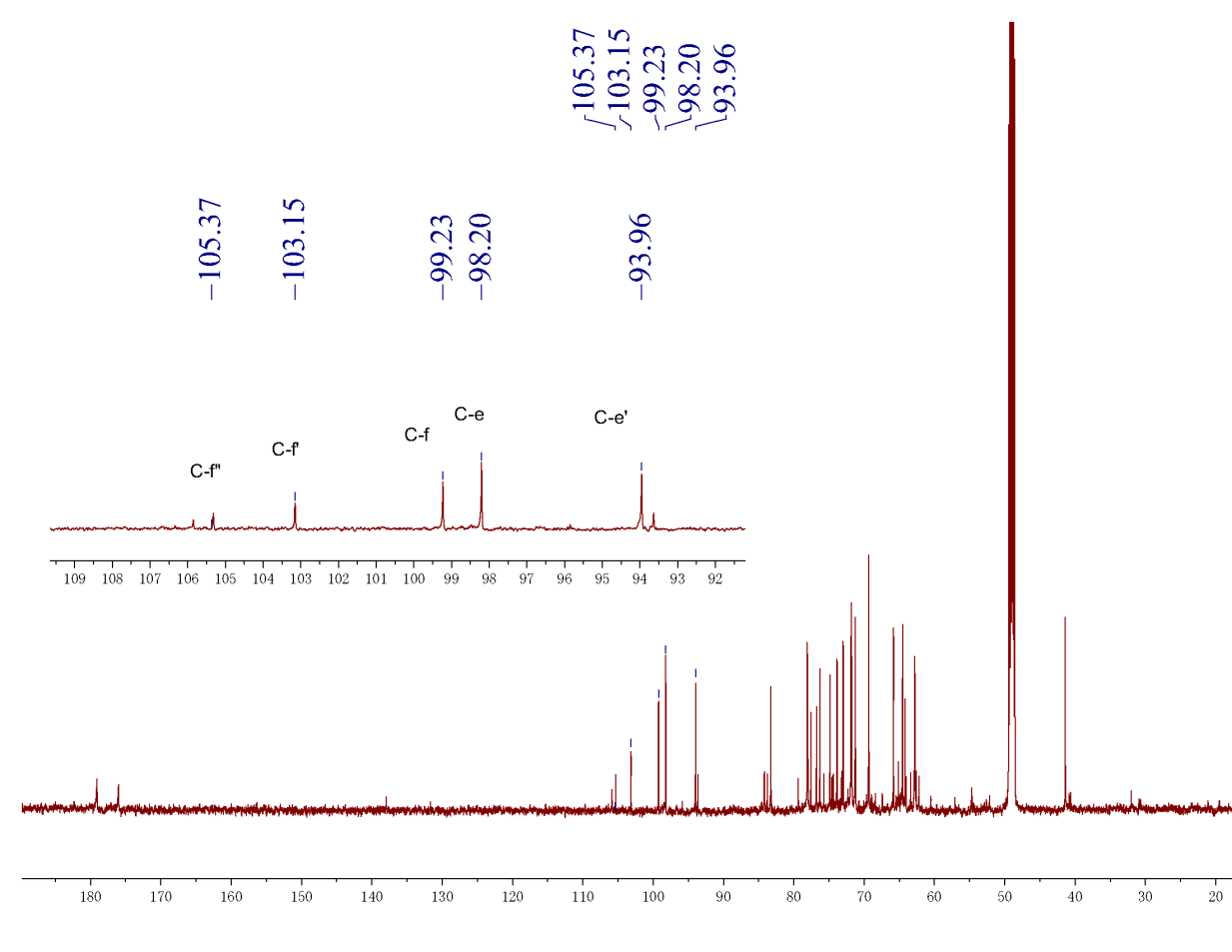


**Figure S22** ^13^C-NMR of polar-extract-o for content determination


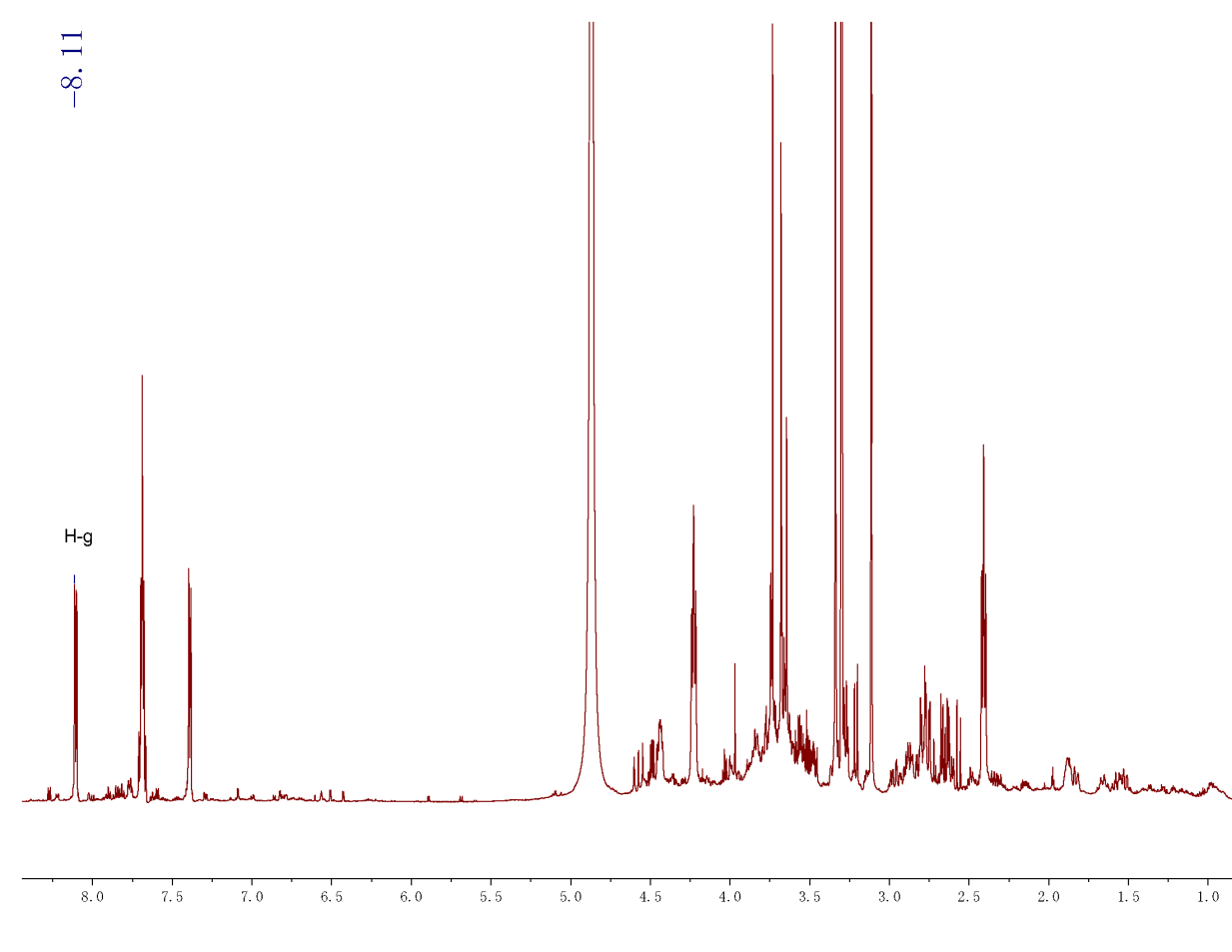


**Figure S23** ^1^H NMR of polar-extract-l for content determination


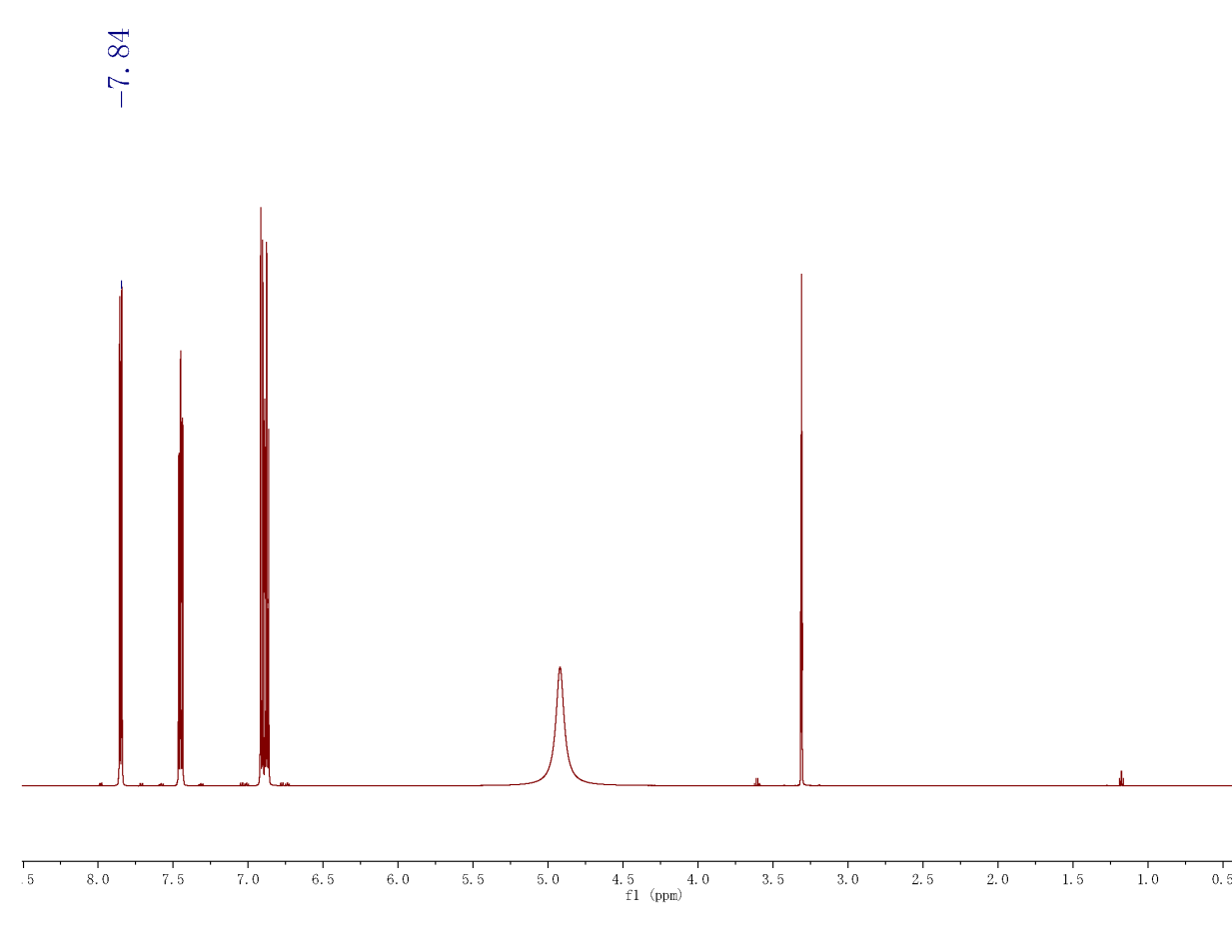


**Figure S24** ^1^H NMR of salicylic acid for an external standard

**Figure S25** The inhibitory activities of the polar extracts on α-glucosidase

**Figure S26** The inhibitory activities of isolated metabolites on α-glucosidase

**Table S1.**The chemical shifts and splitting patterns of diagnostic signals of isolated metabolites

| Plant | Compound | Diagnostic signals | |
| --- | --- | --- | --- |
|  |  | δ (ppm, J, Hz) | |
| *F. fimbriata* | flifimdioside A (1) | 5.51 (s, 1H-a), 1.13 (s, 3H), 1.06 (s, 3H), 0.86 (s, 3H), 0.72 (s, 3H). | |
|  | flickinflimoside B (2) | 5.54 (s, 1H-b), 1.14 (s, 3H), 1.01 (s, 3H), 0.90 (s, 3H), 0.76 (s, 3H). | |
|  | syringaresinol-4′-*O*-D-glucopyranoside (3) | / | |
|  | 3-*O*-*β*-D-galactopyranosyl-*β*-D-galactopyranose (6) | 5.12 (d, 2.4, 1H-e'). | |
| *D. nobile* | anosmine (4) | 7.14 (s, 1H), 4.11 (t, 3.6, 2H-c), 4.01 (t, 4.4, 2H), 2.94 (t, 4.4, 2H), 2.83 (t, 4.4, 2H), 2.07 (m 4H), 1.89 (m 4H) | |
| *D.officinale* | malic acid (5) | 4.31 (m 1H), 2.78 (dd, 10.8, 3.6, 2H-d), 2.57 (dd, 10.8, 4.8) | |
|  | 3-*O*-*β*-D-galactopyranosyl-β-D-galactopyranose (6) | 5.11 (d, 2.4, 1H-e'), 4.48 (d, 5.2, 1H-e) | |
|  | *β*-pyranose (7) | C-f/99.2 (s) |  |
|  | *β*-furanose (7) | C-f′/103.1 (s) |  |
|  | *α*- furanose (7) | C-f″/105.3 (s) |  |
| *D. loddigesii* | shihunine (8) | 8.11 (dd, 4.4, 1.2, 1H-g/), 7.69 (m, 2H), 7.39 (dd, 4.4, 1.2, 1H), 4.23 (t, 5.2, 2H), 2.41 (dd, 5.2, 2H) | |
