# Supplementary material for: Study on the Polar Extracts of Dendrobium nobile, D. officinale, D. loddigesii, and Flickingeria fimbriata: Metabolite Identification, Content Evaluation, and Bioactivity Assay
Source: Molecules. 2018 May 15;23(5):1185. doi: 10.3390/molecules23051185 (PMC6099805; doi:10.3390/molecules23051185)
Supplement: Supplementary file 1 [file molecules-23-01185-s001.zip › molecules-287670-supplementary/Supplementary Materials/Supplementary Materials.pdf]

Supplementary Materials

Study on the polar extracts of *Dendrobium nobile*, *D. officinale*, *D. loddigesii* and *Flickingeria fimbriata*: metabolite identification, content evaluation and bioactivity assay

Huiping Chen[1,†], Xuewen Li[1,†], Yongli Xu[1], Kakei Lo[1], Huizhen Zheng[1], Haiyan Hu[1,*], Jun Wang[1*] and Yongcheng Lin[2]

[1] School of Pharmaceutical Sciences, Sun Yat-sen University, Guangzhou 510006, China

[2] School of Chemistry and Chemical Engineering, Sun Yat-sen University, Guangzhou 510275, China

[*] Corresponding author

E-mail address: lsshhy@mail.sysu.edu.cn (H-Y. Hu), or, wjun@mail.sysu.edu.cn (J. Wang)

† Those authors contribute equally to this work.

Figure Legends:

Figure S1. Structures of **1**–**8** and their target protons (a–g) for content determination

Figure S2 ^{1}H NMR for flifimdioside A (**1**) from *F. fimbriata*

Figure S3 ^{13}C NMR for flifimdioside A (**1**) from *F. fimbriata*

Figure S4 ^{1}H-^{1}H COSY for flifimdioside A (**1**) from *F. fimbriata*

Figure S5 HSQC for flifimdioside A (**1**) from *F. fimbriata*

Figure S6 HMBC for flifimdioside A (**1**) from *F. fimbriata*

Figure S7 HRMS spectrometry for flifimdioside A (**1**) from *F. fimbriata*

Figure S8 ECD spectrum for flifimdioside A (**1**) from *F. fimbriata*

Figure S9 ^{1}H NMR for flickinflimoside B (**2**) from *F. fimbriata*

Figure S10 ^{1}H NMR for syringaresinol-4′-*O*-D-glucopyranoside (**3**) from *F. fimbriata*

Figure S11 ^{1}H NMR for anosmine (**4**) from *D. nobile*

Figure S12 ^{1}H NMR for malic acid (**5**) from *D.officinale*

Figure S13 ^{1}H NMR for compound (**6**) from *D.officinale*

Figure S14 ^{13}C-NMR for compound (**6**) from *D.officinale*

Figure S15 ^{13}C-NMR for compound (**6**) from *F. fimbriata*

Figure S16 ^{1}H NMR for mixture of three fructose isomer (**7**) from *D.officinale*

Figure S17 ^{13}C NMR the mixture of three fructose isomer (**7**) from *D.officinale*

Figure S18 ^{1}H NMR for shihunine (**8**) from *D. loddigesii*

Figure S19 ^{1}H NMR of polar-extract-f for content determination

Figure S20 ^{1}H NMR of polar-extract-n for content determination

Figure S21 ^{1}H NMR of polar-extract-o for content determination

Figure S22 ^{13}C NMR of polar-extract-o for content determination

Figure S23 ^{1}H NMR of polar-extract-l for content determination

Figure S24 ^{1}H NMR of salicylic acid for an external standard

All NMR data were recorded in CD_3OD.

Figure S25 The inhibitory activities of the polar extracts on α-glucosidase

Figure S26 The inhibitory activities of isolated metabolites on α-glucosidase

Table S1 The chemical shifts and splitting patterns of diagnostic signals of isolated metabolites

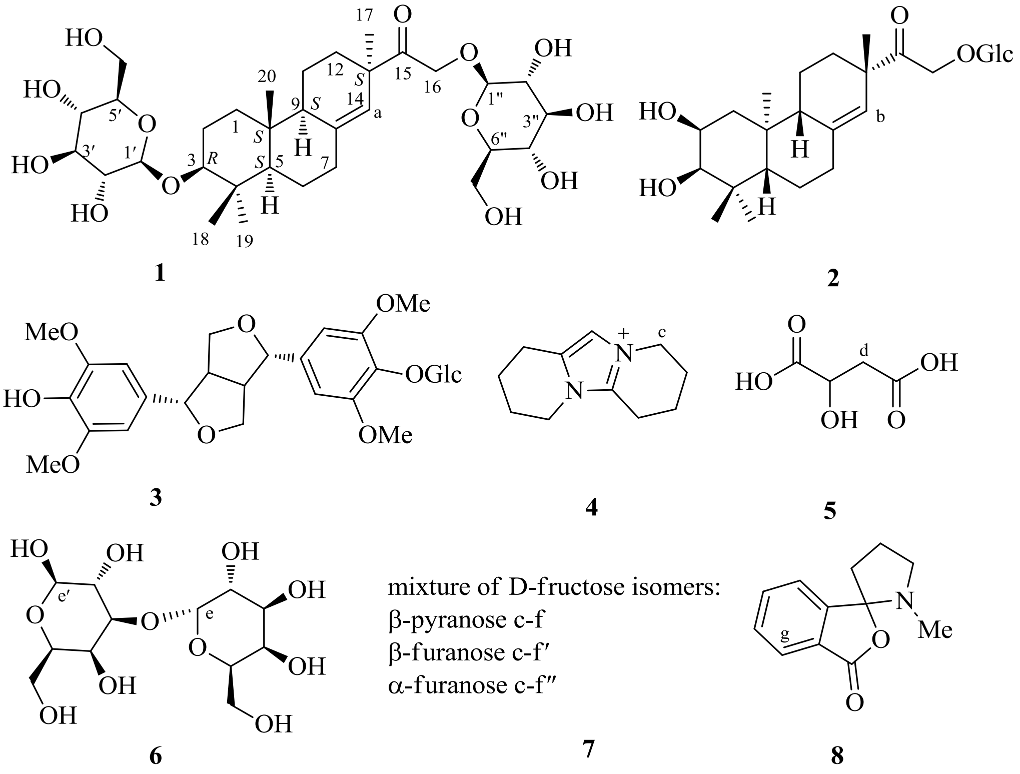

Figure S1. Structures of **1–8** and their target protons (a–g) for content determination

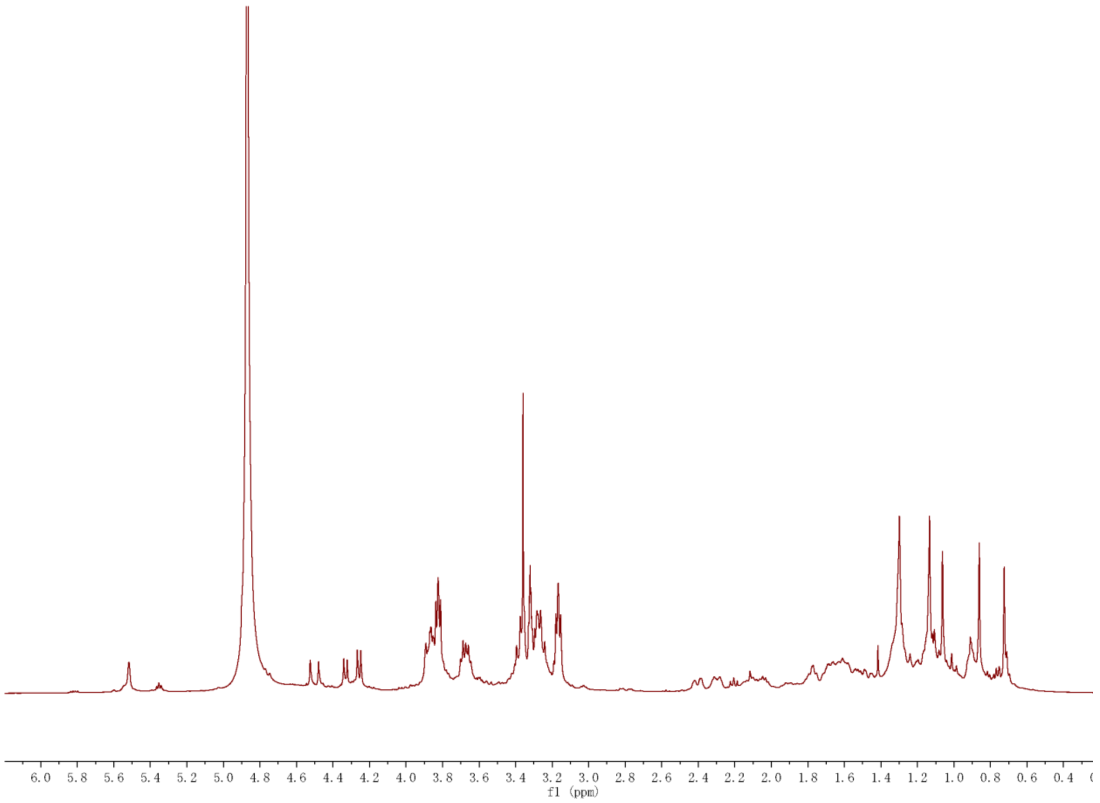

Figure S2 ^{1}H NMR for flifimdioside A (**1**) from *F. fimbriata*

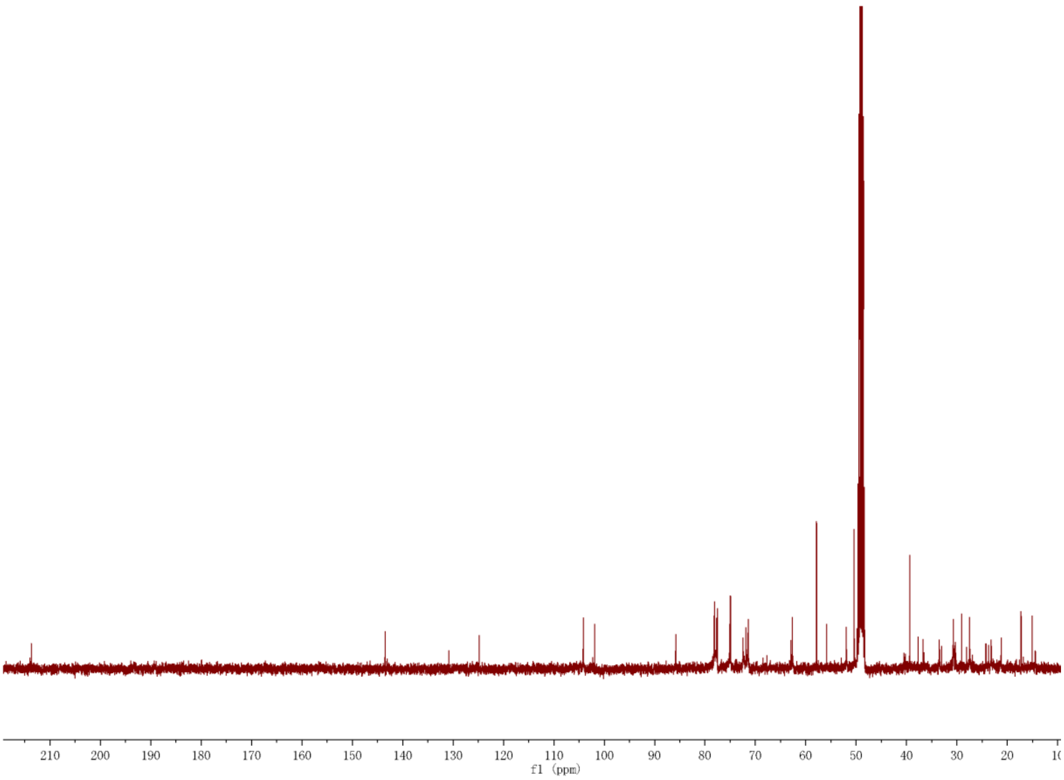

Figure S3 ^{13}C NMR for flifimdioside A (**1**) from *F. fimbriata*

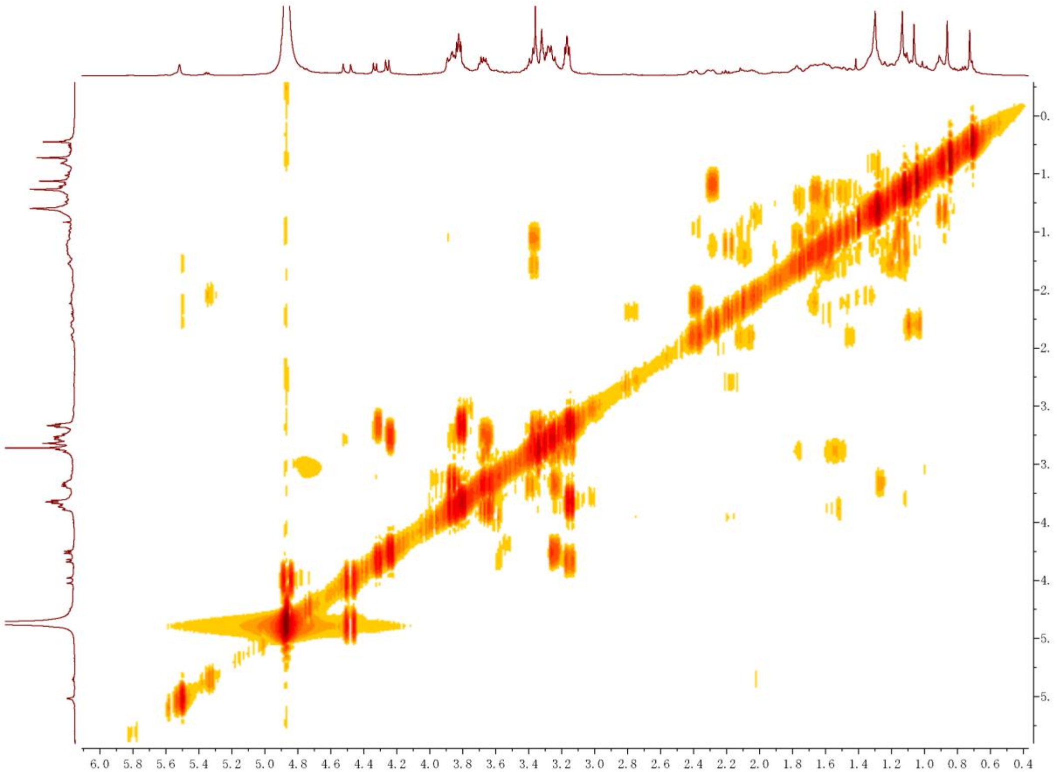

Figure S4 ^{1}H-^{1}H COSY for flifimdioside A (**1**) from *F. fimbriata*

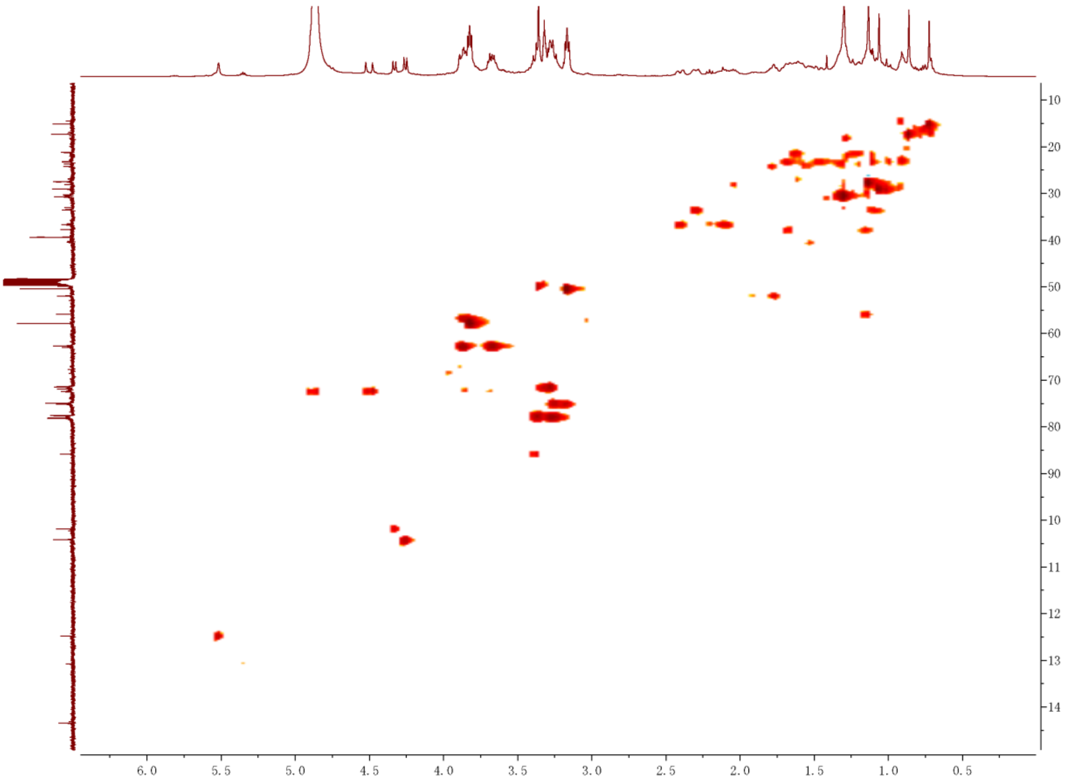

Figure S5 HSQC for flifimdioside A (**1**) from *F. fimbriata*

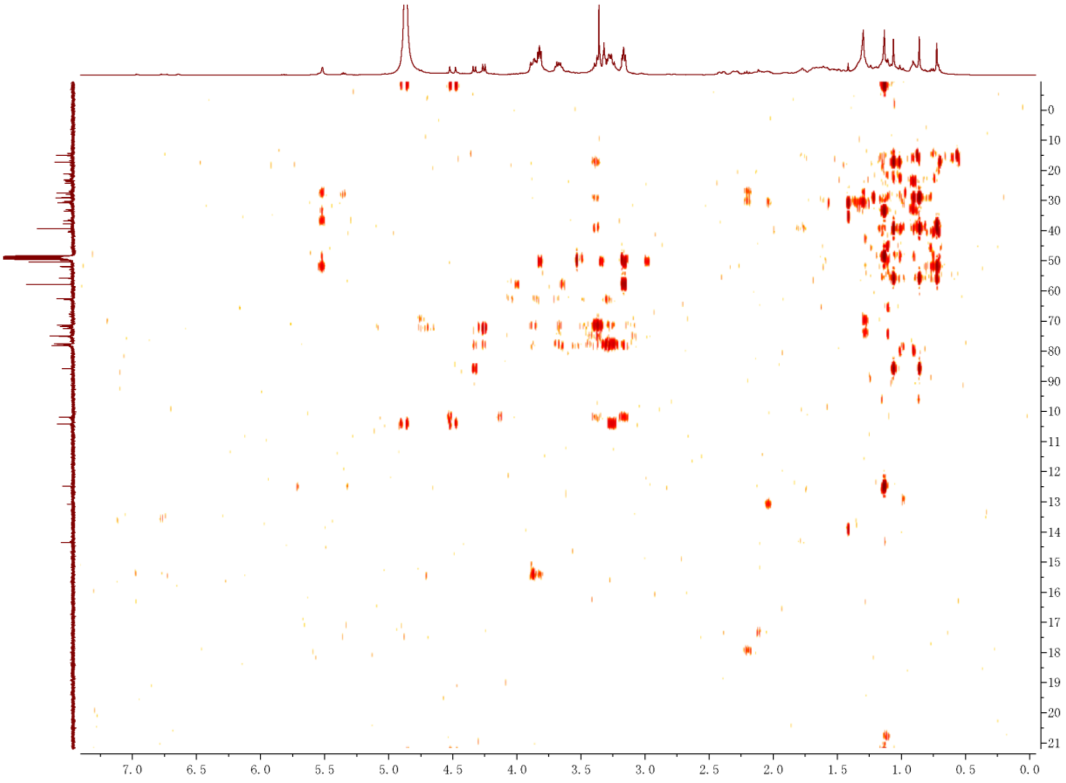

Figure S6 HMBC for flifimdioside A (**1**) from *F. fimbriata*

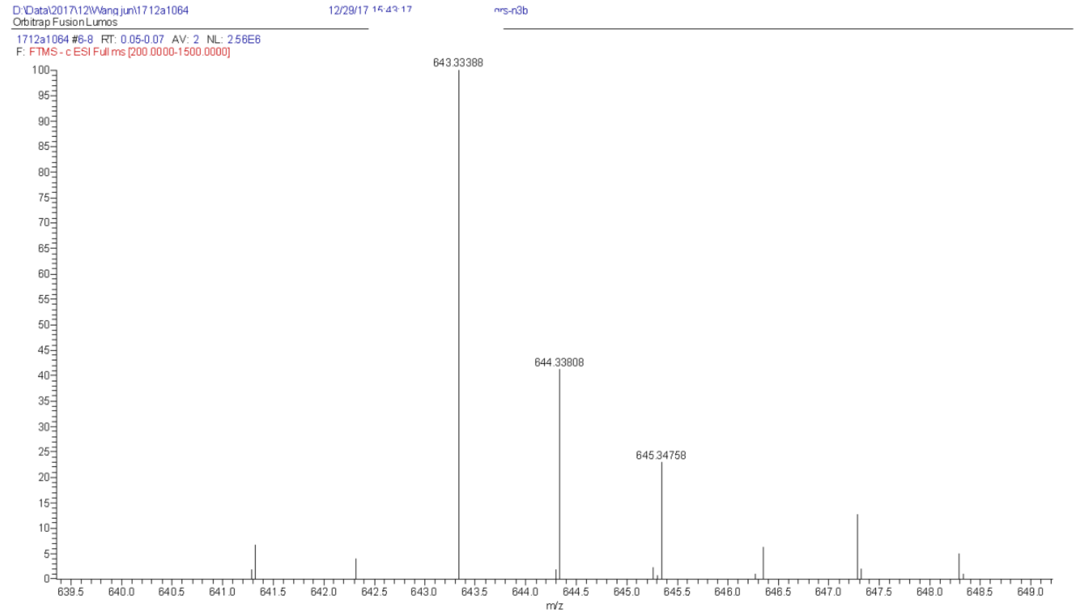

SPECTRUM - simulation :				
m/z	Theo. Mass	Delta (ppm)	RDB equiv.	Composition
643.33388	643.33351	0.57	7.5	C32 H51 O13

Limits:

1) Charge: -1
2) Nitrogen-role: Do not use
3) Mass tolerance: 5 ppm
4) Element in use: $^{12}C(0\sim35)$, $^{1}H(0\sim60)$, $^{16}O(0\sim15)$

Figure S7 HRMS spectrometry for flifimdioside A (**1**) from *F. fimbriata*

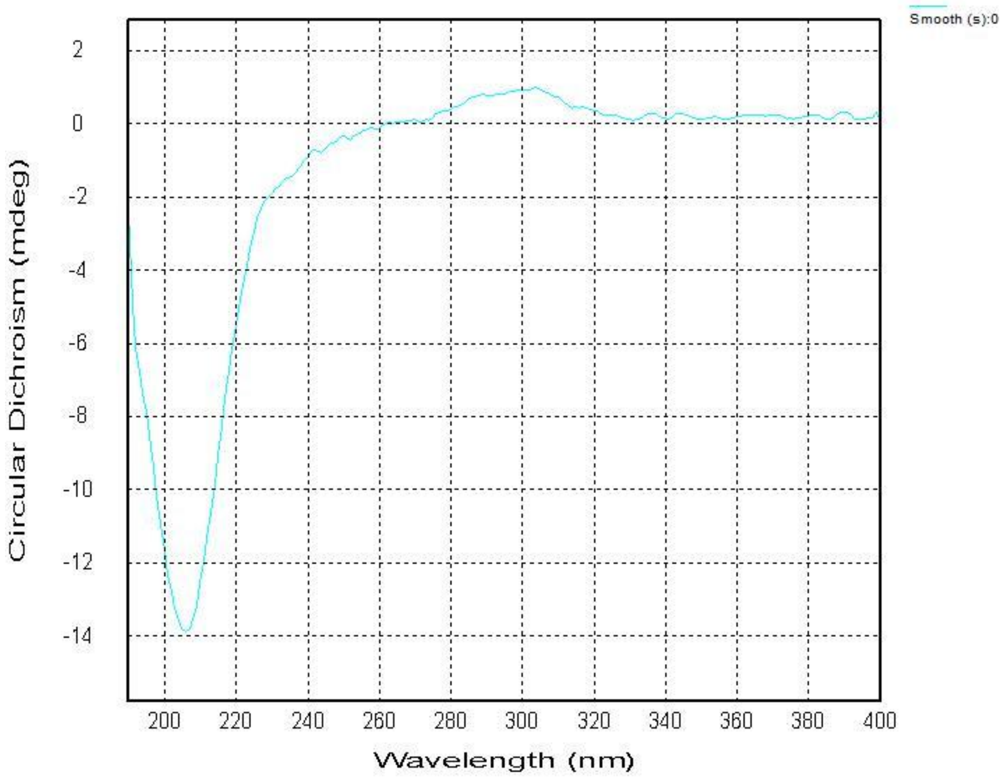

Figure S8 ECD spectra for flifimdioside A (**1**) from *F. fimbriata*

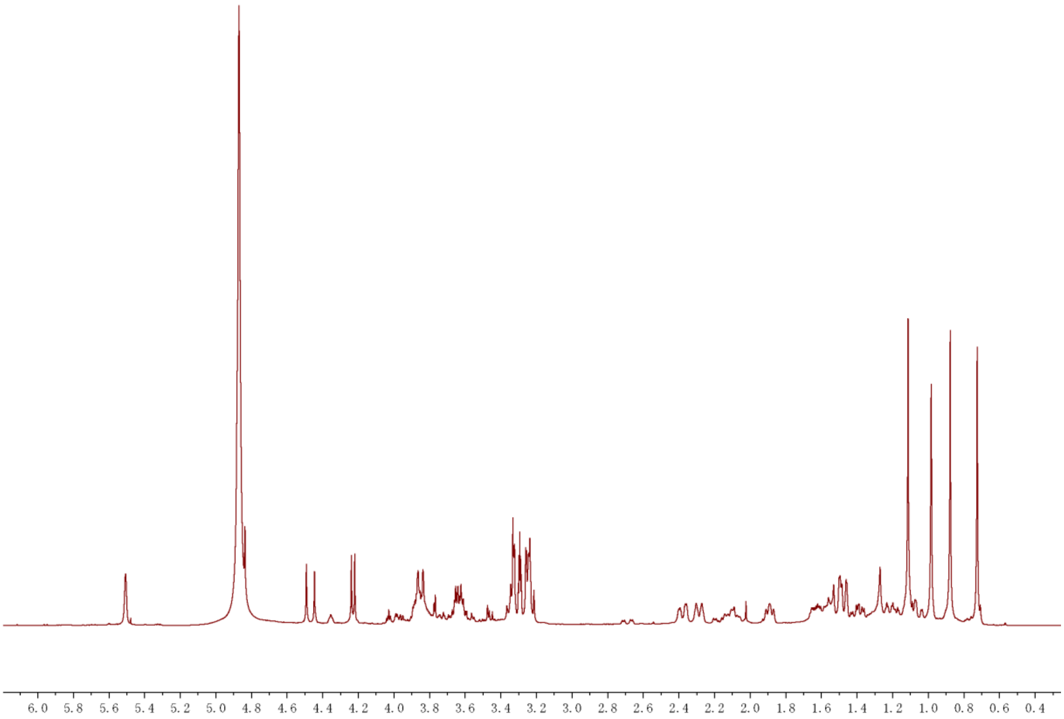

Figure S9 ^{1}H NMR for flickinflimoside B (**2**) from *F. fimbriata*

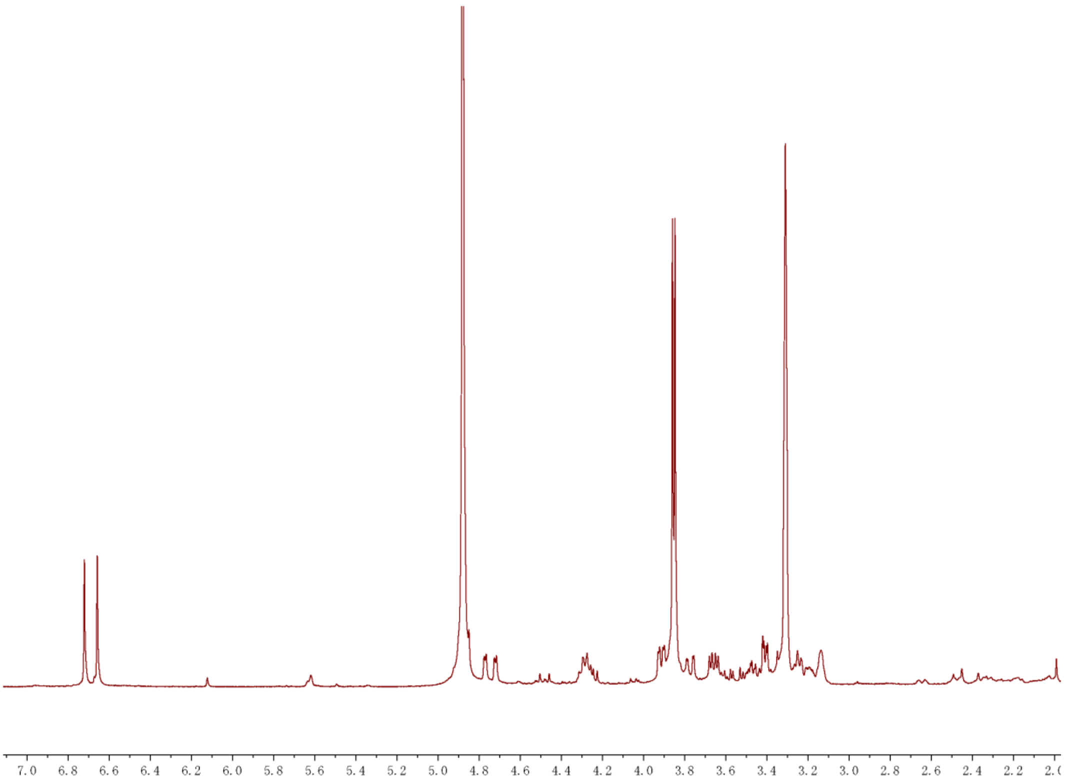

Figure S10 ^{1}H NMR for syringaresinol-4′-*O*-D-glucopyranoside (**3**) from *F. fimbriata*

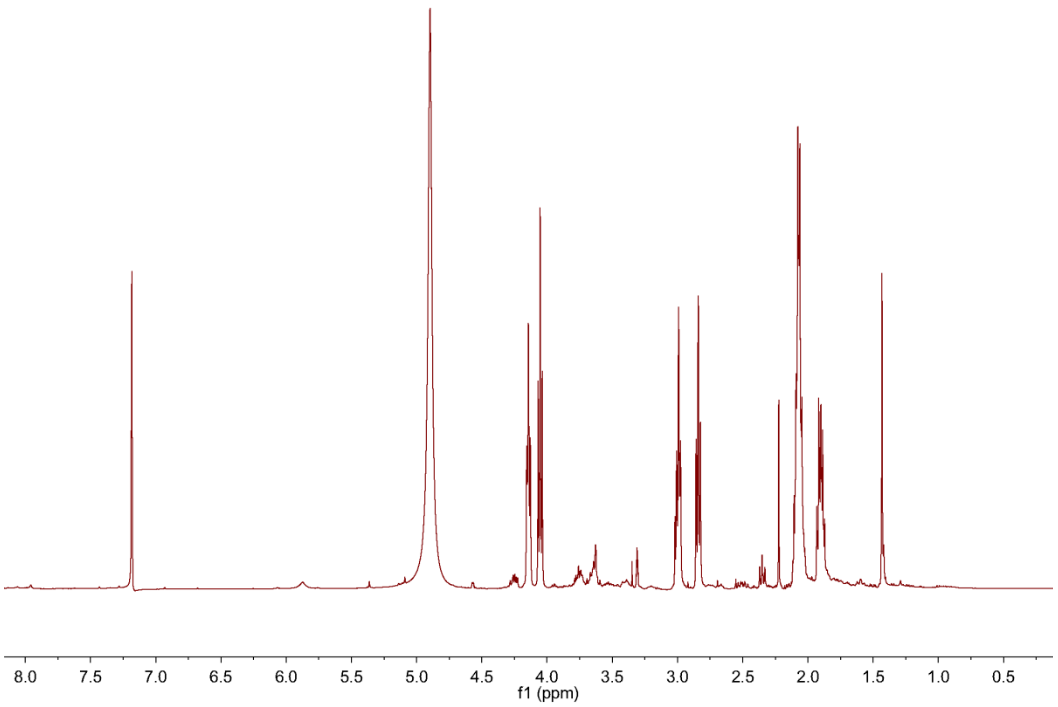

Figure S11 ^{1}H NMR for anosmine (**4**) from *D. nobile*

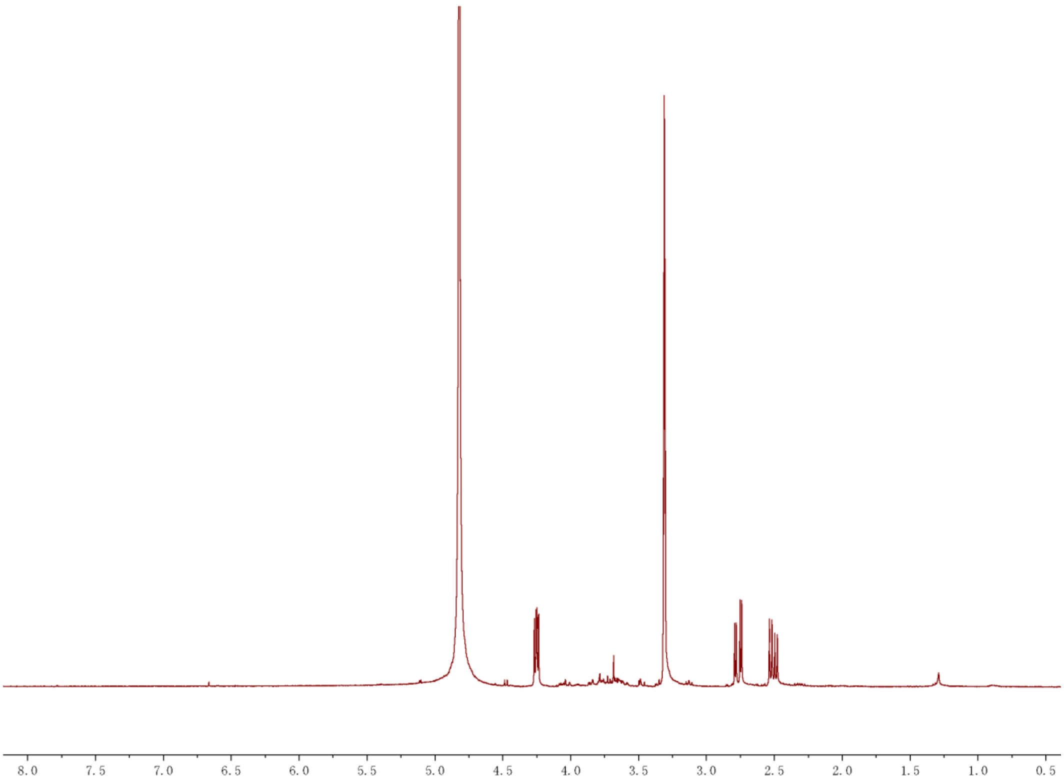

Figure S12 ^{1}H NMR for malic acid (**5**) from *D. officinale*

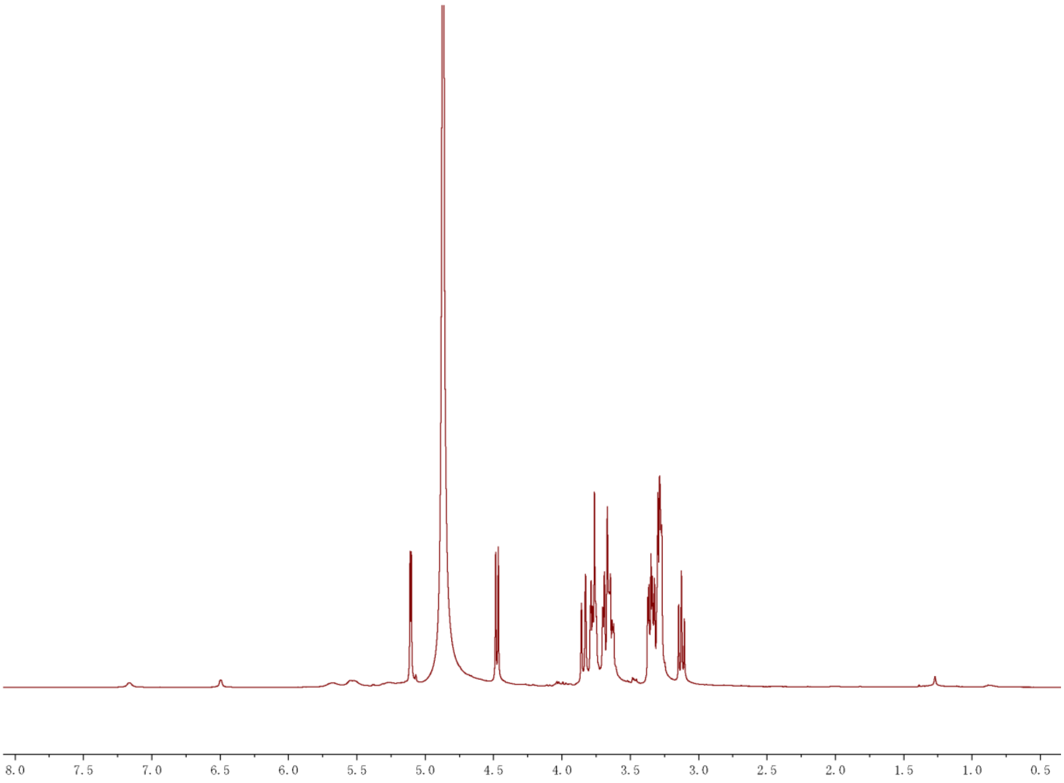

Figure S13 ^{1}H NMR for compound (**6**) from *D. officinale*

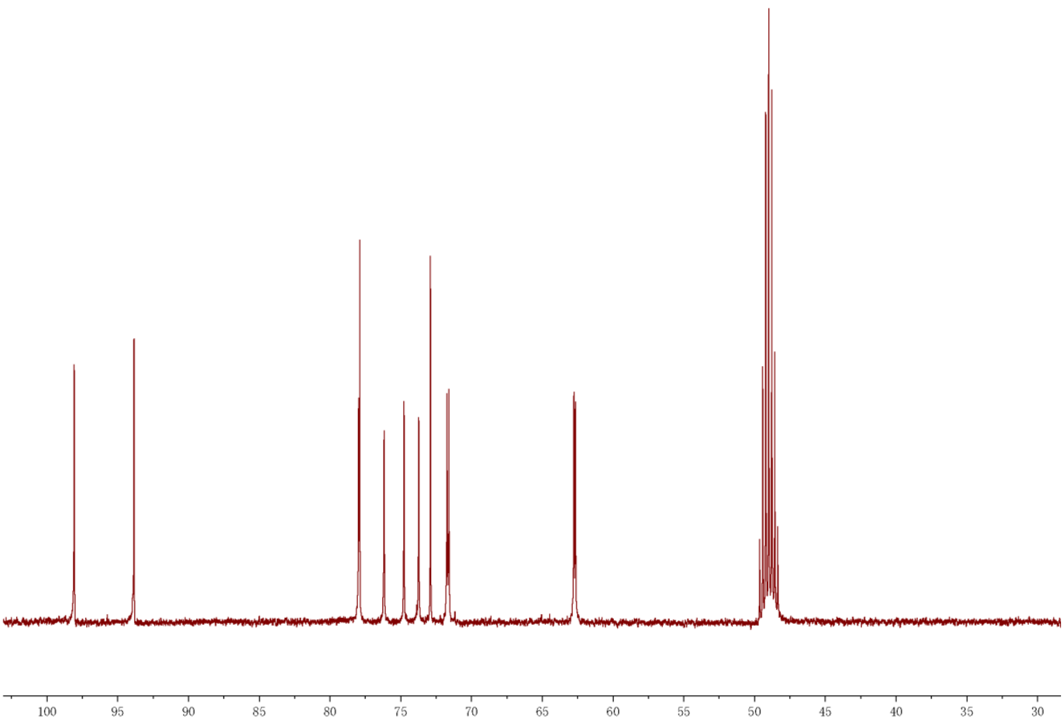

Figure S14 ^{13}C-NMR for compound (**6**) from *D. officinale*

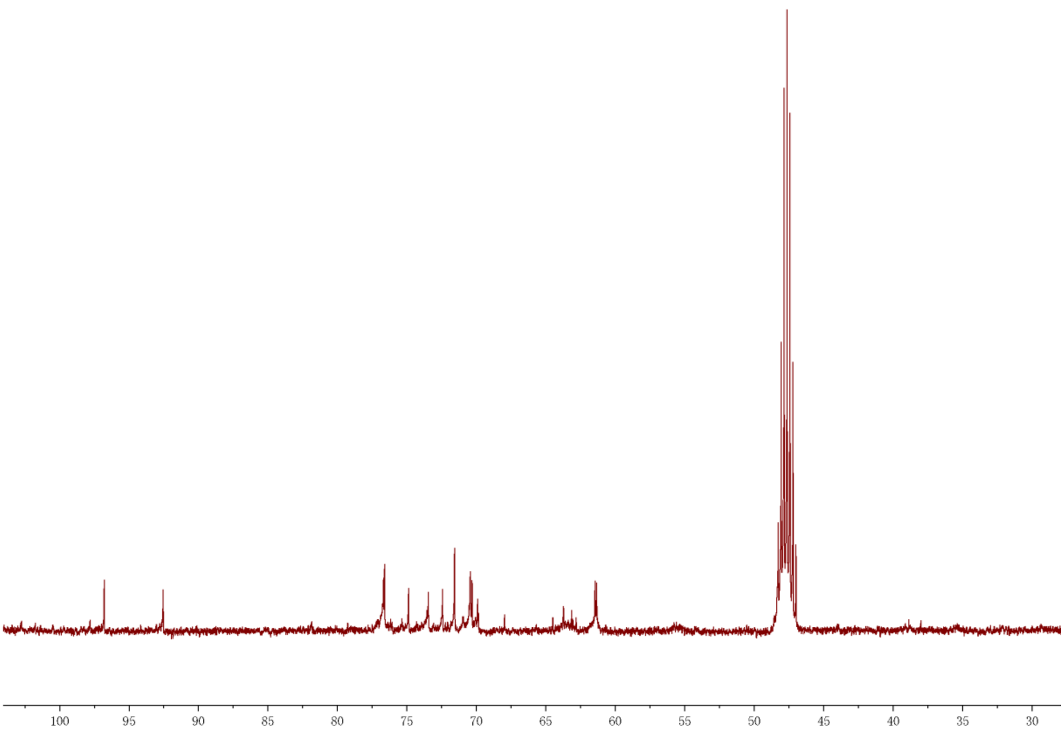

Figure S15 ^{13}C-NMR for compound (**6**) from *F. fimbriata*

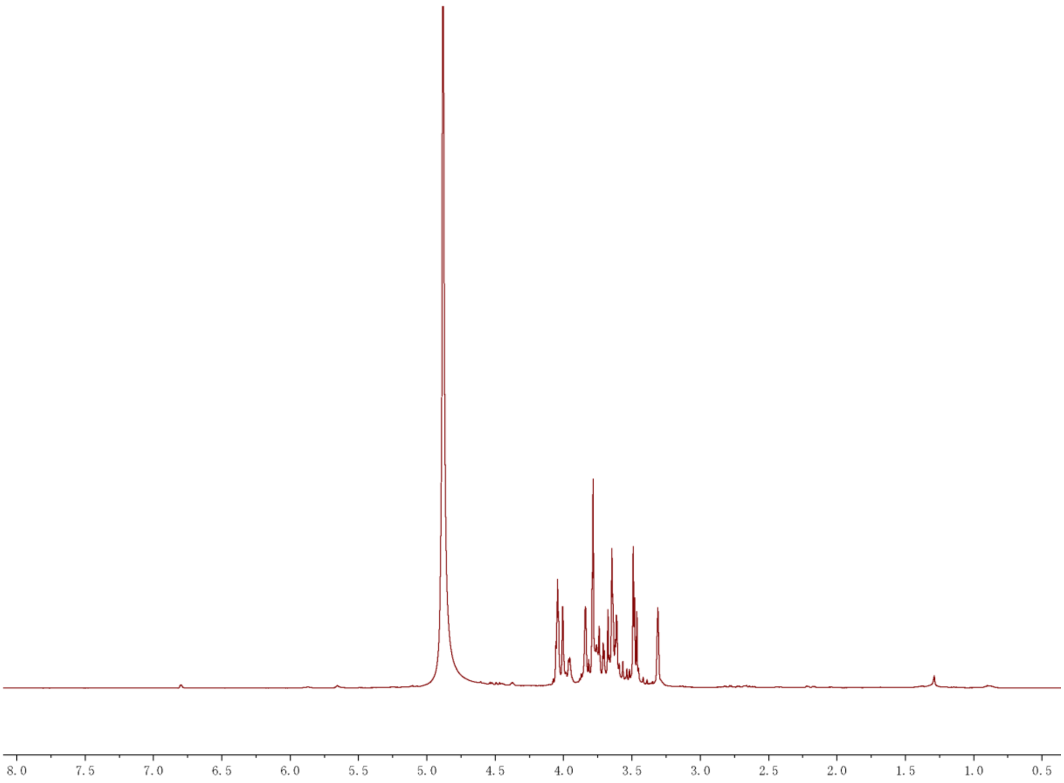

Figure S16 ^{1}H NMR for mixture of three fructose isomer (**7**) from *D. officinale*

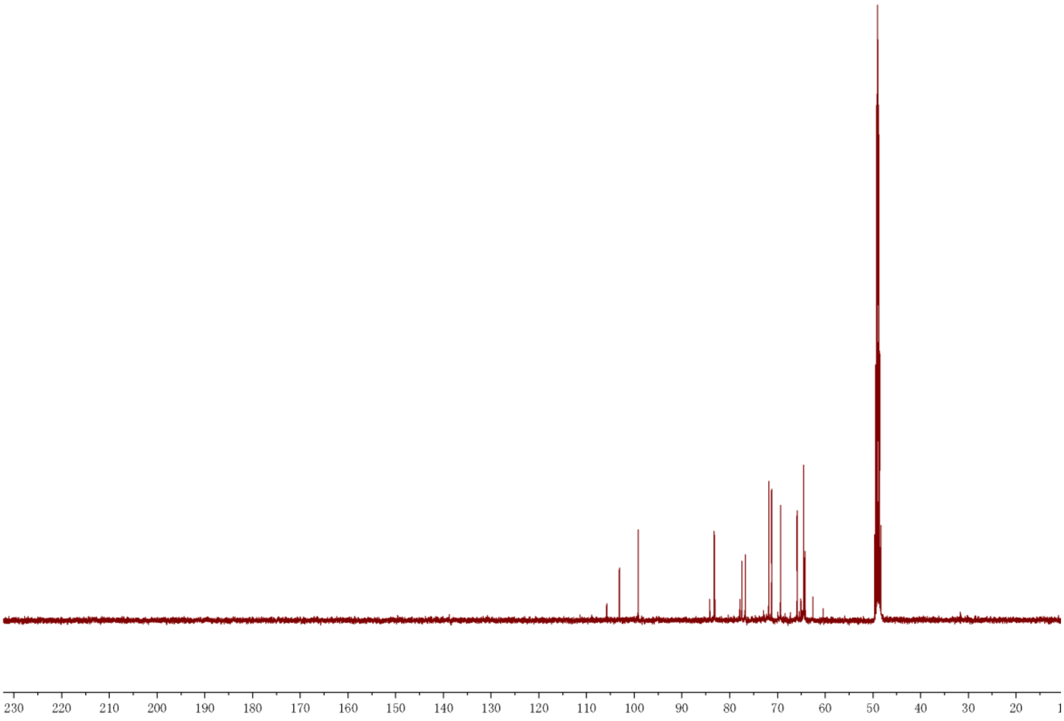

Figure S17 ^{13}C-NMR for mixture of three fructose isomer (**7**) from *D. officinale*

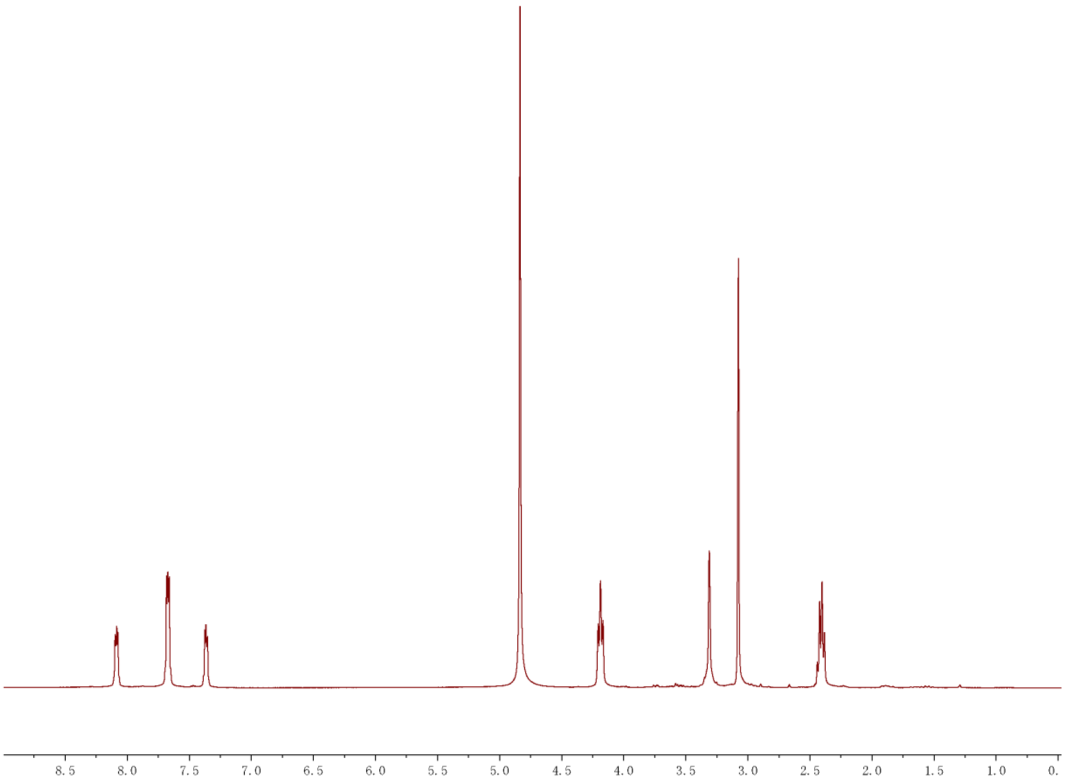

Figure S18 ¹H NMR for shihunine (**8**) from *D. loddigesii*

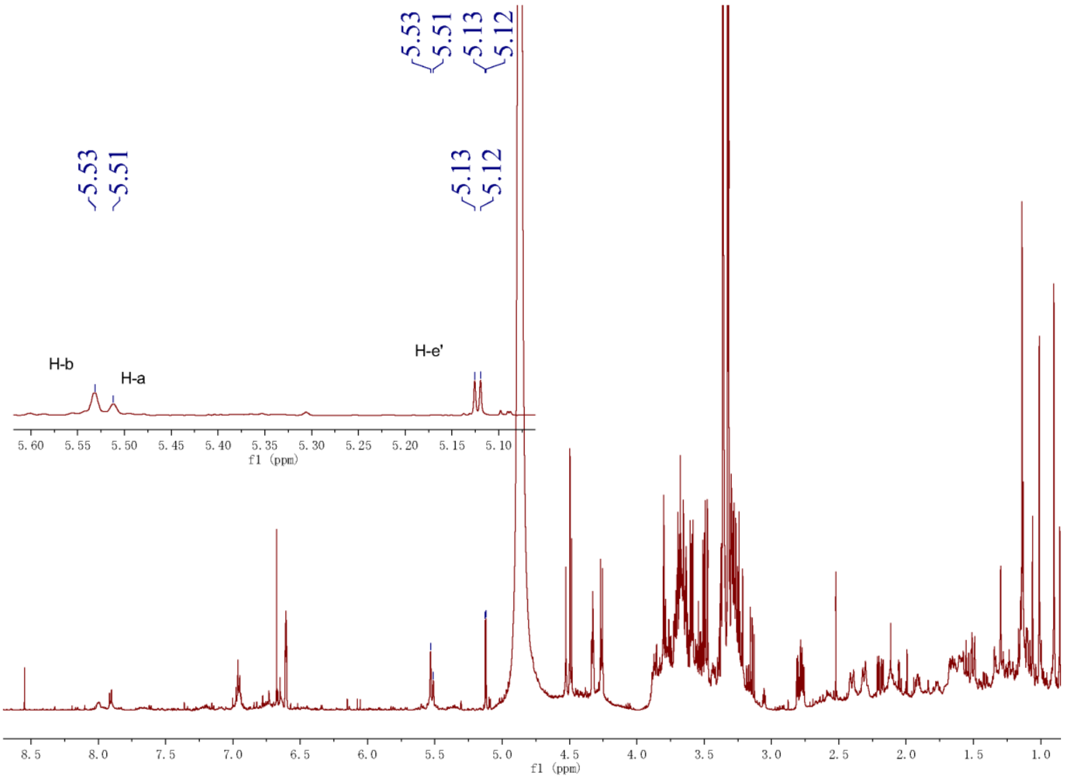

Figure S19 ¹H NMR of polar-extract-f for content determination

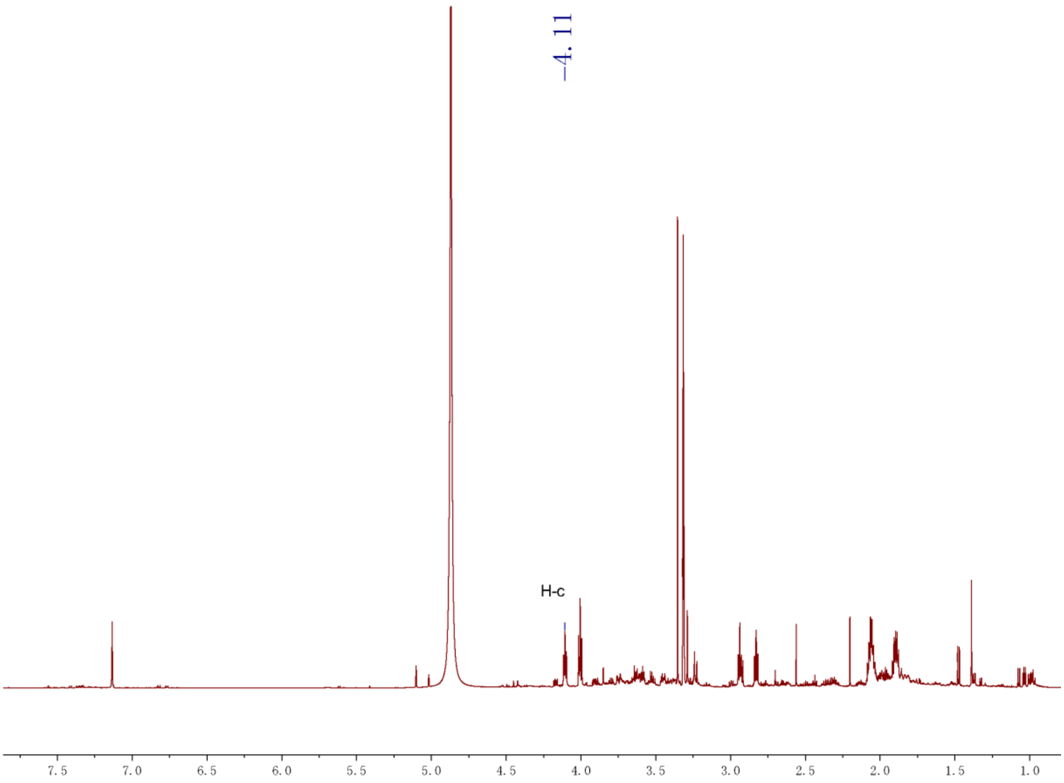

Figure S20 ^{1}H NMR of polar-extract-n for content determination

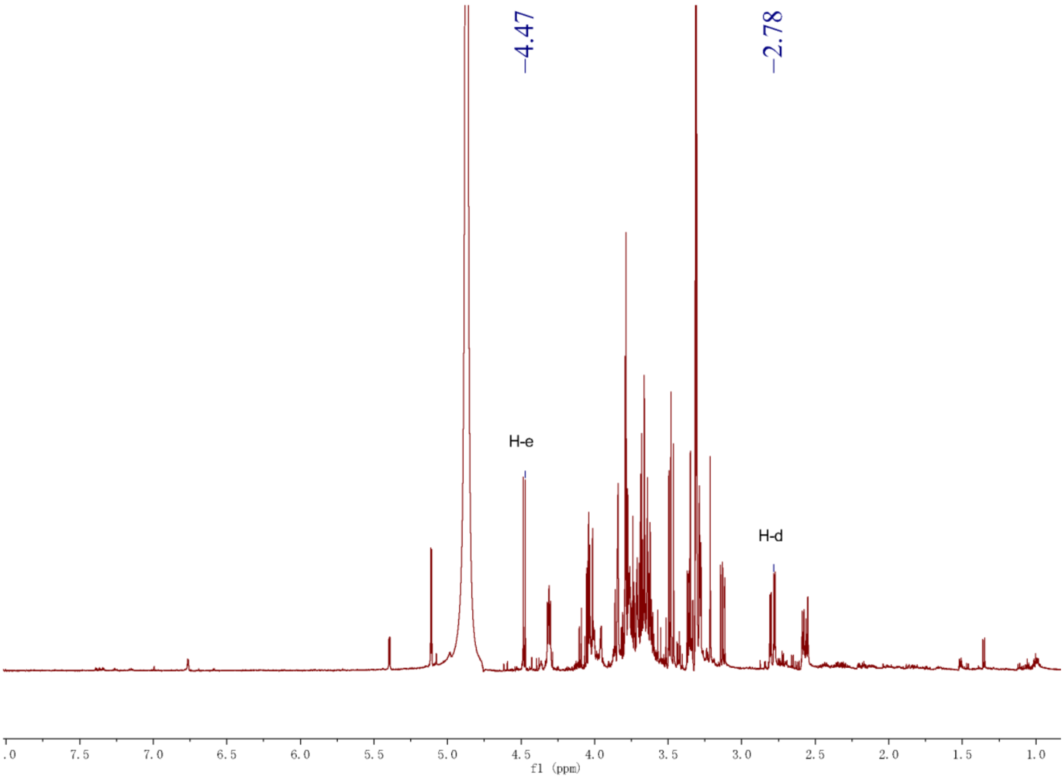

Figure S21 ^{1}H NMR of polar-extract-o for content determination

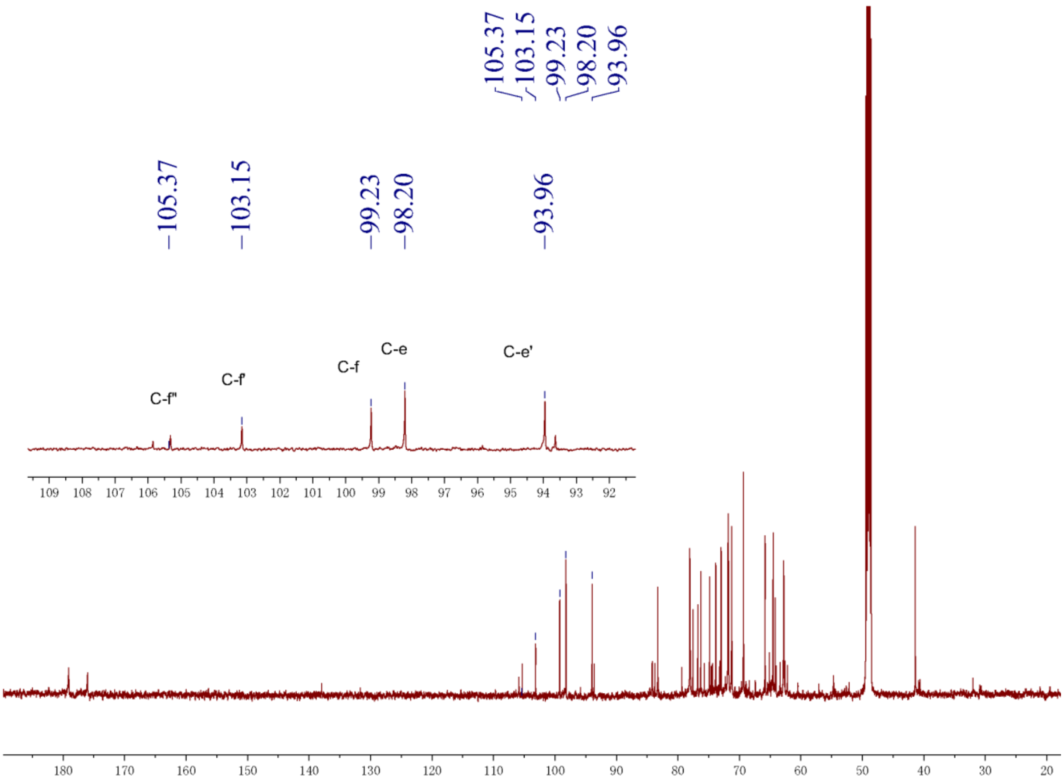

Figure S22 ^{13}C-NMR of polar-extract-o for content determination

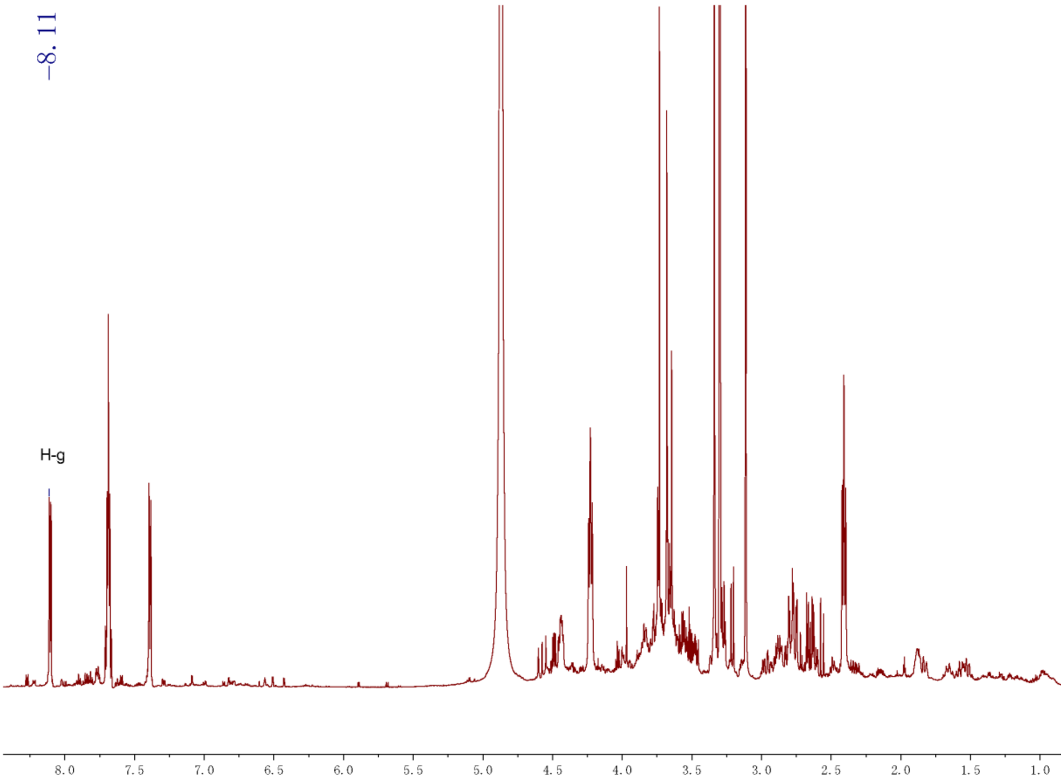

Figure S23 ^{1}H NMR of polar-extract-l for content determination

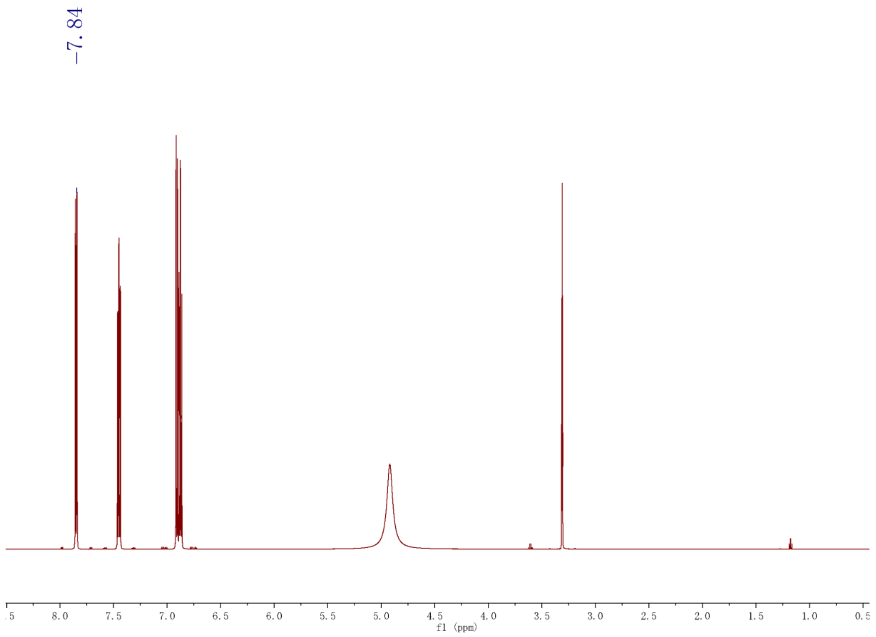

Figure S24 ^{1}H NMR of salicylic acid for an external standard

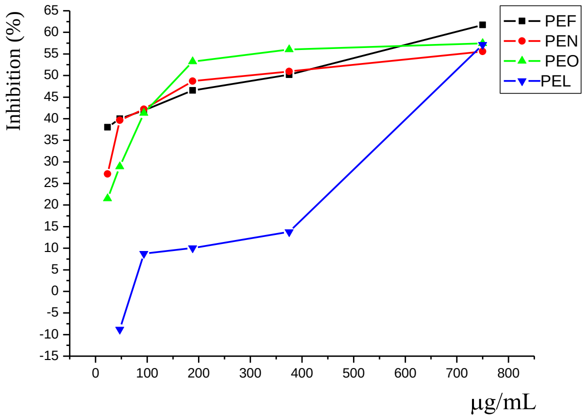

Figure S25 The inhibitory activities of the polar extracts on *α*-glucosidase

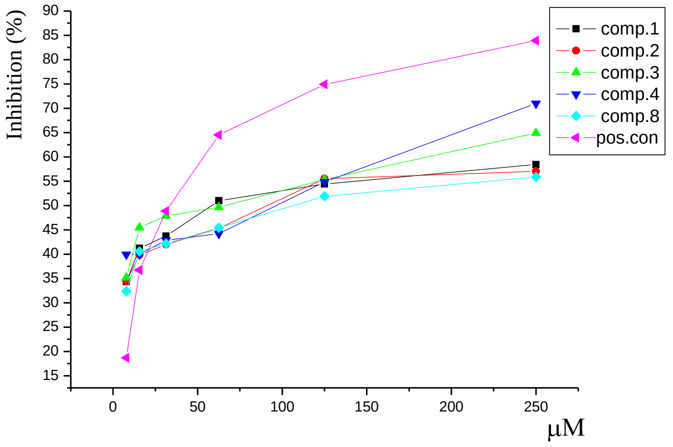

Figure S26 The inhibitory activities of isolated metabolites on *α*-glucosidase

Table S1. The chemical shifts and splitting patterns of diagnostic signals of isolated metabolites

Plant	Compound	Diagnostic signals δ (ppm, J, Hz)
F. fimbriata	flifimdioside A (**1**)	5.51 (s, 1H-a), 1.13 (s, 3H), 1.06 (s, 3H), 0.86 (s, 3H), 0.72 (s, 3H).
	flickinflimoside B (**2**)	5.54 (s, 1H-b), 1.14 (s, 3H), 1.01 (s, 3H), 0.90 (s, 3H), 0.76 (s, 3H).
	syringaresinol-4′-*O*-D-glucopyranoside (**3**)	/
	3-*O*-*β*-D-galactopyranosyl-*β*-D-galactopyranose (**6**)	5.12 (d, 2.4, 1H-e').
D. nobile	anosmine (**4**)	7.14 (s, 1H), 4.11 (t, 3.6, 2H-c), 4.01 (t, 4.4, 2H), 2.94 (t, 4.4, 2H), 2.83 (t, 4.4, 2H), 2.07 (m 4H), 1.89 (m 4H)
D.officinale	malic acid (**5**)	4.31 (m 1H), 2.78 (dd, 10.8, 3.6, 2H-d), 2.57 (dd, 10.8, 4.8)
	3-*O*-*β*-D-galactopyranosyl-β-D-galactopyranose (**6**)	5.11 (d, 2.4, 1H-e'), 4.48 (d, 5.2, 1H-e)
	β-pyranose (**7**)	C-f/99.2 (s)
	β-furanose (**7**)	C-f′/103.1 (s)
	α- furanose (**7**)	C-f″/105.3 (s)
D. loddigesii	shihunine (**8**)	8.11 (dd, 4.4, 1.2, 1H-g/), 7.69 (m, 2H), 7.39 (dd, 4.4, 1.2, 1H), 4.23 (t, 5.2, 2H), 2.41 (dd, 5.2, 2H)
