# Supplementary figures and images for: Study on the Polar Extracts of Dendrobium nobile, D. officinale, D. loddigesii, and Flickingeria fimbriata: Metabolite Identification, Content Evaluation, and Bioactivity Assay
Source: Molecules. 2018 May 15;23(5):1185. doi: 10.3390/molecules23051185 (PMC6099805; doi:10.3390/molecules23051185)

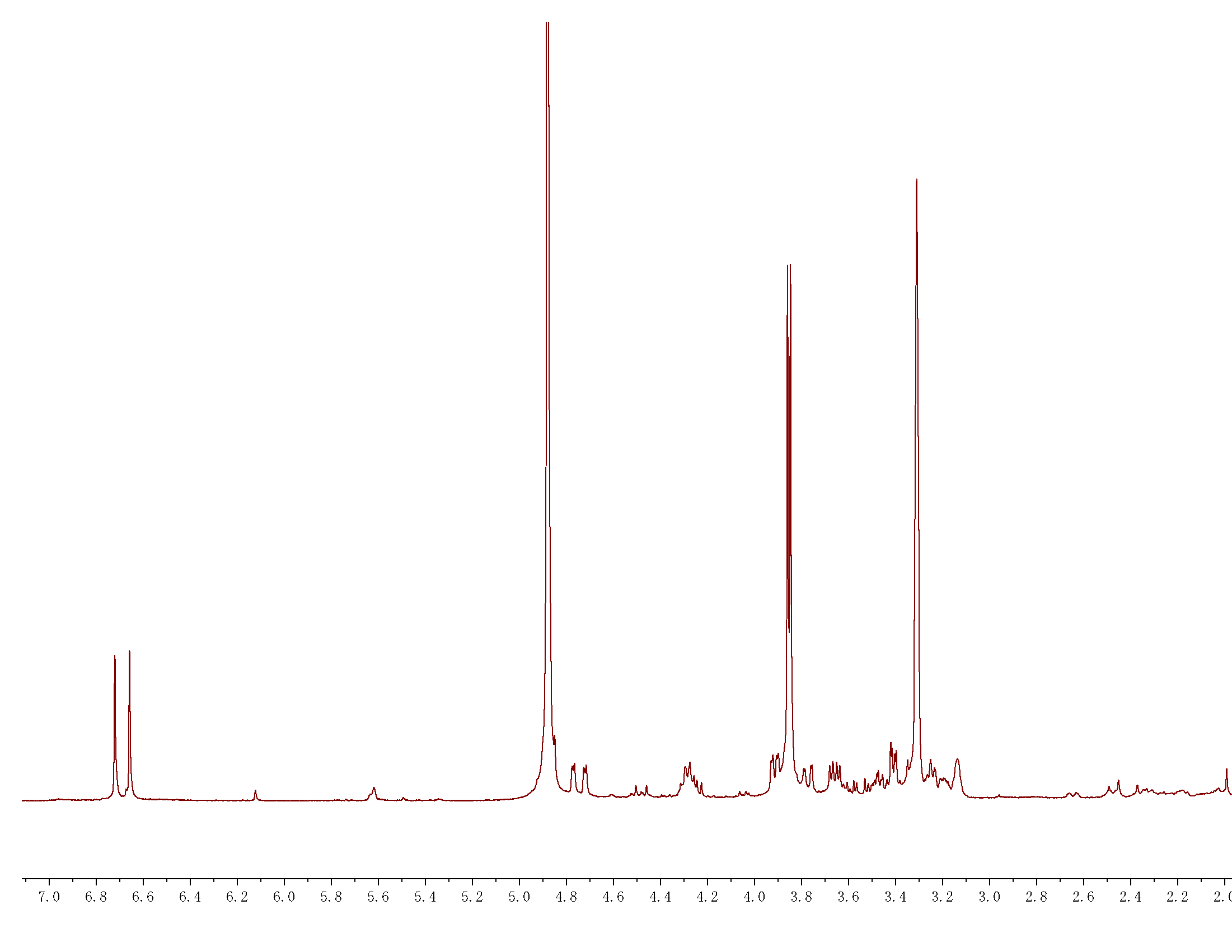

Supplement: Supplementary file 1 [file molecules-23-01185-s001.zip › molecules-287670-supplementary/Supplementary Materials/figures and table in Supplementary Materials/Figure S10.tif]

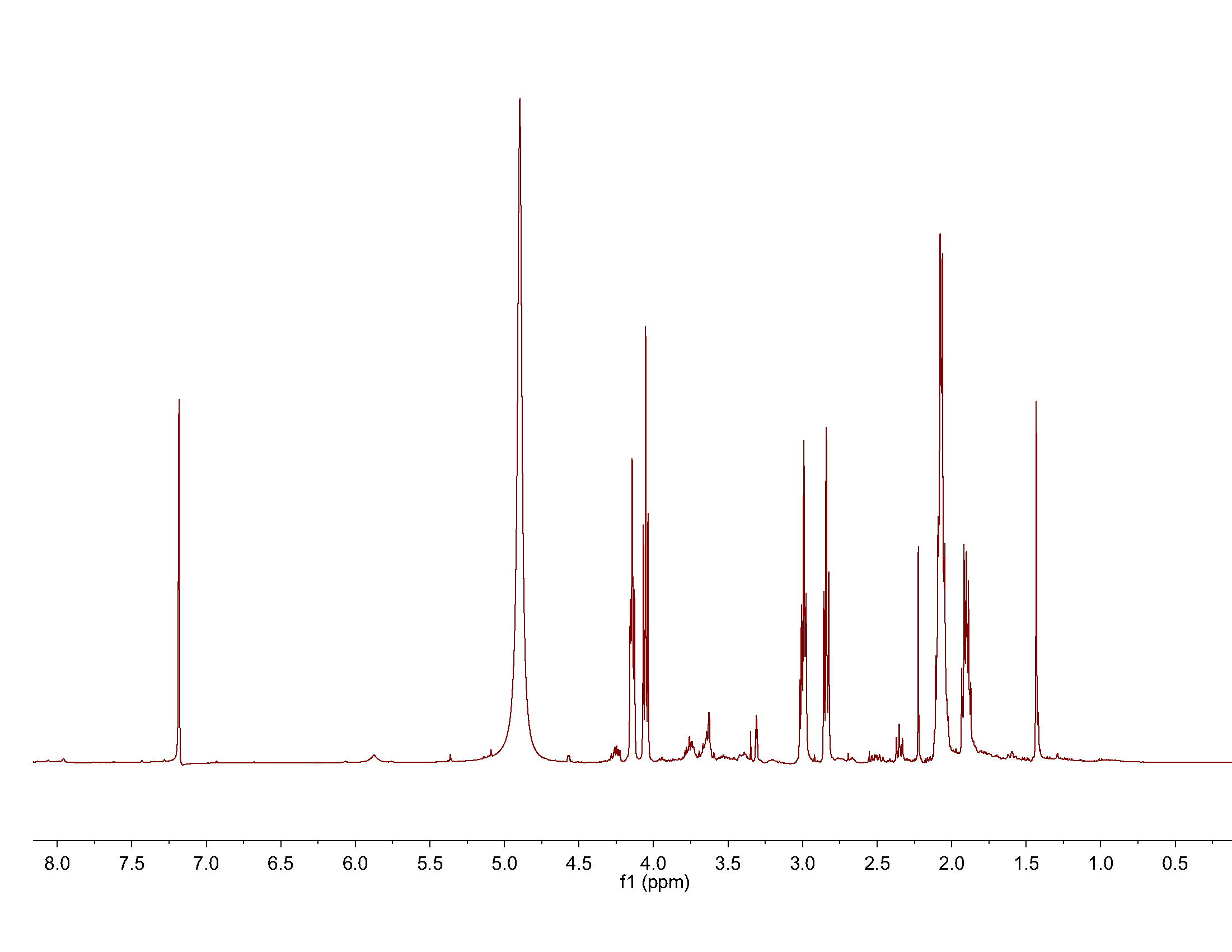

Supplement: Supplementary file 1 [file molecules-23-01185-s001.zip › molecules-287670-supplementary/Supplementary Materials/figures and table in Supplementary Materials/Figure S11.tif]

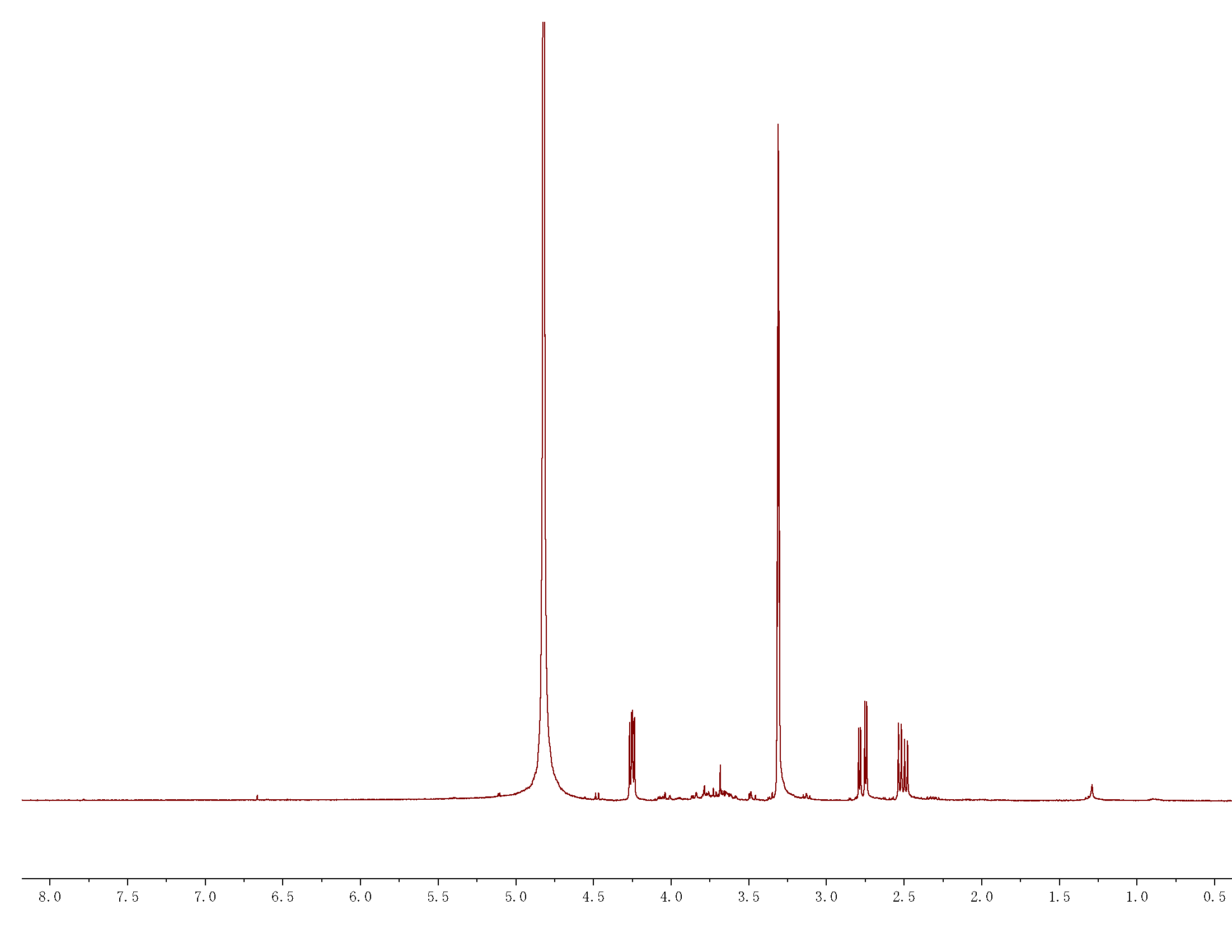

Supplement: Supplementary file 1 [file molecules-23-01185-s001.zip › molecules-287670-supplementary/Supplementary Materials/figures and table in Supplementary Materials/Figure S12.tif]

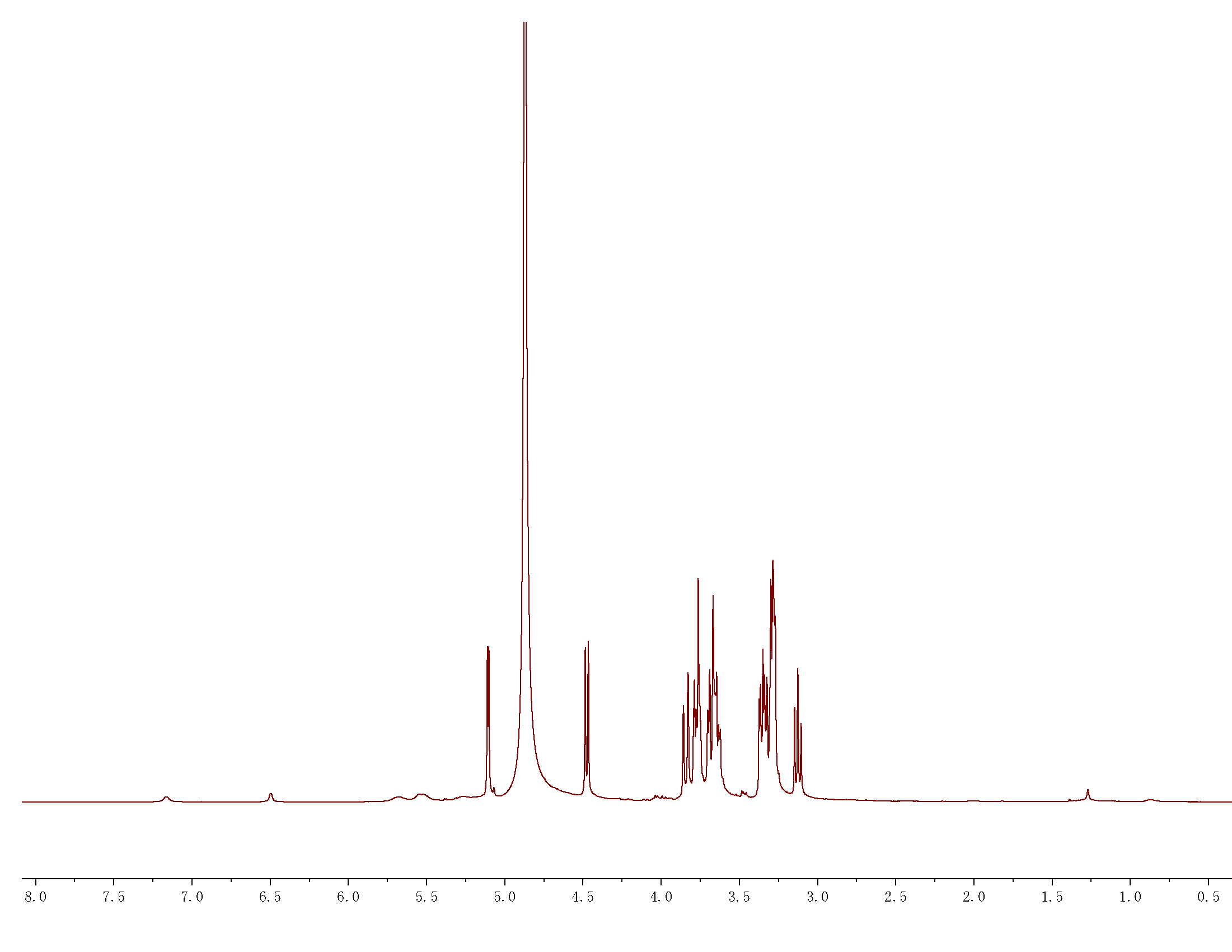

Supplement: Supplementary file 1 [file molecules-23-01185-s001.zip › molecules-287670-supplementary/Supplementary Materials/figures and table in Supplementary Materials/Figure S13.tif]

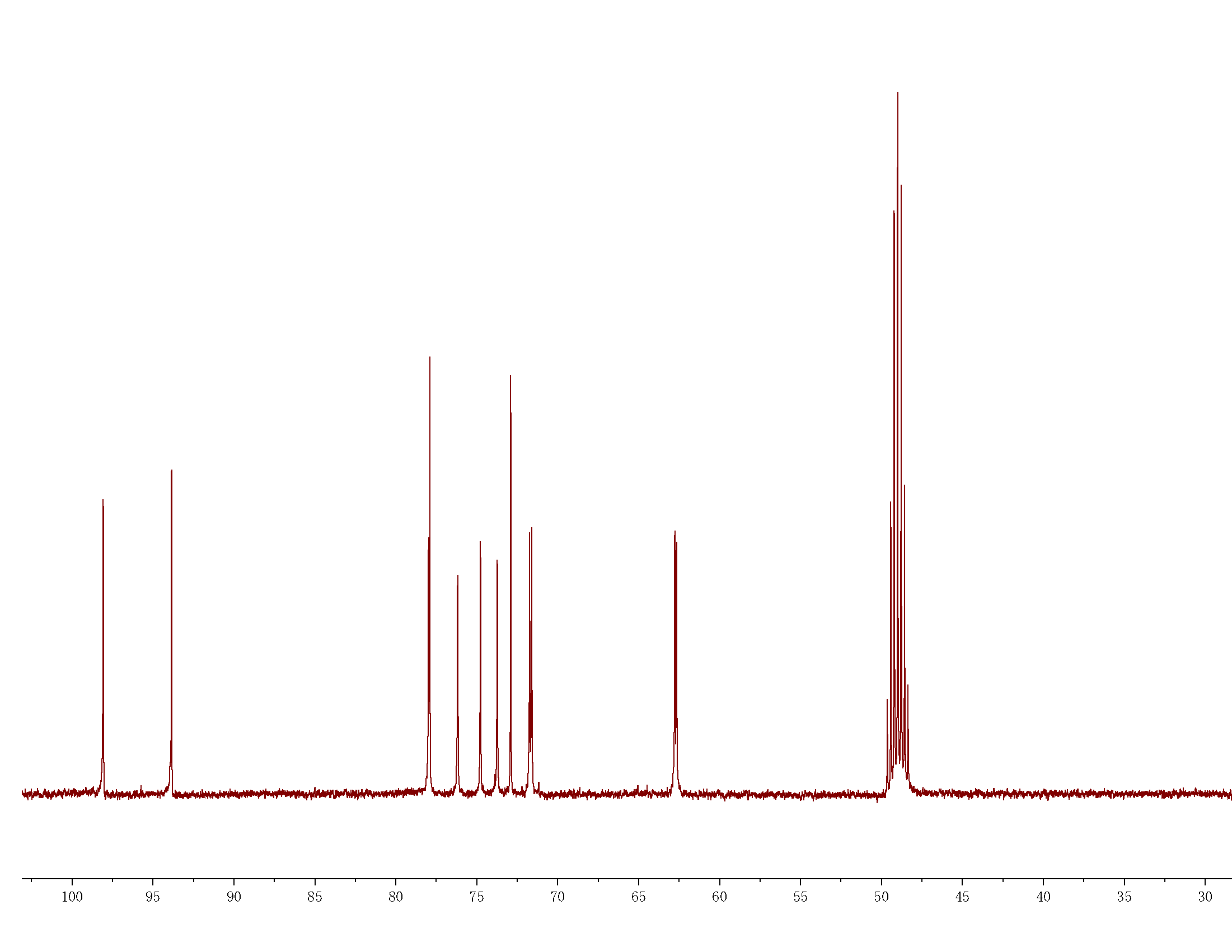

Supplement: Supplementary file 1 [file molecules-23-01185-s001.zip › molecules-287670-supplementary/Supplementary Materials/figures and table in Supplementary Materials/Figure S14.tif]

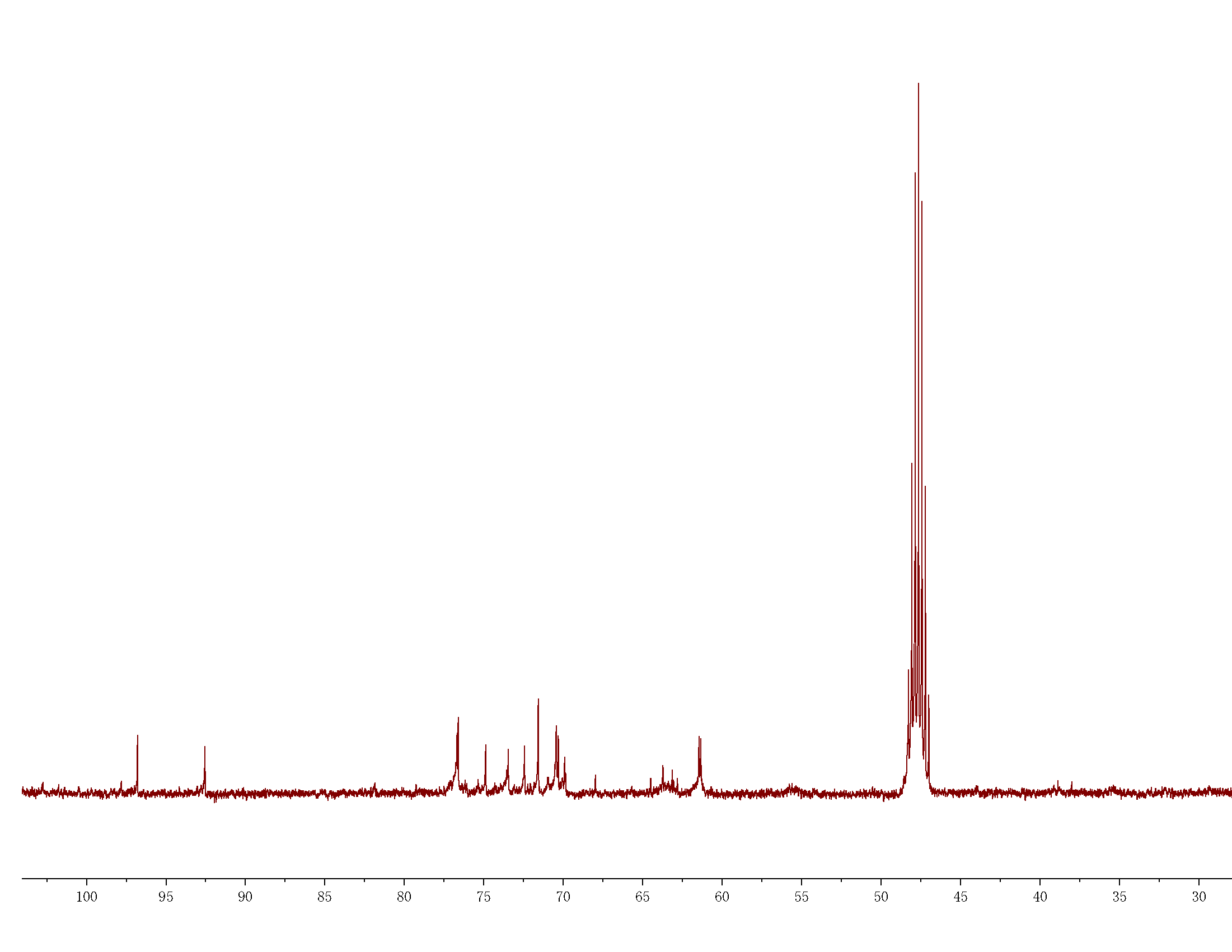

Supplement: Supplementary file 1 [file molecules-23-01185-s001.zip › molecules-287670-supplementary/Supplementary Materials/figures and table in Supplementary Materials/Figure S15.tif]

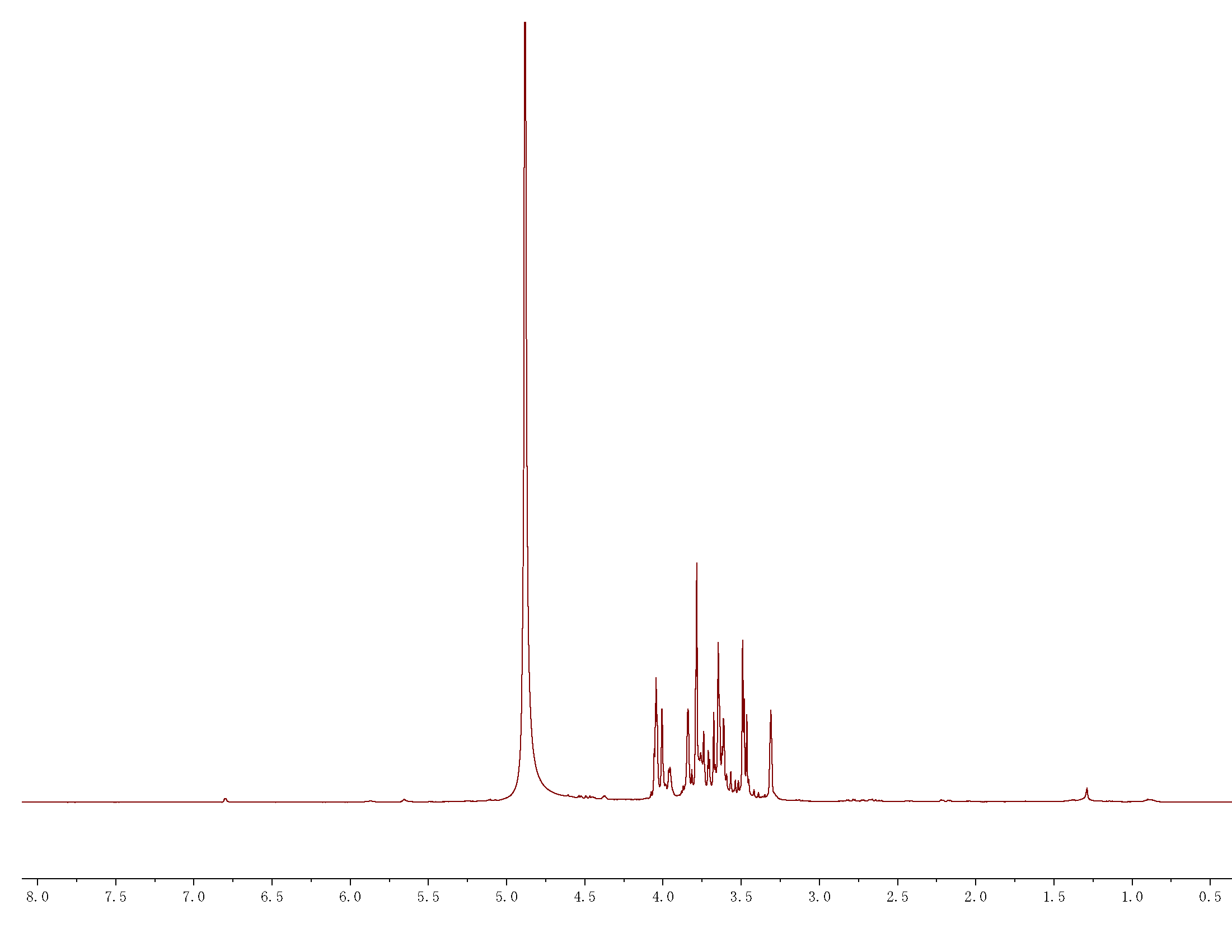

Supplement: Supplementary file 1 [file molecules-23-01185-s001.zip › molecules-287670-supplementary/Supplementary Materials/figures and table in Supplementary Materials/Figure S16.tif]

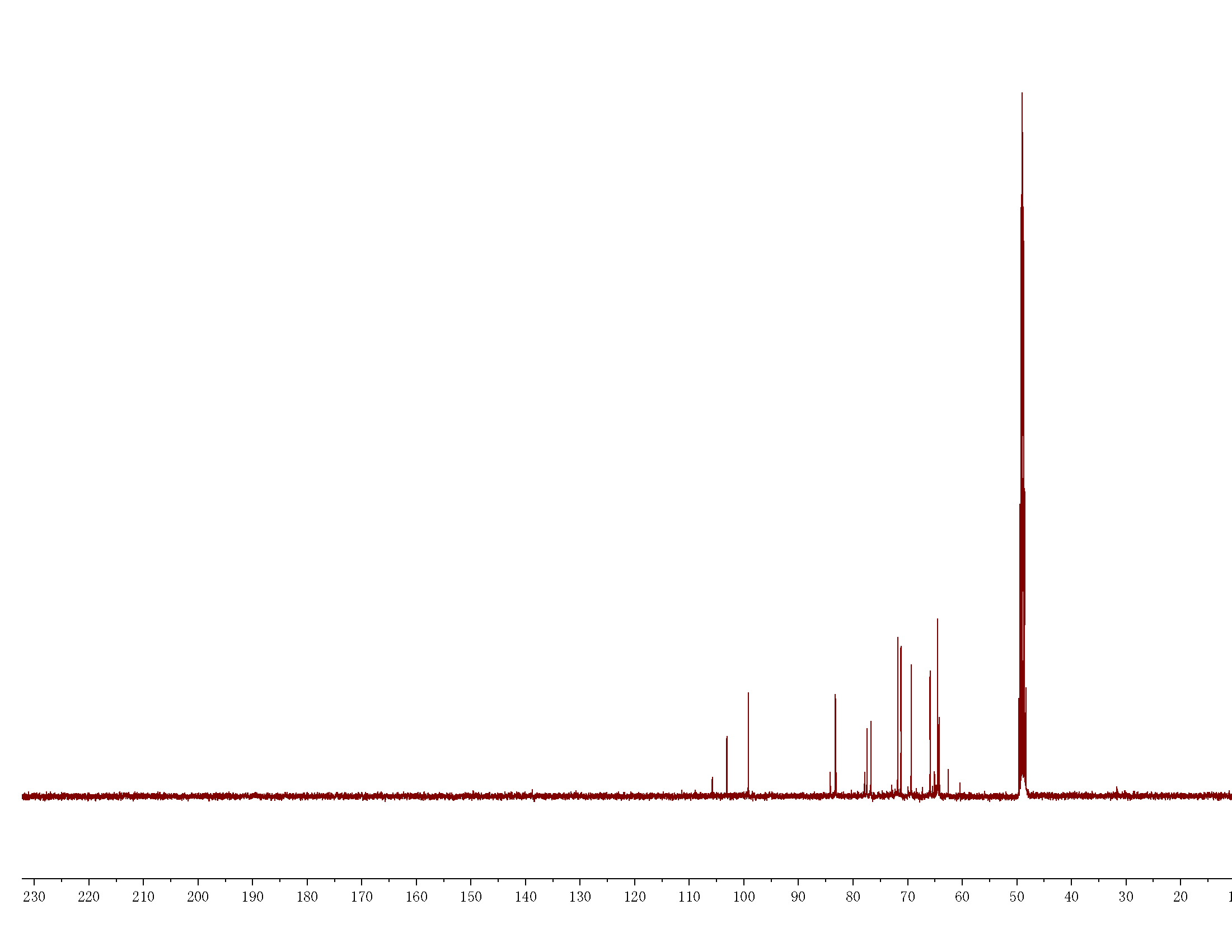

Supplement: Supplementary file 1 [file molecules-23-01185-s001.zip › molecules-287670-supplementary/Supplementary Materials/figures and table in Supplementary Materials/Figure S17.tif]

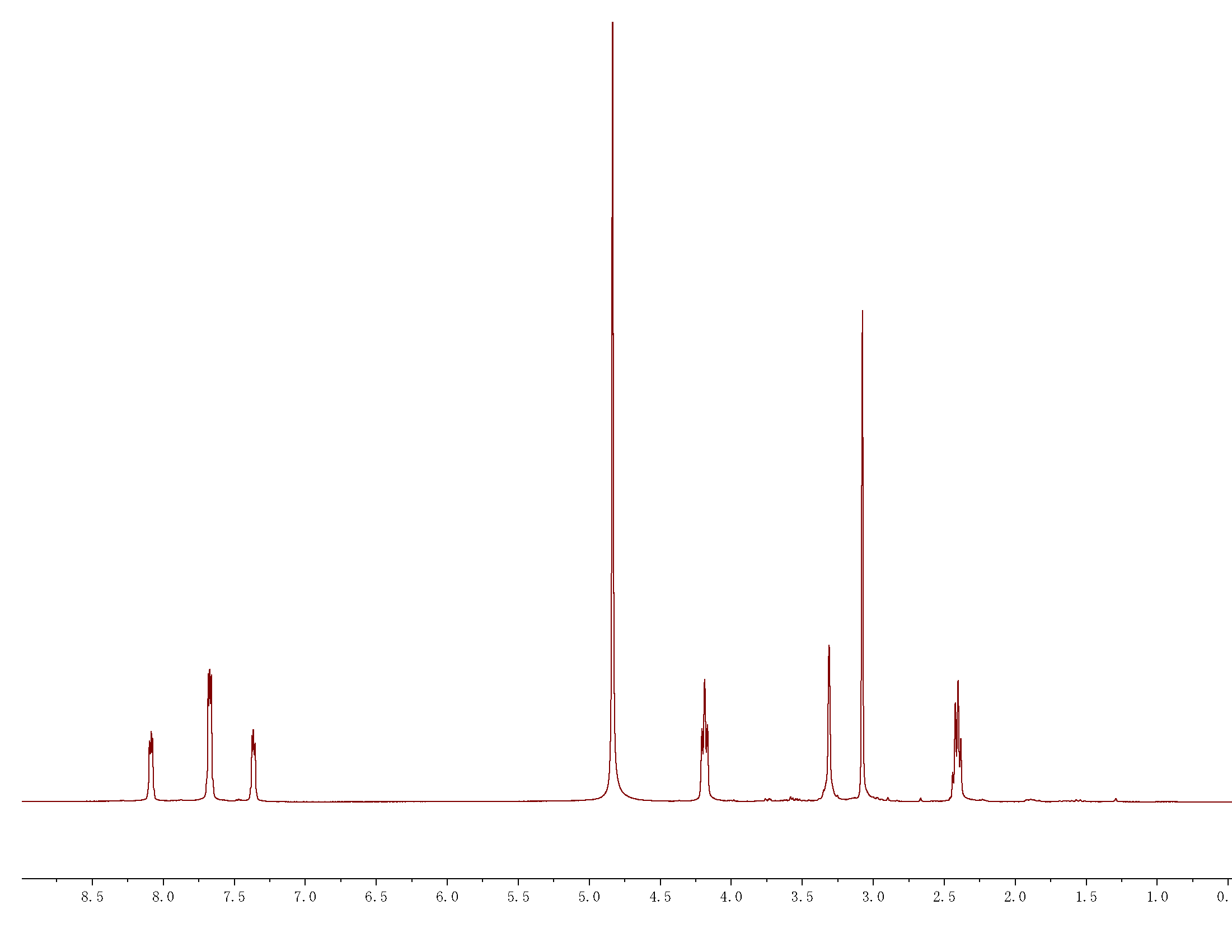

Supplement: Supplementary file 1 [file molecules-23-01185-s001.zip › molecules-287670-supplementary/Supplementary Materials/figures and table in Supplementary Materials/Figure S18.tif]

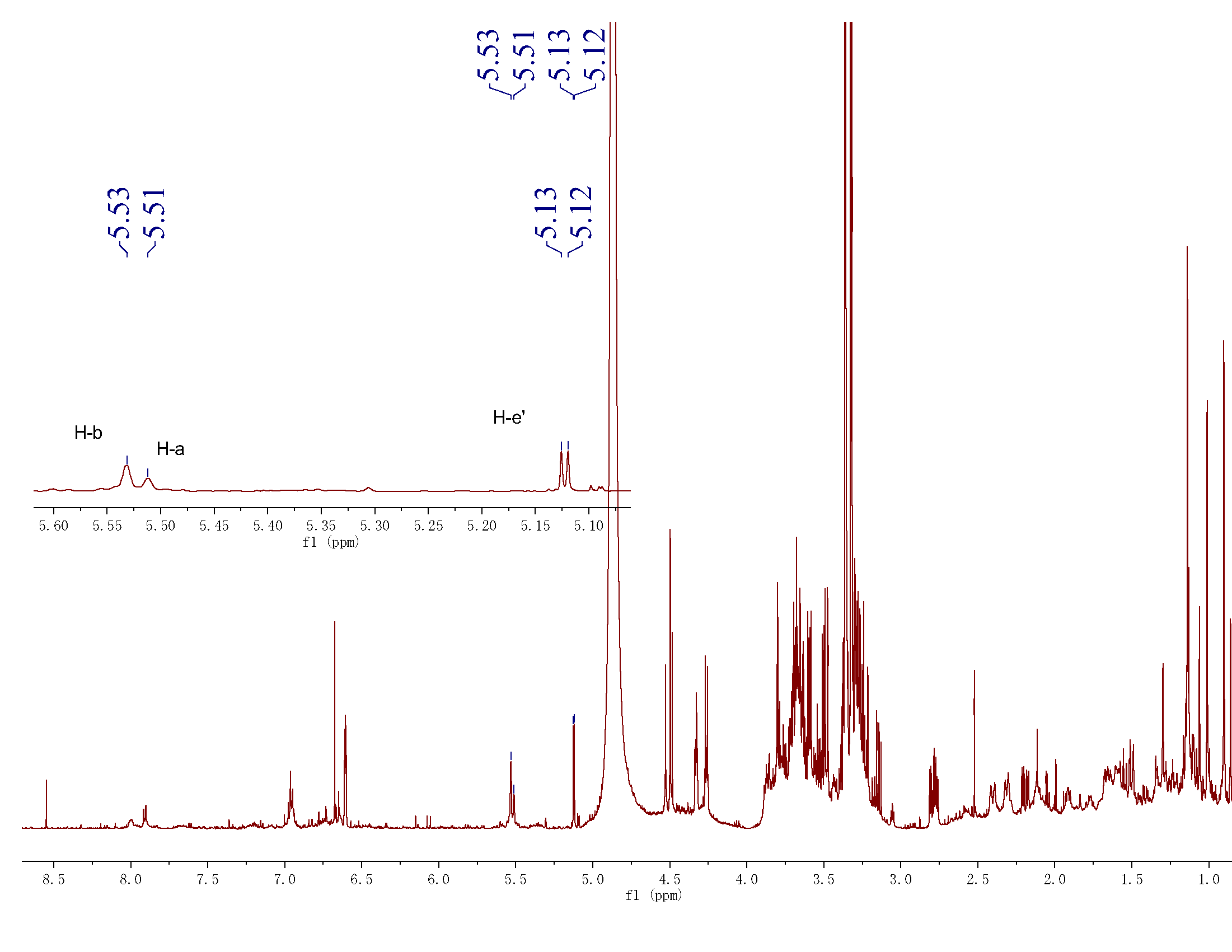

Supplement: Supplementary file 1 [file molecules-23-01185-s001.zip › molecules-287670-supplementary/Supplementary Materials/figures and table in Supplementary Materials/Figure S19.tif]

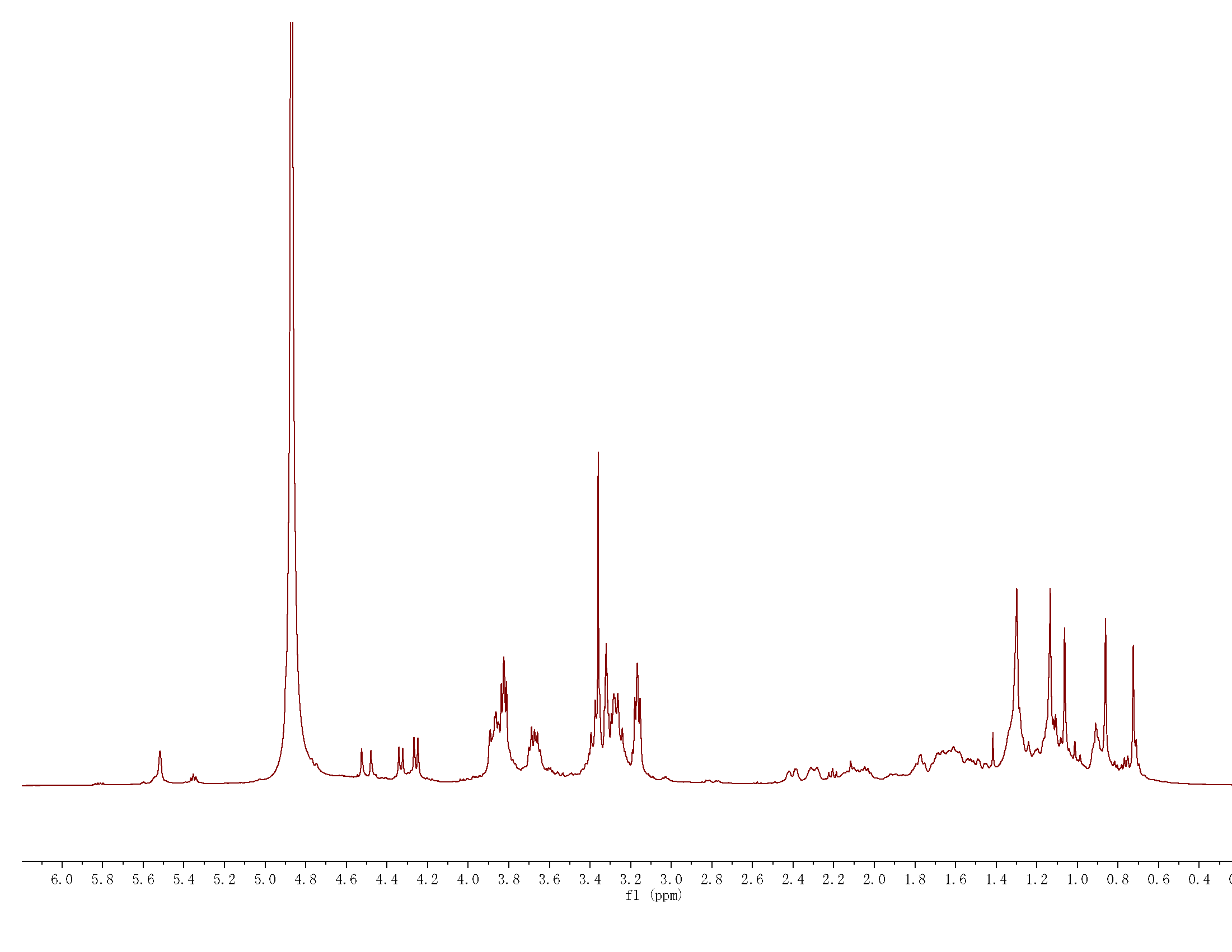

Supplement: Supplementary file 1 [file molecules-23-01185-s001.zip › molecules-287670-supplementary/Supplementary Materials/figures and table in Supplementary Materials/Figure S2.tif]

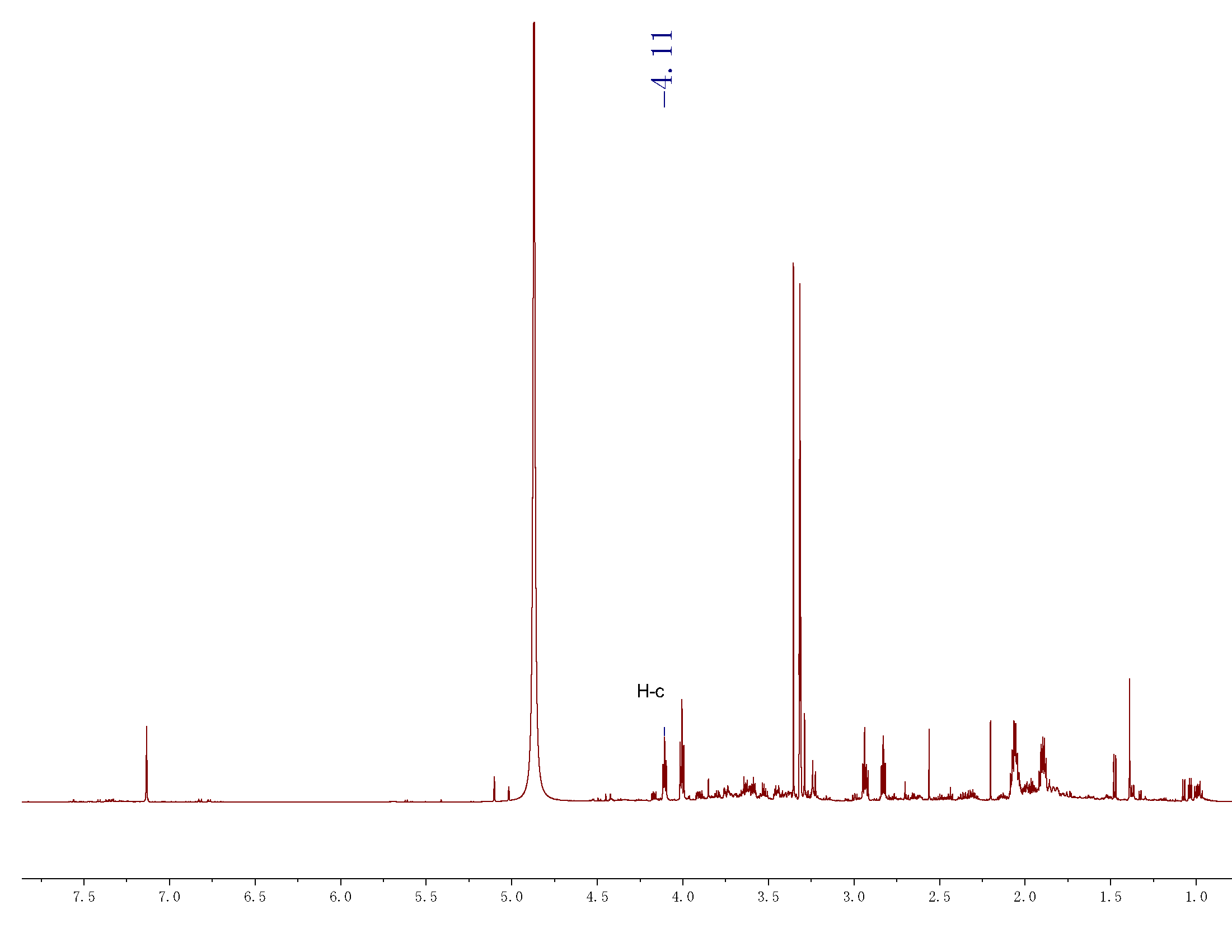

Supplement: Supplementary file 1 [file molecules-23-01185-s001.zip › molecules-287670-supplementary/Supplementary Materials/figures and table in Supplementary Materials/Figure S20.tif]

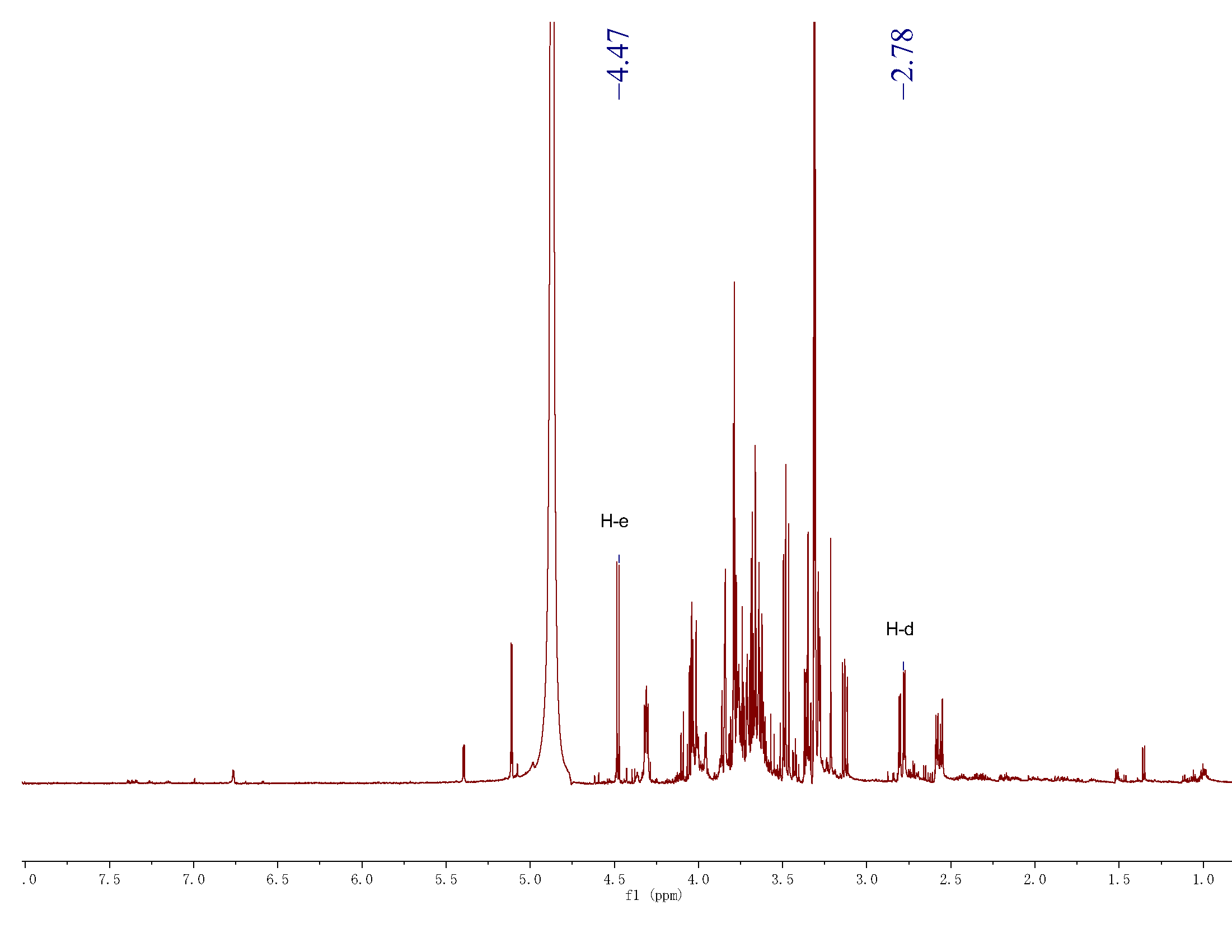

Supplement: Supplementary file 1 [file molecules-23-01185-s001.zip › molecules-287670-supplementary/Supplementary Materials/figures and table in Supplementary Materials/Figure S21.tif]

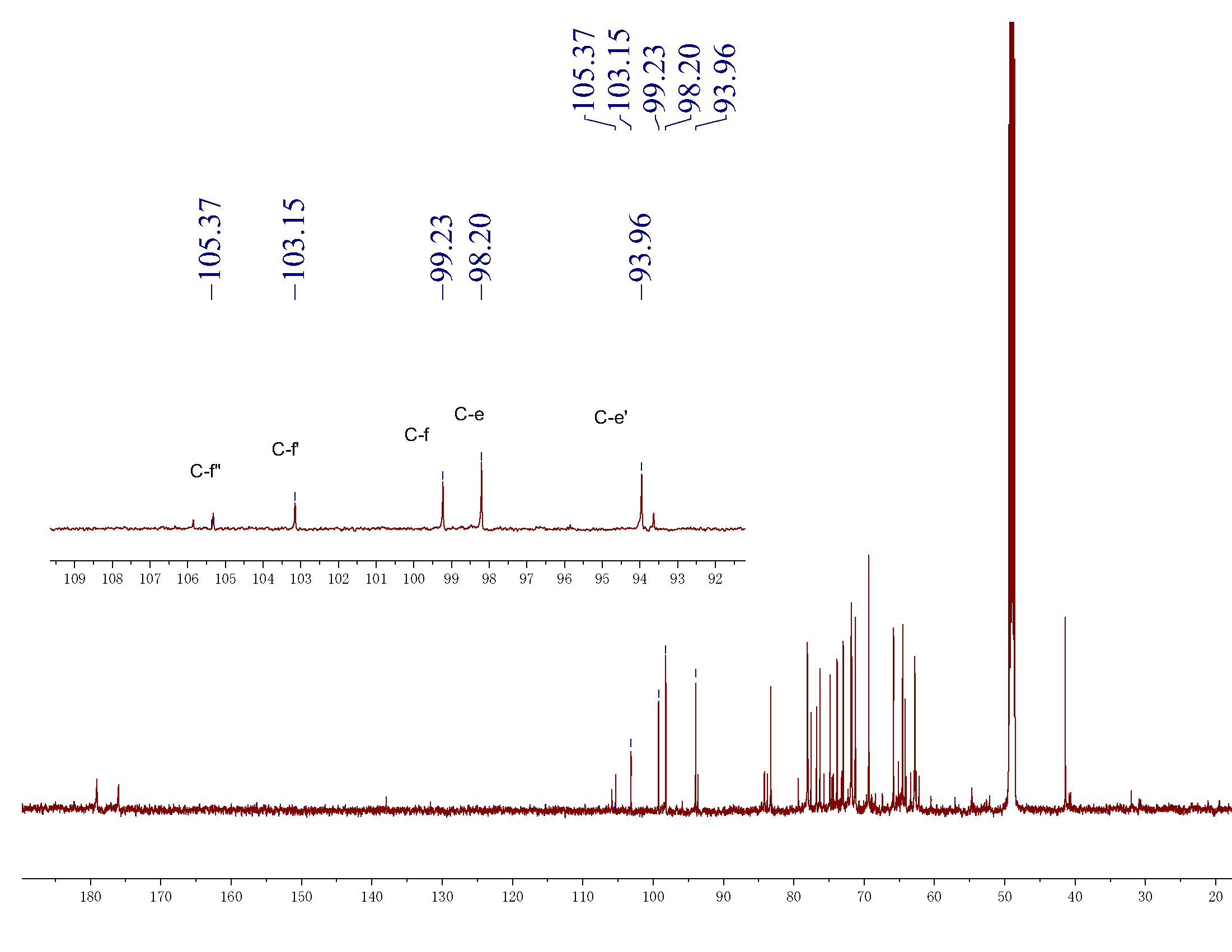

Supplement: Supplementary file 1 [file molecules-23-01185-s001.zip › molecules-287670-supplementary/Supplementary Materials/figures and table in Supplementary Materials/Figure S22.tif]

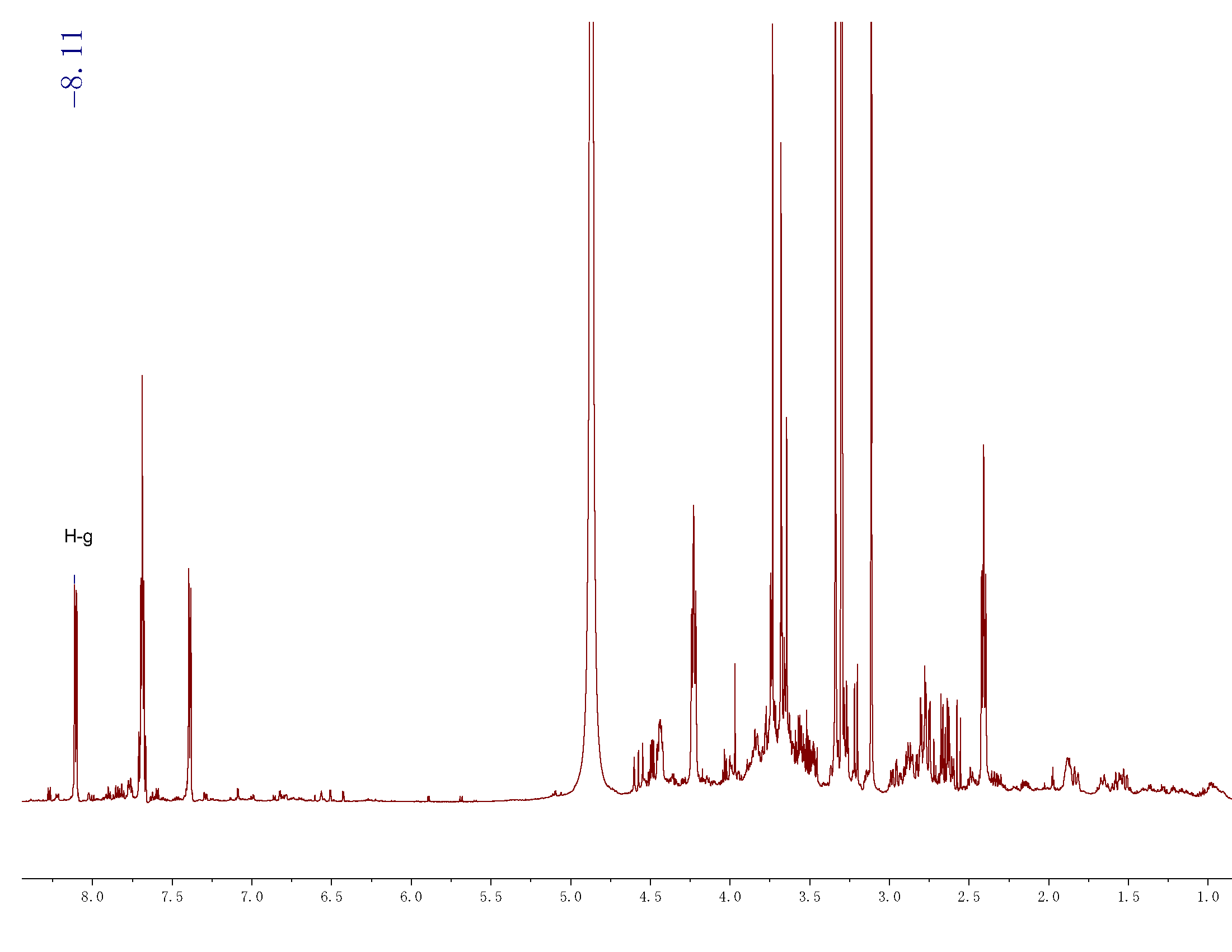

Supplement: Supplementary file 1 [file molecules-23-01185-s001.zip › molecules-287670-supplementary/Supplementary Materials/figures and table in Supplementary Materials/Figure S23.tif]

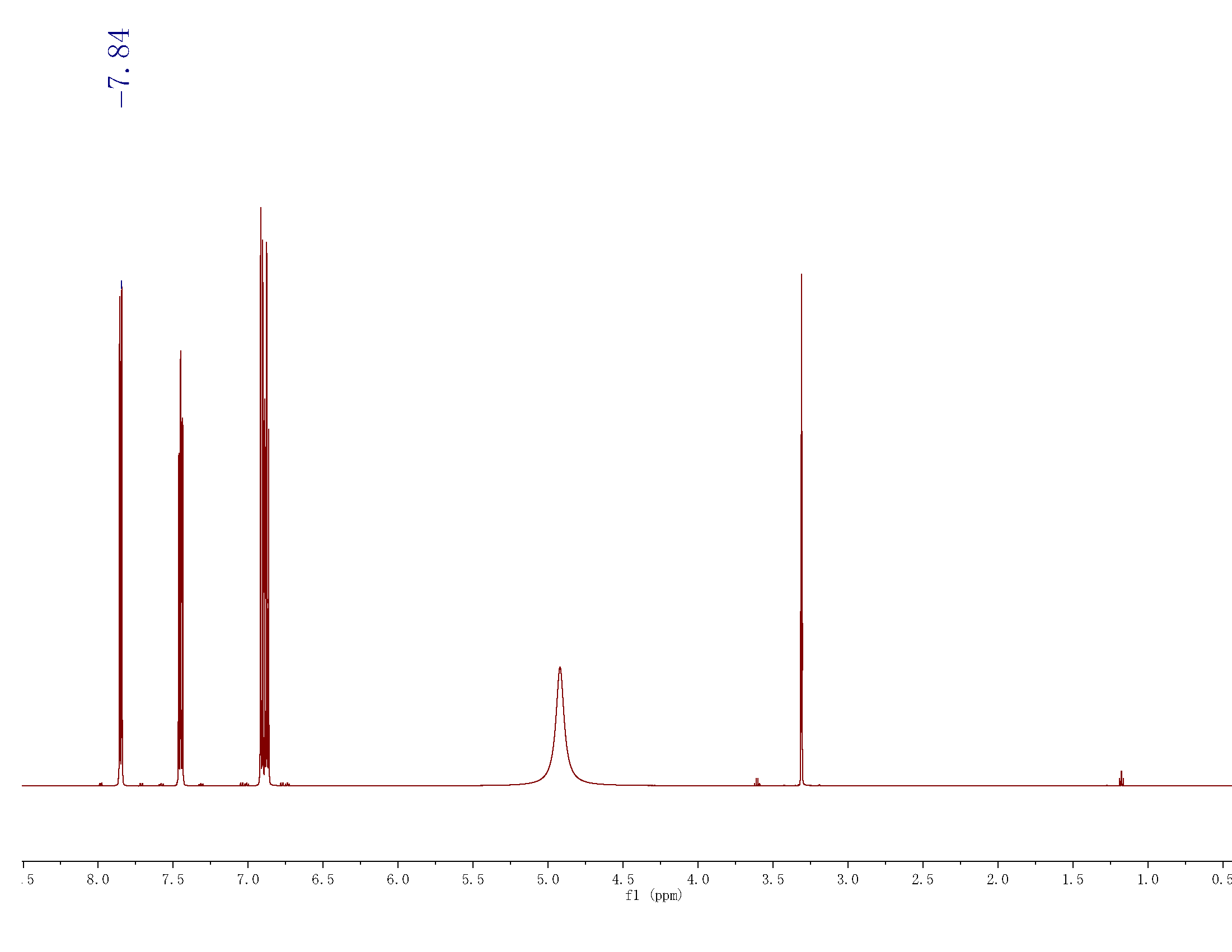

Supplement: Supplementary file 1 [file molecules-23-01185-s001.zip › molecules-287670-supplementary/Supplementary Materials/figures and table in Supplementary Materials/Figure S24.tif]

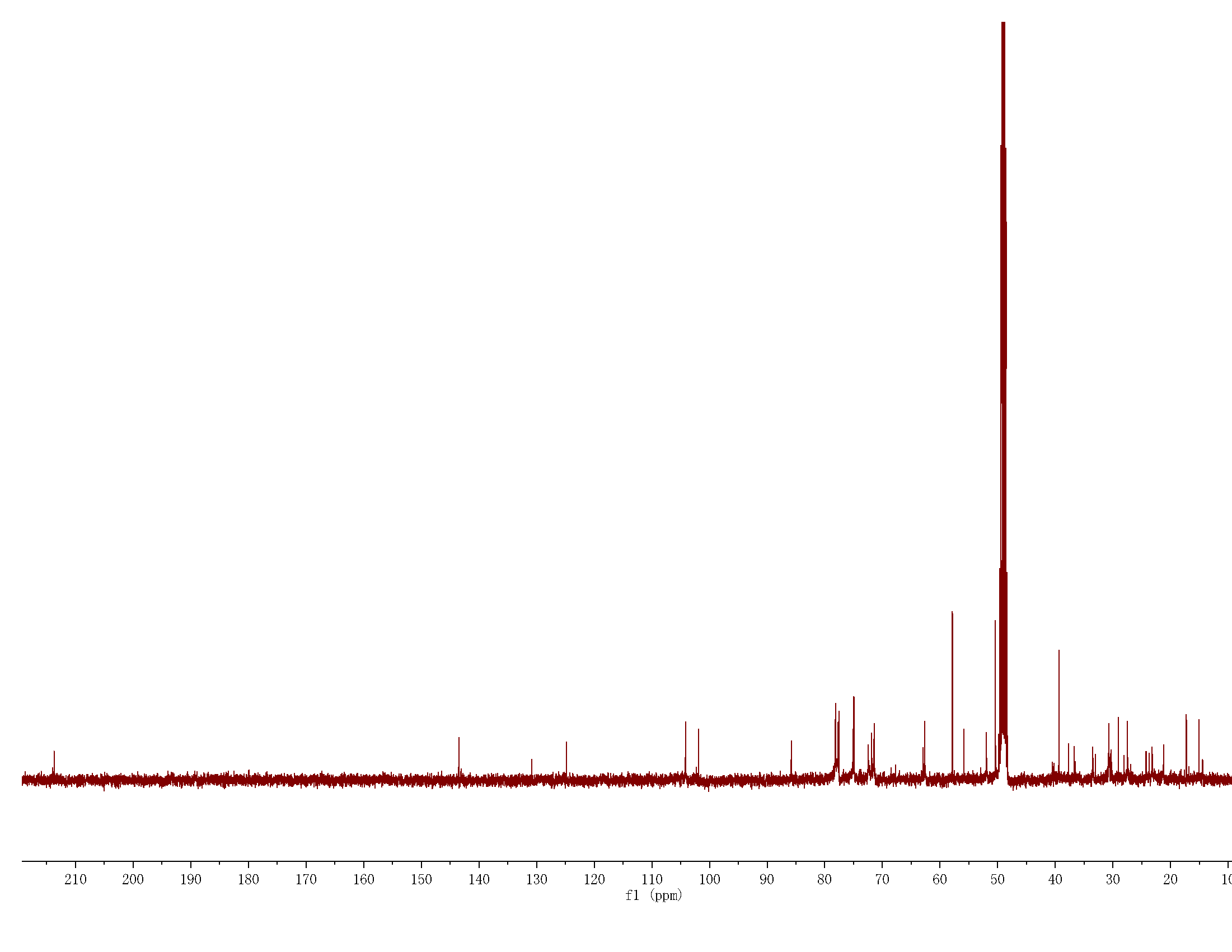

Supplement: Supplementary file 1 [file molecules-23-01185-s001.zip › molecules-287670-supplementary/Supplementary Materials/figures and table in Supplementary Materials/Figure S3.tif]

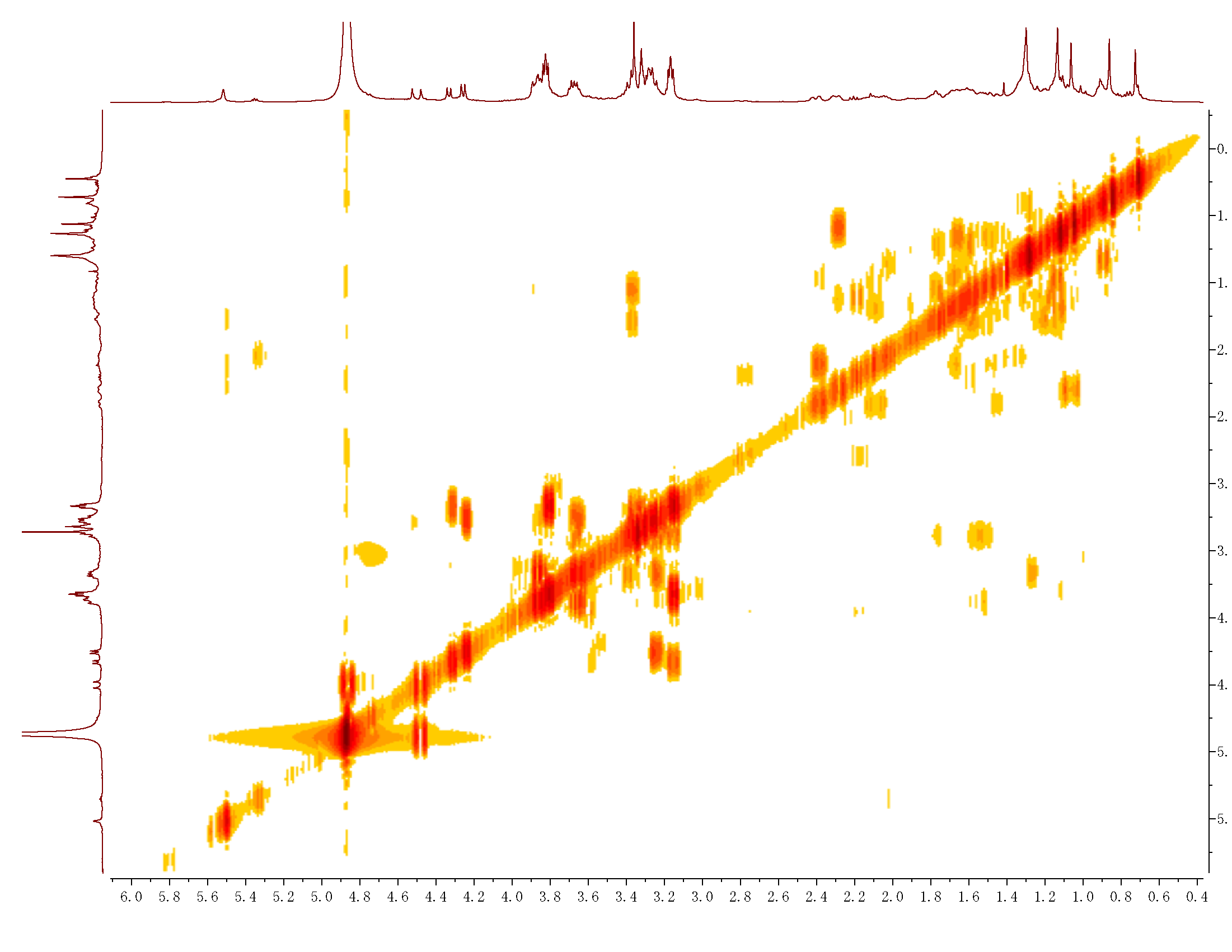

Supplement: Supplementary file 1 [file molecules-23-01185-s001.zip › molecules-287670-supplementary/Supplementary Materials/figures and table in Supplementary Materials/Figure S4.tif]

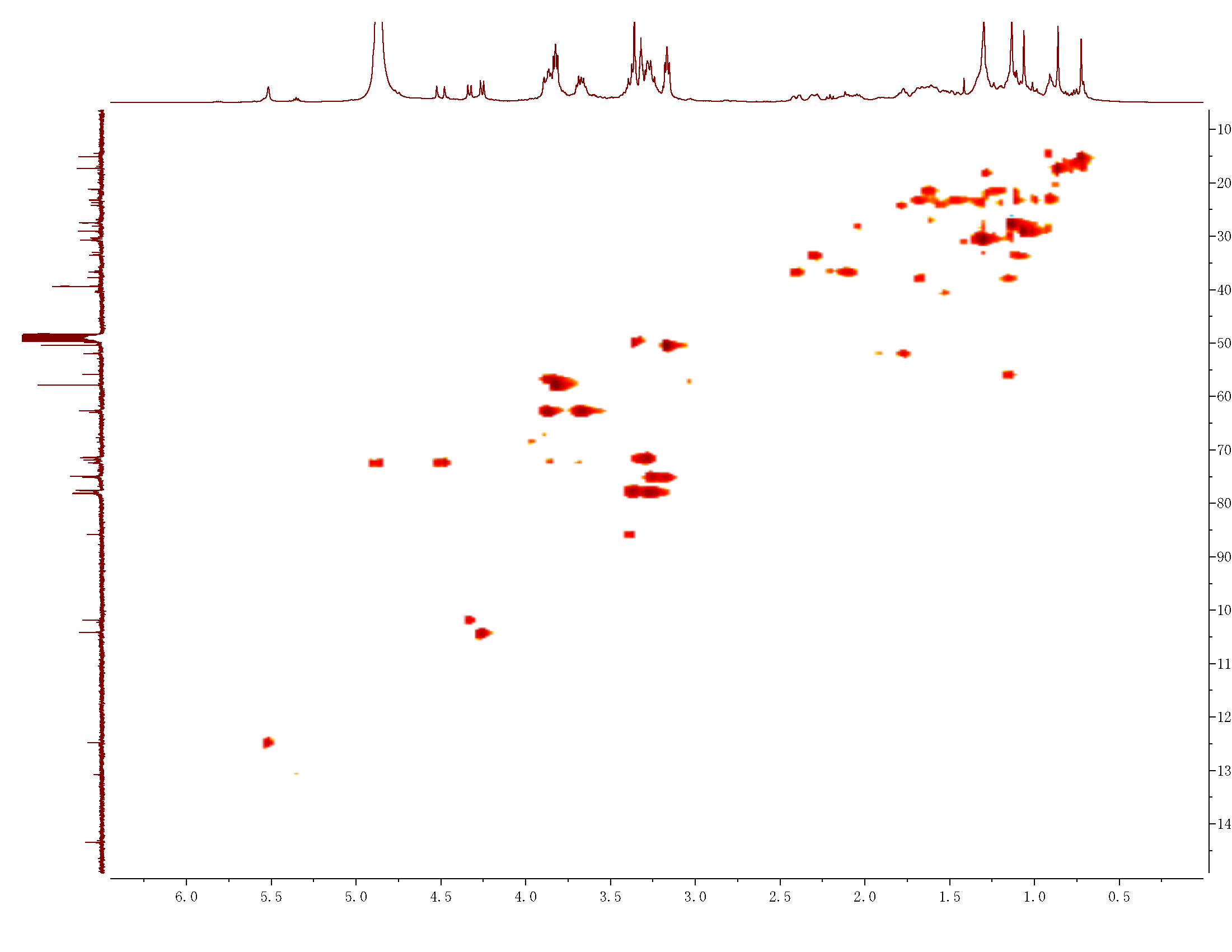

Supplement: Supplementary file 1 [file molecules-23-01185-s001.zip › molecules-287670-supplementary/Supplementary Materials/figures and table in Supplementary Materials/Figure S5.tif]

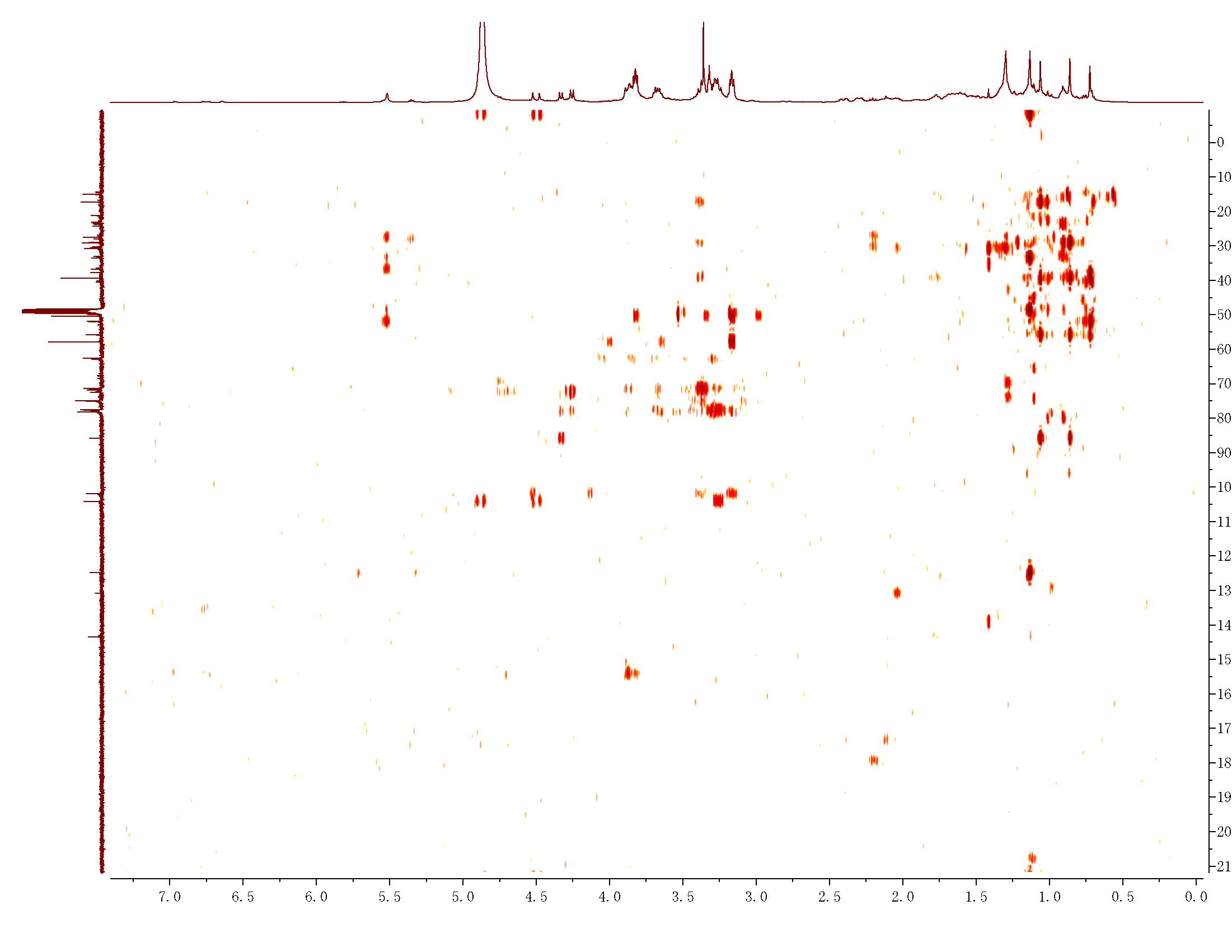

Supplement: Supplementary file 1 [file molecules-23-01185-s001.zip › molecules-287670-supplementary/Supplementary Materials/figures and table in Supplementary Materials/Figure S6.tif]

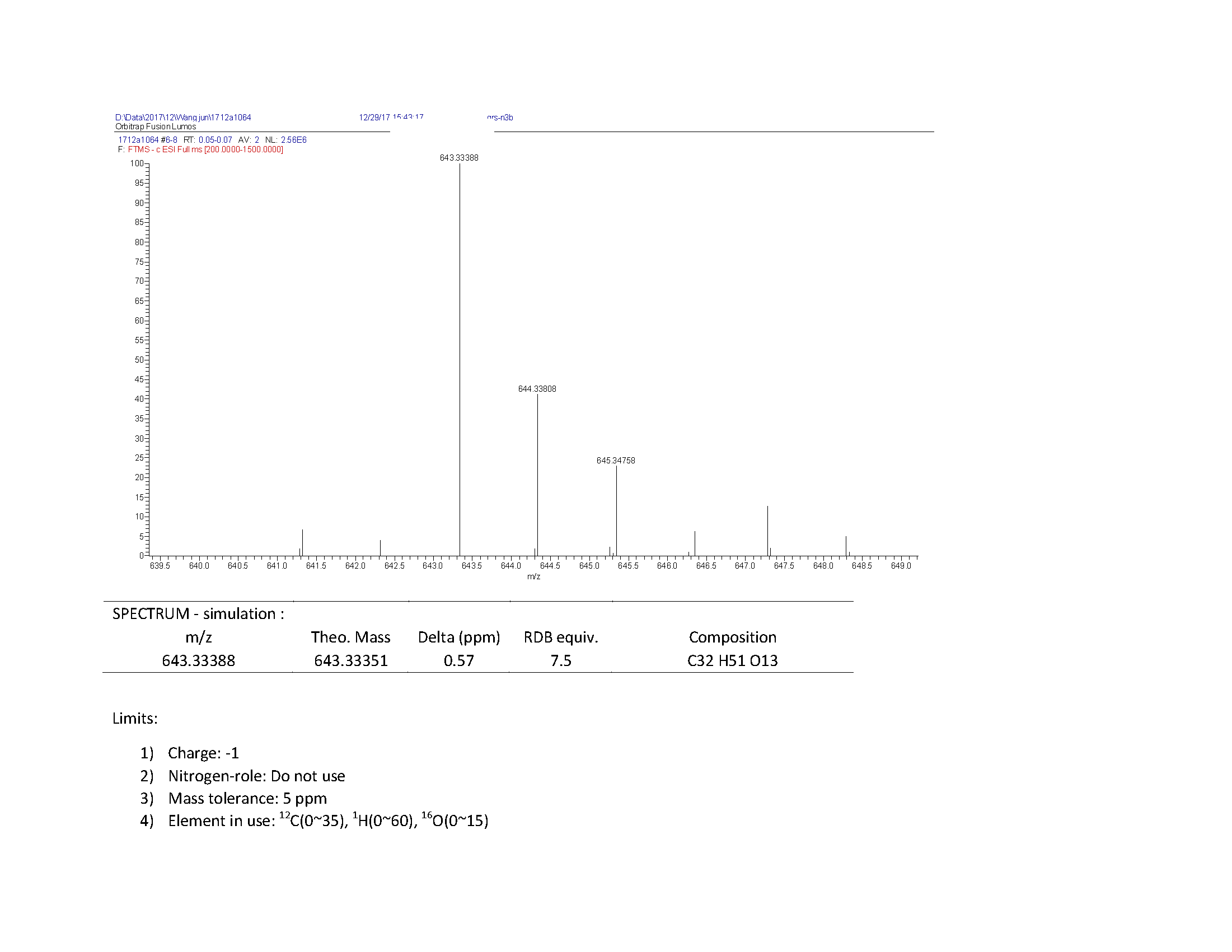

Supplement: Supplementary file 1 [file molecules-23-01185-s001.zip › molecules-287670-supplementary/Supplementary Materials/figures and table in Supplementary Materials/Figure S7.tif]

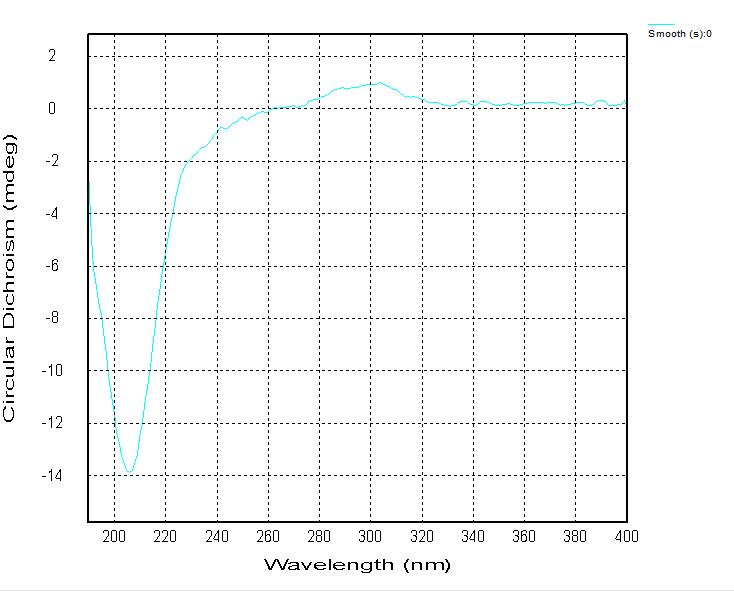

Supplement: Supplementary file 1 [file molecules-23-01185-s001.zip › molecules-287670-supplementary/Supplementary Materials/figures and table in Supplementary Materials/Figure S8.jpg]

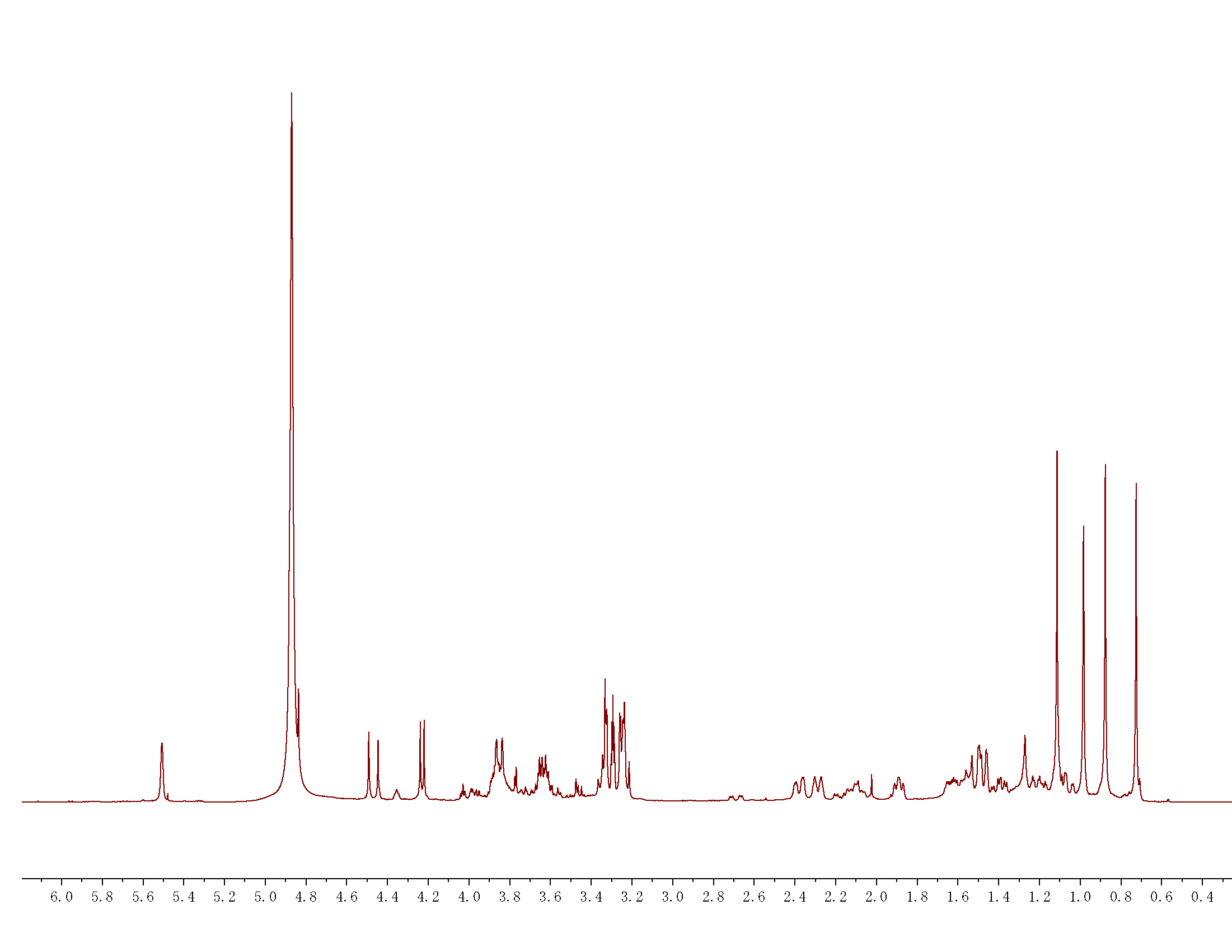

Supplement: Supplementary file 1 [file molecules-23-01185-s001.zip › molecules-287670-supplementary/Supplementary Materials/figures and table in Supplementary Materials/Figure S9.tif]
